# Observation of the amplified transverse thermoelectric signals by reduced-dimensionality transport anisotropy

**DOI:** 10.1038/s41467-026-77569-y

**Published:** 2026-09-08

**Authors:** Honghui Wang, Bin He, Xiaolong Feng, Haihua Hu, Ralf Koban, Walter Schnelle, Erjian Cheng, Yu Pan, Xiaoyuan Zhou, Claudia Felser

**Affiliations:** 1 Max Planck Institute for Chemical Physics of Solids, Dresden, Germany; 2 College of Physics and Center of Quantum Materials & Devices of Chongqing University, Chongqing, P. R. China; 3 Shenyang National Laboratory for Materials Science, Institute of Metal Research, Chinese Academy of Science, Shenyang, China; 4 School of Materials Science and Engineering, University of Science and Technology of China, Shenyang, China

**Keywords:** Thermoelectrics, Condensed-matter physics

## Abstract

Transverse thermoelectric devices offer simplified geometries with flexible designs for next-generation cryogenic technologies. However, mechanisms on geometrical amplification of transverse thermoelectric effects remain elusive. Here we establish a general framework linking reduced dimensionality with its geometrical anisotropy to enhanced transverse thermoelectric responses. Taking quasi-one-dimensional Li_0.9_Mo_6_O_17_ as a model platform, we combine theoretical modeling with systematic experiments to reveal extraordinarily large Nernst and Ettingshausen signals, reaching 11,430 μV/K (25 K, 9 T) and 0.0063 K · m/A (43.1 K, 9 T), respectively, together with a high power factor of 814 μW cm^−1^ K^−2^ at 25 K and 9 T. Dimensional reduction amplifies the Seebeck-driven electric field and enhances the reduced mobility through anisotropic Fermi surface geometry. These effects yield the transverse thermoelectric response far exceeding those of higher-dimensional systems with even ultrahigh carrier mobilities. Our findings highlight enhanced geometrical anisotropy as a powerful principle of design for engineering high-performance transverse thermoelectric systems.

## Abstract (web summary)

Dimensional reduction amplifies transverse thermoelectricity by reshaping Fermi surfaces and amplifying Seebeck-driven fields. In quasi-one-dimensional Li_0.9_Mo_6_O_17_, this enables strong Nernst and Ettingshausen responses.

## Introduction

Thermoelectric (TE) effects enable the direct interconversion of heat and electricity, offering an attractive pathway for solid-state refrigeration and energy harvesting. High-efficiency devices require not only a large figure of merit but also substantial output power, in which sizeable thermopower is highly preferred. Strategies, including resonant levels, band convergence and dimensional reduction, boost the Seebeck coefficient through sharpening the energy dependence of the electronic density of states^1–5^. In metals, however, the Fermi energy greatly exceeds k_B_*T*, resulting in only a minute imbalance in electron-hole symmetry and thereby overshadowing a large net Seebeck response, which limits the cooling efficiency below the liquid-nitrogen temperature (~77 K)^6^. Fortunately, transverse TE effects, including the Nernst and Ettingshausen effects, are often enhanced in the presence of carrier compensation, bringing the renaissance for metals as good TE materials^7–16^. Furthermore, high carrier mobility, enabled by suppressed electron-phonon scattering at low temperatures, and phonon drag contributions can further amplify these signals^17–20^, positioning transverse TE effects as promising alternatives for cryogenic cooling.

**Fig. 1:**
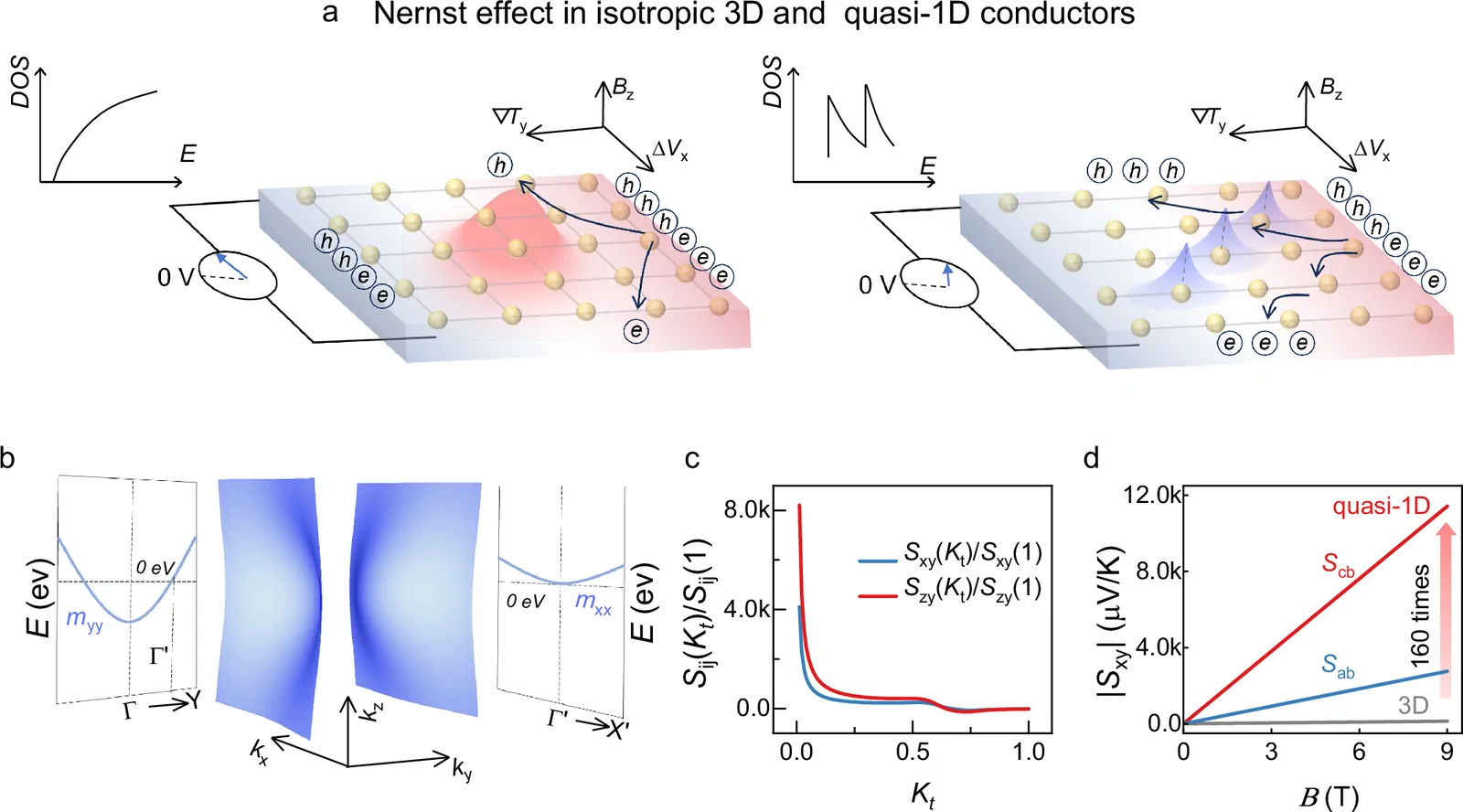
Conceptual framework for dimensional reduction-driven enhancement of the Nernst effect. **a** A schematic illustration of the Nernst effect in an isotropic 3D (left) and a quasi-1D conductor (right). When the same heat flux is applied along the *y*-axis and the same orthogonal magnetic field along the *z*-axis, the resulting Nernst voltage along the *x*-axis differs significantly between quasi-one-dimensional and three-dimensional systems. In 3D systems, atomic orbital overlap (red wave packet) is comparable along all crystallographic axes, yielding nearly isotropic effective masses (*m*_xx_^*^ ≈ *m*_yy_^*^) and mobilities (*μ*_xx_  ≈ *μ*_yy_ ≈ *μ*_H_). By contrast, quasi-1D conductors (conducting chains along the *y*-axis) display pronounced anisotropy: strong orbital hybridization dominates along the chain direction, whereas inter-chain coupling remains weak and yields strong electron localization (blue wave packets). This anisotropic coupling landscape leads to large disparities in effective mass and thus direction-dependent carrier mobility. Consequently, the electronic entropy *δ* (*δ* ~ m^*^) is larger for inter-chain transport in quasi-1D systems than in 3D ones^33^, leaving a greater residual charge. Together with an enlarged Seebeck-driven electric field arising from the sharp density of states (top left; continuous energy-dependent density of states in 3D conductors and nearly δ-shaped energy-dependent density of states in quasi-1D conductors), these factors markedly amplify the Nernst response. **b** Schematic of the strongly anisotropic effective mass distribution associated with a corrugated open Fermi surface in quasi-1D conductors. The Γ-Y direction corresponds to the conducting chain, while Γ`-X` denotes the interchain direction. **c** Calculated Nernst thermopower within the constant relaxation-time approximation (CRTA) for *t*_x_ = 2*t*_z_. The Nernst thermopower increases with decreasing *K*_t_ (*K*_t_ ∝ *K*_m_, *K*_m_ = *m*_xx/__zz_/*m*_yy_). For the same longitudinal Seebeck coefficient *S*_yy_, *S*_zy_ exhibits a stronger enhancement than *S*_xy_, demonstrating that, beyond the Seebeck coefficient, transport anisotropy through the reduced-mobility term can provide an additional and substantial enhancement of the Nernst response. **d** Magnetic-field dependence of the Nernst thermopower in LMO at 25 K. Upon incorporating the contribution from dimensional reduction, the Nernst thermopower *S*_ab_ and *S*_cb_ are enhanced by factors exceeding 22 and 160, respectively.

In transverse TE geometry, the thermal gradient, electric current (or voltage), and magnetic field are mutually orthogonal (see Fig. 1a). This geometric decoupling of thermal and charge transport simplifies device structures^21,22^ and introduces additional degrees of freedom for material optimization that are absent in longitudinal configurations. Large Nernst responses have been reported in various correlated and topological materials^7–22^, which are contributed by the phonon drag-induced high partial Seebeck coefficients (*S*_xx_^h^; *S*_xx_^e^) as well as the high mobilities. Additional underlying mechanisms between transverse geometry and enhanced TE effects, particularly in highly anisotropic systems, remain insufficiently explored. Further enhancement of the Nernst thermopower naturally requires higher partial Seebeck coefficients. Quasi-one-dimensional (quasi-1D) conductors intrinsically host exotic electronic density of states and strongly anisotropic open Fermi surfaces, which may profoundly reshape transverse TE responses. Developing a systematic framework that links quasi-1D electronic anisotropy with macroscopic TE performance is therefore crucial for advancing both fundamental understanding and device design. In this work, we identify enhanced anisotropy as a guiding design principle for amplifying transverse TE responses. By combining theoretical modeling with experimental investigation of bulk quasi-1D Li_0.9_Mo_6_O_17_ (LMO), we show that low-dimensional structures not only generate larger partial Seebeck coefficients than those in higher-dimensional systems but also enhance the reduced mobility ($\tilde{\mu }$) through introducing anisotropic Fermi surface geometry. These effects act synergistically to yield large Nernst and Ettingshausen signals, exceeding state-of-the-art transverse TE materials. These findings establish dimensionality-driven electronic anisotropy as a new framework for transverse TE engineering, opening avenues towards high-performance cryogenic cooling technologies.

## Results

### Theoretical framework for dimensional reduction-amplified transverse TE effects

The thermopower tensor ($\overleftrightarrow{S}={\overleftrightarrow{\sigma }}^{-1}\cdot \overleftrightarrow{\alpha }$) is determined by both the electrical conductivity tensor $\overleftrightarrow{\sigma }$ and the TE conductivity tensor $\overleftrightarrow{\alpha }$. For an isotropic perfectly compensated semimetal (*n*_e_ = *n*_h_ and *μ*_e_ = *μ*_h_), with a temperature gradient applied along the *y* direction, the induced electric voltage measured along the *x* direction, and a magnetic field applied along the *z* direction, the two-carrier Nernst response can be expressed as:^17^

1 $${{{\rm{S}}}}_{{{\rm{xy}}}}=\left({{{{\rm{S}}}}_{{{\rm{xy}}}}}^{{{\rm{e}}}}{\sigma _{{{\rm{yy}}}}}^{{{\rm{e}}}}+{{{{\rm{S}}}}_{{{\rm{yx}}}}}^{{{\rm{h}}}}{\sigma _{{{\rm{yy}}}}}^{{{\rm{h}}}}\right)/{\sigma }_{{{\rm{yy}}}}+1/2{\mu }_{{{\rm{yy}}}}{{{\rm{B}}}}_{{{\rm{z}}}}\left({{{{\rm{S}}}}_{{{\rm{yy}}}}}^{{{\rm{h}}}}-{{{{\rm{S}}}}_{{{\rm{yy}}}}}^{{{\rm{e}}}}\right)$$

where *μ* denotes the carrier mobility and superscripts *e* and *h* refer to electrons and holes, respectively (Supplementary material, Note I). This expression consists of two principal contributions: a diffusive term and an ambipolar term. The diffusive term involves the off-diagonal thermopowers of electrons and holes and scales as *tanΘ*/*ɛ*, where *tanΘ* is the Hall angle, and *ɛ* is the Fermi energy. In highly conductive metals, the combination of a small Hall angle and a large Fermi energy renders this contribution negligible. The ambipolar term, which is coupled to the Seebeck electric field, typically dominates in topological semimetals^7–20^ because high mobility bands coexist with large partial diffusion (*S*_yy_^h^ ≈ −*S*_yy_^e^ ~ 1/*ɛ*) and substantial phonon-drag Seebeck components. Notably, because the partial Seebeck coefficients have opposite signs, the coexistence of electrons and holes actually amplifies the total Nernst thermopower (*S*_xy_), acting as the crucial source of large *S*_xy_ in topological semimetals. In low-dimensional systems, the Mott relation shows that a strong energy dependence of conductivity markedly enhances the Seebeck effect. The partial Seebeck coefficients, defined by the energy derivative of conductivity, become maximized under quasi-1D conditions owing to nearly δ-shaped energy-dependent density of states^2,4,5^. To further enhance the Nernst thermopower, we turn our focus to the mobility term (*μ*_yy_), which is underexplored in 3D semimetals. Low-dimensional semimetals exhibit pronounced mobility anisotropy; consequently the ambipolar contribution can be reformulated as (Supplementary material, Note I):

2 $${{{{\rm{S}}}}_{{xy}}}^{{{{\rm{ambipolar}}}}}=1/2\left({{S}_{{{{{\rm{yy}}}}}}}^{{{\rm{h}}}}-{{S}_{{{{{\rm{yy}}}}}}}^{{{\rm{e}}}}\right){B}_{z}{\mu }_{H}^{2}/{\mu }_{{{{\rm{xx}}}}}$$

where the band mobility *μ*_yy_ is reformulated by a reduced mobility$\tilde{\mu }$ = *μ*_H_^2^/*μ*_xx_, with *μ*_H_ the Hall mobility. As the dimensionality is reduced, carrier mobility becomes increasingly anisotropic, remaining high along the conducting direction while being strongly suppressed in the transverse directions. This pronounced transport anisotropy leads to a substantial enhancement of the reduced mobility.

In three-dimensional (3D) semimetals, the strongest Nernst response typically emerges within a narrow temperature window just below the onset of the phonon-phonon Umklapp process. In this region, the coexistence of high carrier mobility and phonon drag-enhanced partial Seebeck coefficients can yield Nernst thermopower on the order of millivolts per Kelvin^17–20^. Beyond this range, electron-phonon interactions, which are strongly constrained by the Debye temperature, further suppress any enhancement of the Nernst thermopower. By contrast, quasi-1D systems, owing to their sharp density of states, inherently sustain large partial Seebeck coefficients that scale with temperature^4^. When coupled to the phonon drag contributions, this results in enhanced Seebeck responses persisting over a broader temperature range. Furthermore, dimensional reduction amplifies both the effective-mass anisotropy with inter-chain effective masses approaching infinity, which may markedly enhance the reduced mobility $\tilde{\mu }$ (see Fig. 1a, b). Collectively, these effects yield a substantially amplified Nernst thermopower in quasi-1D LMO, with enhancements exceeding 160-fold compared with 3D counterparts (see Fig. 1d), and sustain large responses over extended temperature ranges, surpassing 8 mV/K from 20 to 41 K and 4 mV/K from 10–61 K (see Fig. 3h).

To quantitatively evaluate the role of reduced dimensionality, we employ a tight-binding model described by *H*_*±*_ = *c*_0_ -*t*_x_cos*k*_x_ - *t*_y_cos *k*_y_ - *t*_z_cos *k*_z_ ± Δ, where *t*_i_ represents hopping parameters, Δ controls the energy separation between the electron and hole bands, and *c*_0_ adjusts the band filling and carrier compensation. Because the multiband Nernst response is highly sensitive to both the difference between the electron and hole Seebeck coefficients and the degree of carrier compensation, we impose perfect compensation between the two bands with identical carrier fillings for all anisotropy ratios (Supplementary Fig. 1c, d). This construction eliminates variations arising from carrier imbalance, allowing the effect of dimensionality-induced Fermi-surface reconstruction to be isolated. We first fix *t*_y_ and set *t*_x_ = *t*_z_ = *K*_t_*t*_y_ with *K*_t_ varying from 1 to 0 to model the dimensional crossover from 3D to 1D. For each *K*_t_, the Fermi energy is determined self-consistently at fixed carrier filling, and the Nernst thermopower is calculated by solving the Boltzmann transport equation. As shown in Fig. 1c, the Nernst response increases monotonically with decreasing *K*_t_, demonstrating that the evolution toward a more anisotropic Fermi surface geometry can substantially amplify the Nernst response. To further distinguish the contribution of transport anisotropy from that of the Seebeck coefficient, we introduce an additional anisotropy by setting *t*_x_ = 2*t*_z_. For the same longitudinal Seebeck coefficient *S*_yy_, *S*_zy_ exhibits a much stronger enhancement than *S*_xy_, indicating that the enhanced Nernst response cannot be attributed solely to an increased Seebeck coefficient. Instead, the anisotropic Fermi-surface geometry enhances the Nernst response through the reduced mobility. Within this framework, the Nernst thermopower is governed by the dimensionality-dependent parameters *S*_xx_^h^ – *S*_xx_^e^ and *K*_m_. Reduced dimensionality, therefore, enhances the transverse thermoelectric response through two synergistic mechanisms: increasing the partial Seebeck coefficient and strengthening transport anisotropy, thereby providing additional degrees of freedom for boosting the Nernst response.

### Quasi-one-dimensional LMO with half-filled band and sharp quasi-linear dispersion

**Fig. 2:**
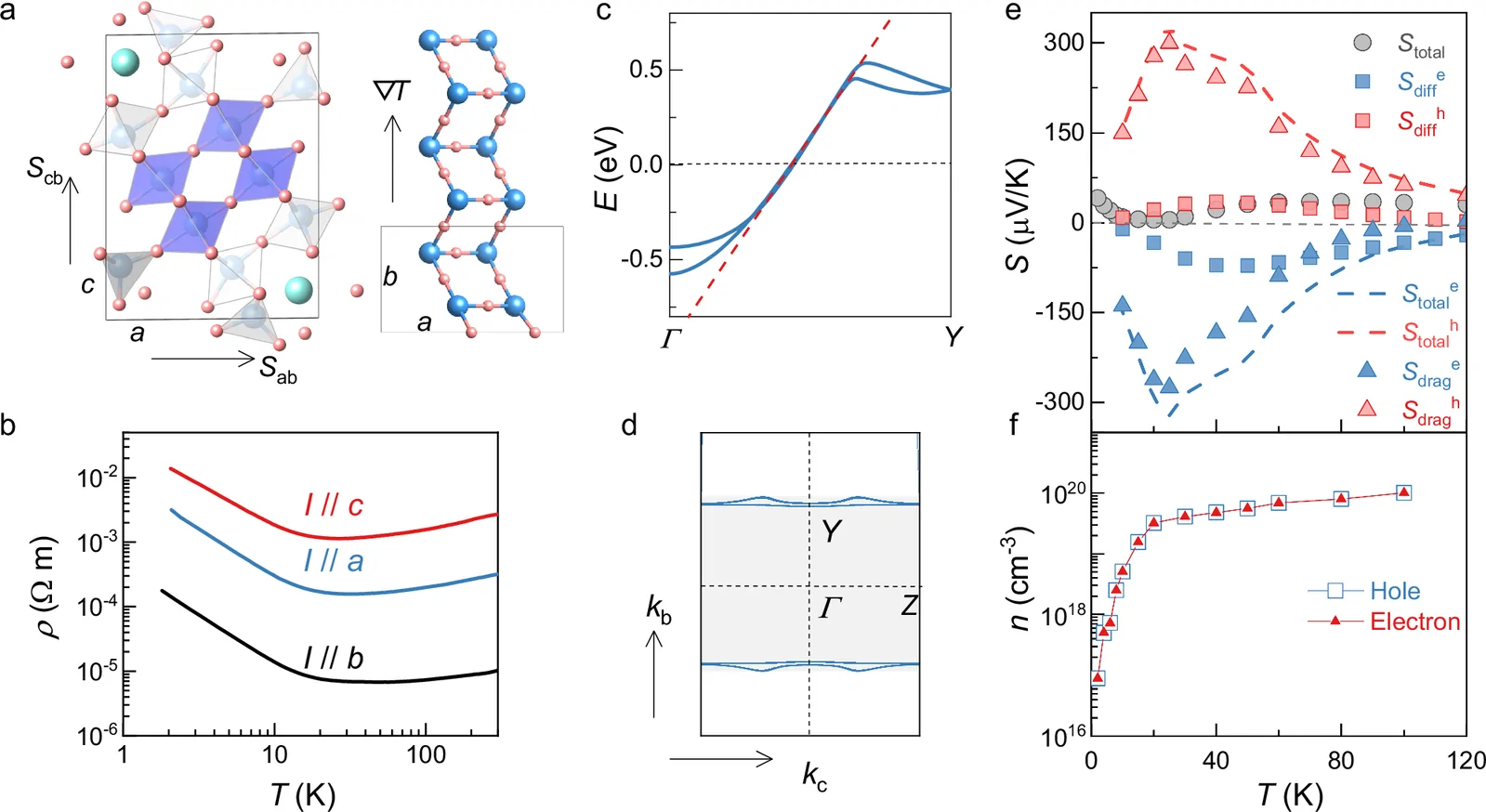
Crystal structure, electronic structure, and quasi-1D transport properties of LMO. **a** Crystal structure of LMO (emerald green, Li; steel blue, Mo; rose brown, O) together with the Nernst measurement configuration, where the heat current is applied along the conducting chains and the transverse voltage is detected along the inter-chain *a*-axis (*S*_ab_) and *c*-axis (*S*_cb_), respectively. **b** Axis-dependent resistivities. **c** Band structure along Γ-Y, showing a sharp quasi-linear band in the vicinity of the Fermi level *E*_F_ (red dashed line). **d** Quasi-1D Fermi surface of LMO within the Brillouin zone, comprising four nearly flat sheets. Both positive and negative curvature of sheets yield near-perfect compensation between electrons and holes. **e** The temperature dependence of Seebeck coefficients. A remarkably small net Seebeck coefficient (*S*_bb_ ~ 5 μV/K) is observed at 25 K, indicative of nearly perfect electron-hole compensation. The total partial Seebeck coefficients, extracted from extended Nernst Eq. (2) calculations (see Note 5 of the Supplementary Material for details), peak at 25 K with pronounced contributions from both phonon drag and diffusion. The partial diffusion contributions are obtained through DFT calculations. The partial phonon-drag contributions are then obtained by subtracting the diffusion component from the total contribution. The diffusion term, strongly enhanced by reduced dimensionality, dominates above 70 K and extends the temperature window of large Nernst response. Even at 25 K, the diffusion contribution remains significant, reaching ~50 μV/K, owing to the quasi-1D properties. **f** Temperature-dependent carrier concentration extracted from two-carrier model fitting.

Based on the derivation above, low-dimensional systems with perfect compensation and high mobility can be decent transverse TE systems. The quasi-1D LMO serves as an ideal platform for demonstrating the role of dimensional reduction in enhancing transverse TE effects. It crystallizes in a monoclinic structure (space group of P21/m1; see Fig. 2a) with its primitive cell consisting of two stacks of four corner-sharing MoO_6_ octahedra and two MoO_4_ tetrahedra. The weak orbital overlap between the adjacent stacks within the *ac*-plane results in quasi-1D electronic transport characteristics, with a conductive zigzag chain along the crystallographic *b*-axis. Only two of the four MoO_6_ units participate in electric conduction, conferring strong quasi-1D properties to the system. This quasi-1D transport is clearly manifested in the temperature-dependent resistivity (see Fig. 2b), with the resistivity ratio along the three principal axes (*ρ*_bb_: *ρ*_aa_: *ρ*_cc_) reaching ~ 1: 30: 262 at 300 K.

Electronic band structure (DFT) calculations reveal that the Fermi surface of LMO consists primarily of two half-filled quasi-1D bands (see Fig. 2c). These generate four nearly flat Fermi surface sheets (see Fig. 2d), mirrored by inversion symmetry. Each sheet contains both positive and negative curvature^23^, yielding near-perfect compensation between electrons and holes. Experimental Hall conductivity fitting by the two-carrier model and temperature dependence of Seebeck coefficient further confirm this compensation across the measured temperature range (see Fig. 2e, f). Above 20 K, the carrier densities remain nearly constant. By contrast, a sharp decline in both carrier types occurs below 20 K, which potentially indicates the onset of possibly correlated effects associated with the metal-insulator transition in *ρ*(*T*)^24–26^. Moreover, the sharp band dispersions near the Fermi level are quasi-linear, resembling those of topological semimetals and enabling relatively high mobility. The Hall mobility reaches 0.3 m^2^ V^−1^ s^−1^ at 2 K (Supplementary Fig. 8c).

### Difference in giant Nernst and Ettingshausen signals after fixing heat and electric currents

**Fig. 3:**
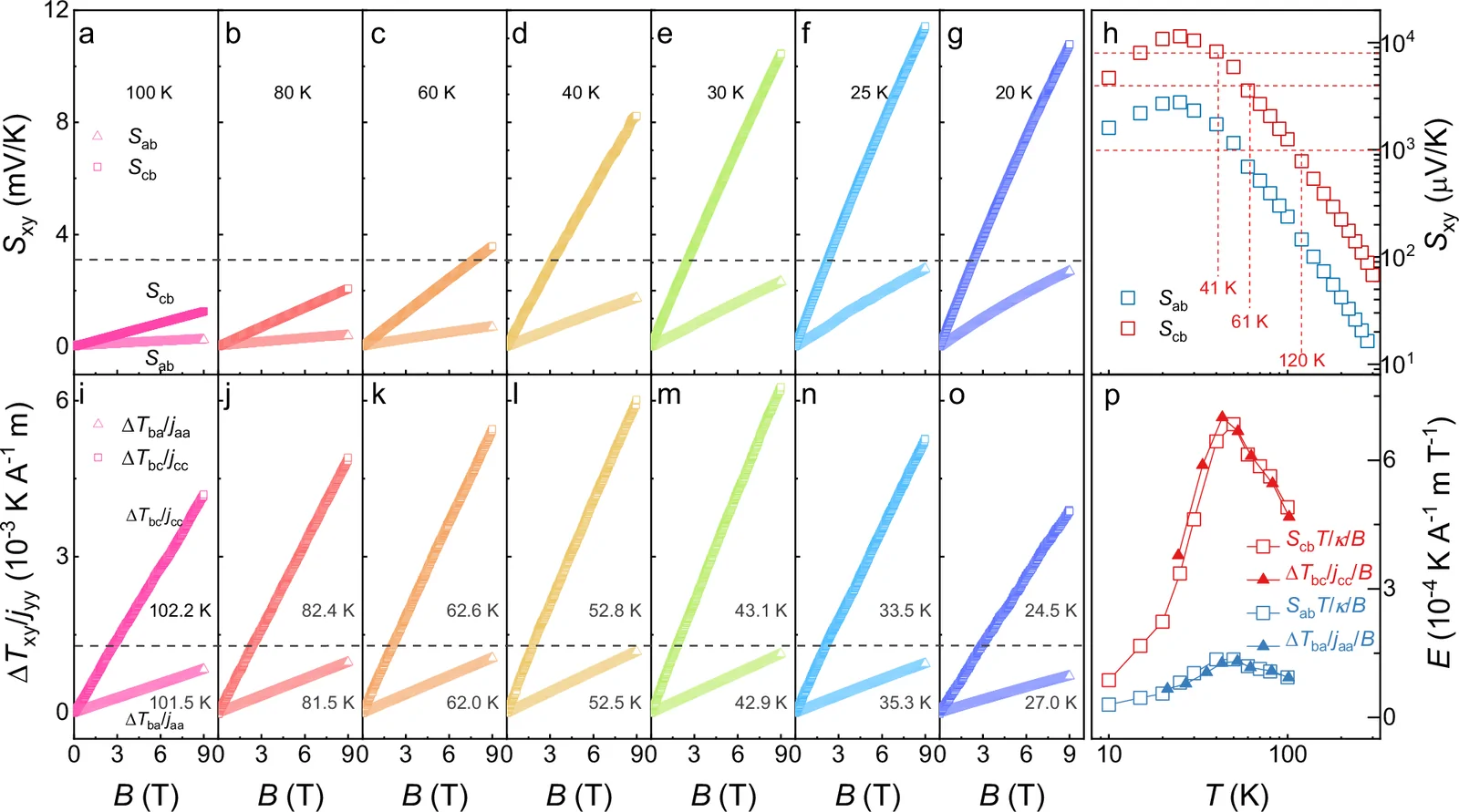
Dimensional reduction enhanced Nernst and Ettingshausen responses. **a–g** Magnetic field-dependent Nernst signals measured between 20 K and 100 K, with the heat current applied along the *b*-axis and the transverse voltage detected along the *a*-axis (*S*_ab_) and *c*-axis (*S*_cb_), respectively. **h** Temperature dependence of Nernst signals at 9 T with a significant difference between *S*_ab_ and *S*_cb_. **i–o** Magnetic-field-dependent Ettingshausen signals recorded between 24.5 K and 102.2 K, with the temperature difference measured along the *b*-axis and electric current applied along the *a*-axis (∆*T*_ba_/*j*_aa_) and *c*-axis (∆*T*_bc_/*j*_cc_), respectively. **p** Comparison of the Ettingshausen coefficient at 9 T obtained from the Bridgman relation with that determined by direct measurement.

The combination of carrier compensation and relatively high mobility in LMO provides a robust foundation for achieving large transverse TE signals. Moreover, the quasi-1D properties make it possible to verify the correlation between transverse geometry and transverse TE coefficients. In order to exclude the influence of Seebeck electric field (based on the extended Nernst Eq. (2)), we design the Nernst thermopower measurement under a fixed thermal gradient applied along the conductive *b*-axis (see Figs. 2a and 3a–g). The *S*_ab_ is proportional to the applied magnetic field and reached a maximum of 2760 μV/K at 25 K and 9 T. Remarkably, upon rotation of the transverse voltage probe towards a more quasi-1D orientation, *S*_cb_ exhibits a substantial enhancement, reaching a peak value of 11430 μV/K under identical measurement conditions. This value is second only to that of pure Bi^27,28^, yet occurs at a higher temperature (25 K vs 8 K), resulting in a substantial transverse TE signal across a broader temperature range (with values exceeding 1000 μV/K below 120 K, see Fig. 3h), highlighting the strong potential of LMO for transverse TE cooling applications.

To further examine the role of transverse geometry with the heat flow direction held constant, we measured the Ettingshausen signals, i.e., the magnetic field induced transverse temperature gradient with the temperature difference recorded along the *b*-axis (see Fig. 3i–o). The Ettingshausen response shows a similar field dependence to that of the Nernst thermopower, displaying an approximately linear increase with increasing field strength across 24.5–102.2 K range. Maximum Ettinghausen signals reach 1.18 × 10^−3^ Km/A and 6.3 × 10^−3^ Km/A at 52.5 K and 43.1 K for electric currents along the *a*- and *c*-axis, respectively, which are comparable to or even surpass that of Bi (0.45 × 10^−3^ Km/A at 6.8 K)^27,28^. While Bi exhibits extremely high mobility and conductivity at zero magnetic field, its conductivity is strongly suppressed under a magnetic field due to the Lorentz force, reaching the same order as that of LMO^27,28^. These results further underscore the Ettingshausen cooling capacity of LMO. Additionally, the Ettingshausen coefficient (*E*) and the Nernst thermopower (*S*_yx_) are related via the Bridgman relation, *E*_yx_ = *S*_xy_*T*/*κ*_yy_^29^, where *κ* denotes the thermal conductivity and is independent of magnetic field in LMO (Supplementary Fig. 3c). Figure 3p compares the Ettinghausen coefficients obtained via direct measurement with those calculated from the Nernst thermopower. The excellent agreement between the two provides compelling evidence for the pronounced difference in transverse TE response of LMO under fixed directions of heat flow and output temperature difference.

### The correlations between dimensional reduction and transverse TE signals

The giant Nernst thermopower and the pronounced disparity between *S*_ab_ and *S*_cb_ indicate that reduced dimensionality plays a crucial role in enhancing the transverse TE response. To elucidate its origin, we employ the extended Nernst Eq. (2). Similar to the case of topological semimetals, the carrier-diffusion contribution plays only a negligible role in enhancing the transverse TE response. Although the Hall angle is large, the Fermi energy is also comparatively high (*ɛ* ≈ 500 meV). Consequently, the enhancement associated with the large Hall angle is largely offset by the high Fermi energy, resulting in a carrier-diffusion contribution of only ~10 μV/K at 25 K. This value is nearly three orders of magnitude smaller than the measured Nernst thermopower of 11,470 μV/K. Therefore, the giant Nernst response is dominated by the ambipolar contribution (Supplementary Fig. 7b). Earlier studies have reported large *S*_ab_ and anisotropic transport properties focusing on the *ab* plane^30,31^, but overlooked the even larger *S*_cb_, attributing the enhancement to a phonon drag-induced Seebeck electric field within an isotropic Nernst framework. However, this simplified isotropic formulation fails to capture the giant difference between *S*_ab_ and *S*_cb_ when the direction of heat flow is fixed. In our results, the dimensional reduction and phonon drag together yield a large partial Seebeck electric field that persists over a wide temperature range (see Figs. 2e and 3h). At low temperatures, the phonon-drag Seebeck component dominates, particularly near the phonon peak. As the temperature increases, the contribution from the Seebeck effect induced by the low-dimensional density of states gradually becomes more significant (*S*_bb_^drag^ ≈ *S*_bb_^diffusion^ at 120 K; Supplementary Fig. 8f). Moreover, quasi-1D transport anisotropy further enhance Nernst thermopower via a extended Nernst Eq. (2) (Supplementary Fig. 8e), in which the reduced mobility$\tilde{\mu }$ embodies both intrinsic contributions from Fermi surface curvature *K*_m_.

**Fig. 4:**
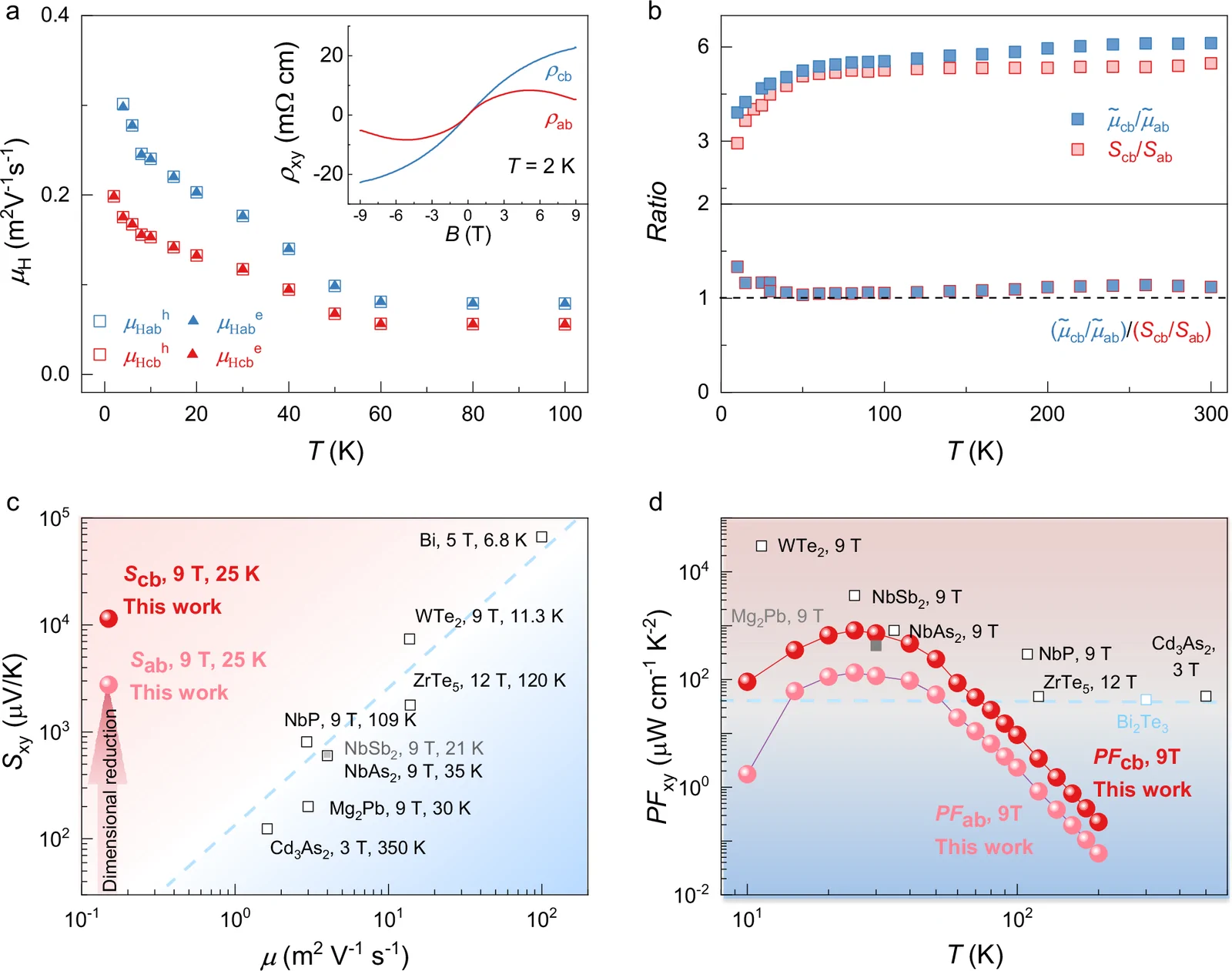
Hall mobility and transverse TE performance. **a** Temperature-dependent Hall mobility extracted from two-carrier model fitting. The inset shows the Hall resistivity measured at 2 K with the electric current applied along the *b*-axis and the transverse voltage detected along the *a*-axis (*ρ*_ab_) and *c*-axis (*ρ*_cb_), respectively. **b** Comparison between the ratios *S*_cb_/*S*_ab_ and ${\tilde{\mu }}_{{{{\rm{cb}}}}}/{\tilde{\mu }}_{{{{\rm{ab}}}}}$ as a function of temperature at 9 T with ${\widetilde{\mu }}_{{{\mathrm{cb}}}}$ *= μ*_Hcb_*²*/*μ*_cc_ and ${\tilde{\mu }}_{{{{\rm{ab}}}}}$ = *μ*_Hab_*²*/*μ*_aa_. In this analysis, differences in charge carriers between the *a*-axis and *c*-axis were neglected, with *ρ*_cc_/*ρ*_aa_ used as a proxy for *μ*_aa_/*μ*_c**c**_**. c** Comparison of the Nernst response and carrier mobility in LMO with those of representative transverse TE materials^8–20,27,28^. **d** Temperature-dependent transverse power factors of LMO benchmarked against other typical transverse TE systems and Bi_2_Te_3_^8–20,34^.

Figure 4a presents the temperature dependence of the Hall mobility. Both *μ*_Hab_ and *μ*_Hcb_ increase upon cooling, with *μ*_Hab_ consistently exceeding *μ*_Hcb_, reflecting the lower value of *μ*_cc_ compared to *μ*_aa_. We applied the particular subscription of *a* and *b* to represent the effective mass ratio *m*_aa_^*^/*m*_bb_^*^. Hall resistivity measurements at 2 K (see inset, Fig. 4a) further reveal a pronounced difference between *ρ*_cb_ and *ρ*_ab_, accompanied by nonlinear field dependence arising from multiband transport. To quantitatively assess the effect of dimensional reduction (manifested as the reduced mobility $\tilde{\mu }$) on Nernst signals, we compare the experimental ratio *S*_cb_/*S*_ab_ with the theoretical expression ${\tilde{\mu }}_{{{{\rm{cb}}}}}/{\tilde{\mu }}_{{{{\rm{ab}}}}}$ (Fig. 4b). The near identical temperature dependence and magnitude of these ratios provide strong evidence that the difference in Nernst response originates from the substantial difference in the reduced mobility induced by quasi-1D properties of LMO. The pronounced anisotropy in Fermi-surface geometry, characteristic of low-dimensional transport, thus governs the observed amplification of the transverse TE response.

Crucially, these findings broaden the conventional framework for understanding large transverse TE effects. In addition to the established roles of high mobility and large compensated Seebeck coefficients, we identify reduced dimensionality as a decisive factor that can dramatically amplify transverse TE responses. At 25 K, the reduced mobilities (${\tilde{\mu }}_{{{{\rm{ab}}}}}$ and ${\tilde{\mu }}_{{{{\rm{cb}}}}}$) reach 4.1 and 19.1 m^2^ V^−1^ s^−1^, corresponding to 22- and 160-fold enhancements in transverse TE signals compared with those expected from the bare Hall mobility and Seebeck electric fields (see Fig. 1d). Figure 4c benchmarks these mobility-dependent Nernst thermopower against other representative transverse TE materials. Despite its relatively modest Hall mobility, LMO yields a large Nernst signal second only to that of Bi, underscoring the critical role of dimensional reduction in enabling superior transverse TE responses.

### Giant transverse TE power factors below the liquid nitrogen temperature region

From an application perspective, the orthogonal configuration of heat flow and voltage output in transverse TEs not only simplifies device architecture but also enables high power density. Beyond this structural advantage, our results reveal that such an orthogonal geometry intrinsically provides a pathway to enhance both the transverse response and overall TE performance. Achieving a substantial transverse TE signal requires the heat-flux direction to sustain high carrier mobility and a large compensated Seebeck electric field, while the voltage-output direction maintains comparatively lower mobility. This yields a large reduced mobility, naturally realized in quasi-1D systems with anisotropic Fermi-surface geometry. This requirement directly challenges the conventional view that high resistivity suppresses the TE power factor. Instead, we demonstrate that dimensional reduction amplifies the transverse TE response by simultaneously enhancing the Seebeck electric field and the reduced mobility, thereby boosting the transverse power factor (*PF*_cb_ = *S*_cb_^2^/*ρ*_cc_ ~ *μ*_Hcb_^4^*ρ*_cc_) through geometric effects. As illustrated in Fig. 4d, the temperature-dependent power factor *PF*_ab_ reaches a maximum of ~139 μW cm^−1^ K^−2^ at 25 K and 9 T. The transverse TE response is further amplified along stronger quasi-one-dimensional direction, yielding a sixfold increase in power factor. Specifically, *PF*_cb_ attains 814 μW cm^−1^ K^−2^ at the same temperature and field, comparable to those of other reported transverse TE materials, including WTe_2_ and NbSb_2_. Notably, this high performance persists throughout the entire liquid nitrogen temperature range. At sub-liquid nitrogen temperature, the transverse power factor of LMO significantly exceeds that of Bi_2_Te_3_, the benchmark longitudinal TE material in commercial applications. This comparison underscores the substantial promise of LMO for high-efficiency transverse TE cooling in cryogenic environments.

## Discussion

In summary, we demonstrate that dimensional reduction provides an effective and previously under-explored strategy for enhancing transverse TE response and performance. Through a combination of theoretical modeling and experimental validation, we reveal that the record Nernst signal (11,430 μV/K at 25 K and 9 T) and Ettingshausen signal (0.0063 Km/A at 43.1 K and 9 T) observed in LMO originate not only from carrier compensation and high-mobility quasi-linear band dispersion, but more fundamentally from the emergence of exotic density-of-states distributions and pronounced Fermi-surface anisotropy induced by dimensional reduction. These contributions are quantitatively captured by an extended Nernst framework (Eq. (2)) that couples the reduced mobility with enhanced Seebeck electric fields, offering a new foundation for understanding and optimizing transverse TE transport. Moreover, the interplay between phonon drag and dimensional-reduction-driven modifications of the density of states gives rise to a large Seebeck electric field sustained over a wide temperature range, overcoming the long-standing limitation that substantial transverse TE signals are restricted to narrow temperature windows. Consequently, LMO exhibits a remarkably large transverse power factor across a broad temperature interval, with a peak value of 814 μW cm^−1^ K^−2^, while retaining excellent performance well below liquid-nitrogen temperatures. Its performance far exceeds that of most known transverse and even state-of-the-art longitudinal TE materials, underscoring its strong potential for low-temperature solid-state cooling. This work not only advances the fundamental understanding of low-dimensional transverse TE transport but also establishes a pathway towards the rational design of high-performance transverse TE materials for cryogenic applications.

Looking forward, the concept of reduced dimensionality opens multiple avenues for further advancing transverse thermoelectricity. A key challenge is achieving an optimal balance between an enhanced transverse response and sufficient electrical conductivity, particularly in transverse geometries where low mobility also limits charge transport. Beyond quasi-one-dimensional systems, future efforts may focus on low-dimensional topological semimetals, in which high reduced mobility and large partial Seebeck coefficients coexist. Such platforms offer a promising route to simultaneously amplify Nernst and Ettingshausen effects while suppressing excessive magnetoresistance, thereby enabling more practical and efficient cryogenic cooling devices. More broadly, integrating dimensional engineering with band topology may establish a general design paradigm for next-generation high-performance transverse TE materials.

## Methods

Single crystals of Li_0.9_Mo_6_O_17_ were synthesized via a temperature-gradient flux method^32^, using Li_2_MoO_4_, MoO_2_, and MoO_3_ powders as starting materials in a molar ratio of 3:3:14. The thoroughly milled precursors were sealed in a 12 cm evacuated quartz tube, pre-reacted at 58 °C for four days, and subsequently subjected to a temperature gradient of 12.5 °C/cm from 640 °C to 520 °C for two weeks. The ampoule was then cooled to room temperature at a rate of 100 °C per hour. The resulting crystals typically exhibited dimensions of approximately 4 × 3 × 1 mm^3^.

The first-principle calculations were carried out based on Density functional theory implemented in Vienna ab initio Simulation Package (VASP) with the projector augmented wave (PAW) method. The exchange-correlation energy functional was treated using the Perdew-Burke-Ernzerhof (PBE) scheme within the generalized gradient approximation (GGA). The plane-wave energy cutoff of 500 eV was adopted. The energy and force convergence criteria were set to 10^−7^ eV and 10^−5^ eV Å^−1^, respectively. The Brillouin zone was sampled with the Gamma-centered k-mesh of 5 × 8 × 4.

Electrical transport measurements, including resistivity and Hall resistivity, were carried out using a standard four-probe configuration on a Physical Property Measurement System (PPMS-9T, Quantum Design). Thermal/thermoelectric transport parameters were measured under high vacuum using a one-heater, two-(differential-)thermocouple steady-state method implemented within the same PPMS platform. To ensure uniform heat flow through the sample, thin copper sheets were attached at both ends of the sample. A strain gauge heater (~350 Ω) was then mounted on the hot end, while the cold end was fixed to a copper block with a piece of cigarette paper serving as electrical insulation. The differential thermocouple was fixed to the sample using black glue (Stycast 2850 FT). The Seebeck and Nernst voltages were measured by attaching two pairs of platinum wires to the sample with silver paste (Supplementary Fig. 12). During magnetic field sweeps at each temperature, measurements were conducted from 9 T to -9 T and back to 9 T. To eliminate self-inductance effect, the signals were symmetrized as ∆*T*(*B*) = ∆*T*(-*B*) + ∆*T*( + *B*); ∆*V*(*B*) = ∆*V*(-*B*) + ∆*V*( + *B*). For Ettingshausen temperature difference measurements, the same configuration was employed, with an electric current (25 mA) applied along the *a*- and *c*-axes, the output temperature difference measured along the *b*-axis, the hot end suspended, and the cold end fixed to the copper block.

## Supplementary information

Supplementary Information

Transparent Peer Review file

## Source data

Source Data

## Data Availability

The data generated in this study are provided in the Source Data file. Source data are provided with this paper.
