# Supplementary material for: Observation of the amplified transverse thermoelectric signals by reduced-dimensionality transport anisotropy
Source: Nat Commun. 2026 Sep 8;17:9607. doi: 10.1038/s41467-026-77569-y (PMC13554294; doi:10.1038/s41467-026-77569-y)
Supplement: Supplementary file 1 [file 41467_2026_77569_MOESM1_ESM.pdf]

Supplementary Information for

Observation of the amplified transverse thermoelectric signals by reduced-dimensionality transport anisotropy

Honghui Wang[1,2,*], Bin He[1,3,4*], Xiaolong Feng[1,*], Haihua Hu[1], Ralf Koban[1], Walter Schnelle[1], Erjian Cheng[1], Yu Pan[2], Xiaoyuan Zhou[2*], Claudia Felser[1*]

1. Max Planck Institute for Chemical Physics of Solids, Dresden, 01187 Germany.
2. College of Physics and Center of Quantum Materials & Devices of Chongqing University, Chongqing 401331, P. R. China
3. Shenyang National Laboratory for Materials Science, Institute of Metal Research, Chinese Academy of Science, Shenyang 110016, China.
4. School of Materials Science and Engineering, University of Science and Technology of China, Shenyang 110016, China.

* Correspondence to Honghui.Wang@cpfs.mpg.de, Bin.He@cpfs.mpg.de, Xiaolong.Feng@cpfs.mpg.de, Xiaoyuan2013@cqu.edu.cn, Claudia.Felser@cpfs.mpg.de

Supplementary Note 1: Anisotropic two-carrier Nernst signals with perfectly compensated electron-hole carriers

According to linear response equation,

$$\vec{J} = \vec{\sigma} \cdot \vec{E} + \vec{\alpha} \cdot (-\nabla \vec{T}), \tag{s1}$$

Where J, σ, E, a and ∇T are electric current density, conductivity, electric field, thermoelectric conductivity and temperature gradient, respectively. When measuring the thermoelectric coefficients, the electric current is set to zero and equation s1 can be expressed as:

$$\begin{bmatrix} \sigma_{\mathrm{xx}} \sigma_{xy} \\ \sigma_{\mathrm{yx}} \sigma_{yy} \end{bmatrix} \begin{bmatrix} E_{\mathrm{x}} \\ E_{\mathrm{y}} \end{bmatrix} = \begin{bmatrix} \alpha_{\mathrm{xx}} \alpha_{xy} \\ \alpha_{\mathrm{yx}} \alpha_{yy} \end{bmatrix} \begin{bmatrix} \nabla T_{\mathrm{x}} \\ \nabla T_{\mathrm{y}} \end{bmatrix}. \tag{s2}$$

Thus, the longitudinal and transverse thermoelectric signals can be expressed as:

$$S_{\mathrm{xx}} = \frac{E_{\mathrm{x}}}{\nabla T_X} = \frac{\alpha_{\mathrm{xx}} \sigma_{yy} - \alpha_{yx} \sigma_{xy}}{\sigma_{xx} \sigma_{yy} - \sigma_{xy} \sigma_{yx}}, \tag{s3}$$

$$S_{\mathrm{yx}} = -\frac{E_{\mathrm{y}}}{\nabla T_X} = \frac{\alpha_{\mathrm{xx}} \sigma_{yx} - \alpha_{yx} \sigma_{xx}}{\sigma_{xx} \sigma_{yy} - \sigma_{xy} \sigma_{yx}}. \tag{s4}$$

Due to

$$\vec{\alpha} = \vec{\sigma} \cdot \vec{S}. \tag{s5}$$,

The thermoelectric conductivity components α_{xx} and α_{yx} can be expressed as:

$$\alpha_{\mathrm{xx}} = \sigma_{\mathrm{xx}} \mathrm{S}_{\mathrm{xx}} + \sigma_{\mathrm{xy}} \mathrm{S}_{\mathrm{yx}}, \tag{s6}$$

$$\alpha_{\mathrm{yx}} = \sigma_{\mathrm{yx}} \mathrm{S}_{\mathrm{xx}} + \sigma_{\mathrm{yy}} \mathrm{S}_{\mathrm{yx}}. \tag{s7}$$

For a two-carrier system consisting of electrons (subscript e) and holes (subscript h), the conductivity and thermoelectric conductivity are given by:[1]

$$\sigma_{\mathrm{xx}} = \sigma_{xx}^{e} + \sigma_{xx}^{h} = \frac{\mathrm{n}_{exx} e \mu_{exx}}{1 + \mu_{He}^2 B^2} + \frac{\mathrm{n}_{hxx} e \mu_{hxx}}{1 + \mu_{Hh}^2 B^2}, \tag{s8}$$

$$\sigma_{\mathrm{yx}} = \sigma_{yx}^{e} + \sigma_{yx}^{h} = \frac{\mathrm{n}_{\mathrm{hxx}} e \mu_{Hh}^2 B}{1 + \mu_{Hh}^2 B^2} - \frac{\mathrm{n}_{exx} e \mu_{He}^2 B}{1 + \mu_{He}^2 B^2}, \tag{s9}$$

$$\alpha_{\mathrm{xx}} = \sigma^{\mathrm{e}}_{\mathrm{xx}} \mathrm{S}^{\mathrm{e}}_{\mathrm{xx}} + \sigma^{\mathrm{h}}_{\mathrm{xx}} \mathrm{S}^{\mathrm{h}}_{\mathrm{xx}} - \sigma^{e}_{\mathrm{yx}} \mathrm{S}^{\mathrm{e}}_{\mathrm{yx}} - \sigma^{h}_{\mathrm{yx}} \mathrm{S}^{\mathrm{h}}_{\mathrm{yx}}, \tag{s10}$$

$$\alpha_{\mathrm{yx}} = \sigma^{\mathrm{e}}_{\mathrm{yx}} \mathrm{S}^{\mathrm{e}}_{\mathrm{xx}} + \sigma^{\mathrm{h}}_{\mathrm{yx}} \mathrm{S}^{\mathrm{h}}_{\mathrm{xx}} + \sigma^{e}_{\mathrm{yy}} \mathrm{S}^{\mathrm{e}}_{\mathrm{yx}} + \sigma^{h}_{\mathrm{yy}} \mathrm{S}^{\mathrm{h}}_{\mathrm{yx}}. \tag{s11}$$

Neglecting the second-order Hall term, the Nernst signals can be approximated as：

$$S_{\mathrm{yx}} \approx \frac{S_{yx}^{e} \sigma_{yy}^{e} + S_{yx}^{h} \sigma_{yy}^{h}}{\sigma_{yy}} + \frac{(\sigma_{xx}^{e} \sigma_{yx}^{h} - \sigma_{xx}^{h} \sigma_{yx}^{e})(S_{xx}^{h} - S_{xx}^{e})}{\sigma_{xx} \sigma_{yy}}. \tag{s12}$$

For a perfectly compensated two-carrier system ($n_e = n_h$, $\mu_e = \mu_h$), substituting s8 and s9 into s12, neglecting differences in charge carriers between the *x*-axis and *y*-axis, equation s12 can be rewritten as:

$$S_{\mathrm{yx}} = S_{\mathrm{diffusion}} + S_{\mathrm{ambipolar}} = \frac{S_{yx}^{e}\sigma_{yy}^{e} + S_{yx}^{h}\sigma_{yy}^{h}}{\sigma_{yy}} + 1/2\mu_{\mathrm{H}}^{2}/\mu_{\mathrm{yy}}B(S_{xx}^{h} - S_{xx}^{e}) \ . \qquad \text{(s13)}$$

The $\mu_{\mathrm{H}}^2/\mu_{\mathrm{yy}}$ is named as the reduced mobility. For isotropic systems ($\mu_{xx} = \mu_{yy} = \mu_H$), equation s13 can be rewritten as:

$$S_{\mathrm{ambipolar}} = 1/2\mu_{\mathrm{xx}}B(S_{xx}^{h} - S_{xx}^{e}) \, . \qquad \text{(s14)}$$

Supplementary Note 2: Anisotropic Nernst model

The thermoelectric transport is evaluated based on Boltzmann transport equation

$$\frac{\partial f}{\partial t}+\dot{r}\cdot\nabla_r f+\dot{k}\cdot\nabla_k f=\left(\frac{\partial f}{\partial t}\right)_{coll}, \tag{s15}$$

with $\dot{r}=v_{nk}$ and $\hbar\dot{k}=-e(E+v_{nk}\times B)$.

Under relaxation time approximation we have $\left(\frac{\partial f}{\partial t}\right)_{coll}=-\frac{f-f_0}{\tau_{nk}}$, with f_0 the equilibrium Fermi-Dirac distribution and τ_{nk} the relaxation time. Under weak electric field and temperature gradient, the distribution is linearized as $f=f_0+f_1$, with f_1 the first-order deviation from equilibrium. Under steady condition, $\frac{\partial f}{\partial t}=0$. Therefore, the Boltzmann equation is reduced to

$$v_{nk}\cdot\nabla_r f_0-eE\cdot v_{nk}\frac{\partial f_0}{\partial\varepsilon}-\frac{e}{\hbar}(v_{nk}\times B)\cdot\nabla_k f_1=-\frac{f_1}{\tau_{nk}}, \tag{s16}$$

since $\nabla_k f_0=\frac{\partial f_0}{\partial\varepsilon}\nabla_k\varepsilon=\hbar v_{nk}\frac{\partial f_0}{\partial\varepsilon}$. For $\nabla_r f_0=\frac{\partial f_0}{\partial T}\nabla T+\frac{\partial f_0}{\partial\mu}\nabla\mu=(-\frac{\partial f_0}{\partial\varepsilon})(\nabla\mu+\frac{\varepsilon-\mu}{T}\nabla T)$, the BTE can be further reduced to

$$[1+\tau_{nk}\Omega]f_1=\tau_{nk}v_{nk}\cdot F(-\frac{\partial f_0}{\partial\varepsilon}) \tag{s17}$$

Where $\Omega=-\frac{e}{\hbar}(v_{nk}\times B)\cdot\nabla_k$, and the generalized force $F=-\nabla\mu-\frac{\varepsilon-\mu}{T}\nabla T-\mathrm{eE}$. At weak magnetic fields, the distribution can be obtained as

$$\begin{aligned}
f_1&=[1+\tau\Omega]^{-1}\tau v_j\cdot F\left(-\frac{\partial f_0}{\partial\varepsilon}\right)\\
&\approx[1-\tau\Omega]\tau v_j\cdot F\left(-\frac{\partial f_0}{\partial\varepsilon}\right)\\
&=\tau F\left(-\frac{\partial f_0}{\partial\varepsilon}\right)\left[v_j-\tau\Omega v_j\right]\\
&=\tau F\left(-\frac{\partial f_0}{\partial\varepsilon}\right)\left[v_j+e\tau\epsilon_{lmn}v_l B_m\frac{\partial v_j}{\partial k_n}\right]
\end{aligned} \tag{s18}$$

assuming k-independent relaxation time $\tau_{nk}=\tau$. Consider $J=\sigma E+\alpha\nabla T+\ldots$, the electric and thermoelectric conductivity then can be obtained as

$$\sigma_{ij}=\frac{e^2}{V}\int\tau v_i(v_j+e\tau\epsilon_{lmn}v_l B_m\frac{\partial v_j}{\partial k_n})(-\frac{\partial f}{\partial\varepsilon})d\varepsilon \tag{s19}$$

$$\alpha_{ij}=\frac{e}{TV}\int\tau v_i(v_j+e\tau\epsilon_{lmn}v_l B_m\frac{\partial v_j}{\partial k_n})(\varepsilon-\mu)(-\frac{\partial f}{\partial\varepsilon})d\varepsilon. \tag{s20}$$

within constant relaxation time approximation $\tau_{nk}=\tau$.

The Seebeck and Nernst coefficient correspond to the diagonal and off-diagonal S. Here, we take the model $H_\pm = c_0 + t_x\cos k_x + t_y\cos k_y + t_z\cos k_z \pm \Delta$. t_y is set to 0.8 eV as the conduction direction, and $t_{x(z)}$ varies from 0.01 to 0.8 eV to investigate the anisotropic transport. To clarify the effect of anisotropy, the band is set to the same electron fillings via tuning c_0 and Δ. A constant relaxation time approximation (CRTA) with τ_0=80fs is applied. A orthogonal magnetic field B = 1 T is applied to evaluate the Nernst thermopower.

For Seebeck effect, only one metallic band was considered. As the system evolves from isotropic three-dimensional system to highly anisotropic system, the same electron fillings

denotes a lower Fermi energy and higher *DOS* in anisotropic system as shown in Supplementary Figs. 1a-b, ensuring the enhanced Seebeck effect in highly symmetric system.

Unlike the Seebeck effect, which is often discussed within single band, the Nernst effect arises from the contributions of electron and hole bands, with its magnitude determined by their respective Seebeck coefficients and compensation. To clarify the anisotropy effect on Nernst, we set the perfect compensation between electron and hole bands. Similar to the discussion of Seebeck effect, the fillings of electron and hole bands remains the same for different anisotropy, as shown in Supplementary Figs. 1c-d. As the anisotropy ratio increases, the system undergoes a Lifshitz transition from closed pockets to open sheets upon touching the boundary of the Brillouin zone. This transition substantially reshapes the Fermi surface, i.e., the distribution of charge carriers, rendering the notion of anisotropy less well-defined in this vicinity. This accounts for the sign change around $K_t \approx 0.5$, whereas the Nernst response grows monotonically with anisotropy on both sides of the transition.

In addition, further modifications are introduced to assess the role of relaxation time and in-plane anisotropy. (I) $\tau = \tau_0 g^{-1}(E)$. A *DOS*-dependent scattering model was evaluated, which presents negligible changes. This approximation may be relevant to short-range impurity scattering with weak momentum-dependence. However, this should be considered as a simplified phenomenological model rather than a universal scattering mechanism, since realistic scattering can depend on momentum, band index and microscopic defects. (II) $t_x = 2t_z$. An additional in-plane anisotropy was introduced, which further enhanced the Nernst effect. A clear in-plane anisotropy emerges with $S_{zy} > S_{xy}$ for $t_z < t_x$, reflecting the increased directional asymmetry.

Supplementary Note 3: Anistropic magnetoresistivity and Hall resisitivity

Magnetoresistivity and Hall resistivity are shown in Supplementary Figs. 4a–f. Based on the compensated band structure along the conducting chain (DFT calculations indicate that, in the metallic regime, the Fermi surface of LMO is primarily composed of two half-filled quasi-one-dimensional bands. These bands give rise to four nearly flat Fermi surface sheets (Fig. 2d). Each sheet contains regions of both positive and negative curvature, resulting in nearly perfect compensation between electrons and holes. However, due to possible correlation effects, the system undergoes a metal-insulator transition with the opening of a gap below ~25 K, while compensated electrons and holes may still persist.[3]), the temperature-dependent carrier concentrations (Fig. 2f) and Hall mobility (Fig. 4a) are extracted using a two-carrier model $\sigma_{yx} = eB(\frac{n_1\mu_1^2}{1+\mu_1^2B^2} - \frac{n_2\mu_2^2}{1+\mu_2^2B^2})$. In this analysis, the Hall conductivity can be approximated as $\sigma_{ab} \approx \rho_{ab}/\rho_a\rho_b$ and $\sigma_{cb} \approx \rho_{cb}/\rho_c\rho_b$, given that $\rho_a\rho_b >> \rho_{ab}\rho_{ba}$ and $\rho_c\rho_b >> \rho_{cb}\rho_{bc}$.

Supplementary Note 4: Hall angle

The Hall angles of electrons and holes is calculated by the formula $\tan\Theta = \sigma_{cb}^{e}/\sigma_{bb}$ and $\tan\Theta = \sigma_{cb}^{h}/\sigma_{bb}$ with $\sigma_{yx}^{h} = n_{h}e\mu_{Hh}^{2}B/(1+\mu_{Hh}^{2}B^{2})$ and $\sigma_{yx}^{e} = -n_{e}e\mu_{He}^{2}B/(1+\mu_{He}^{2}B^{2})$). Based on Mott relation and linear response equation, the diffusive Nernst contribution can be expressed as $S_{yx}^{Diffusion} = \pi^{2}/3(k_{B}^{2}T/e)\tan\Theta/\varepsilon$ for single band system. Furthermore, the diffusive Nernst contribution can be expressed as $S_{yx}^{Diffusion} = \pi^{2}/3(k_{B}^{2}T/e)(\tan\Theta^{h}-\tan\Theta^{e})/\varepsilon$ for two-band system. Although the Hall angle is relatively large, the Fermi energy is also comparatively high ($\varepsilon \approx 500$ meV). Consequently, the diffusive contribution is only ~10 μV/K at 25 K, which is three orders of magnitude smaller than the measured total Nernst thermopower (11470 μV/K). The ambipolar Nernst component is obtained by subtracting diffusive contribution from the total signal. These results indicate that the ambipolar contribution dominates the large Nernst signal.

Supplementary Note 5: Longitudinal thermopower

For LMO, the total partial Seebeck coefficients S_{total}^{h} and S_{total}^{e} (Supplementary Fig. 8a) are calculated based on the wannier tight-binding model, which are constructed based on the DFT calculation using wannier90 package,[4-5] with the Mo-*d* orbitals included. Supplementary Fig. 8b shows the temperature dependence of Seebeck coefficients measured along *b*- and *a*-axes. To obtain the total partial Seebeck coefficients, the reduced mobility is first calculated in Supplementary Fig. 8c and the difference between total partial Seebeck coefficients (S_{total}^{h} - S_{total}^{e}) is then determined using Eq. (2) in the main text. Combined with the temperature-dependent S_b ($S_b = S_{total}^{h} + S_{total}^{e}$; Supplementary Fig. 8b), the resulting total partial Seebeck coefficients are shown in Supplementary Fig. 8d. The phonon-drag partial Seebeck contributions are obtained by calculating the diffusion contributions from the total partial Seebeck coefficients. The Nernst signals arising from different contributions are further distinguished in Supplementary Figs. 8e-f. Supplementary Figure 8e demonstrates that, under quasi-one-dimensional conditions, the introduction of reduced mobility significantly enhances the Nernst signal compared to that based on the Hall mobility. Supplementary Figure 8f separates the phonon-drag and diffusive contributions to the Nernst signal. At low temperatures, the Nernst response is dominated by phonon drag, whereas with increasing temperature, the diffusive contribution gradually becomes more significant.

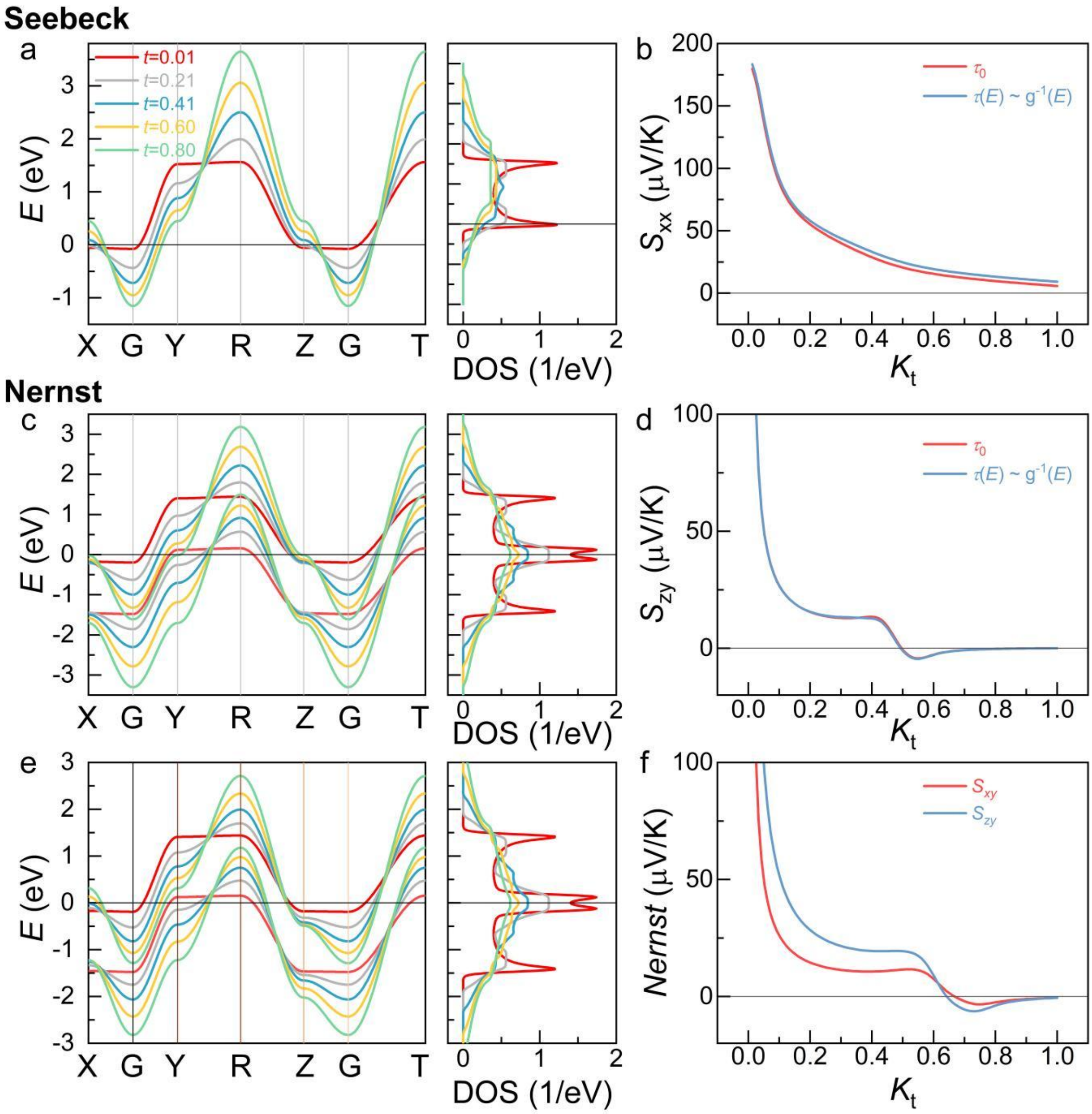

Supplementary Figure 1 | Anisotropic model. **a-b** Band structure, density of states for single band, and Seebeck response with varied anisotropy and same band filling at the Fermi level. **c-d** Band structure for compensated electron and hole bands, and corresponding Nernst reponse with $t_x=t_z$ for constant relaxation time and *DOS*-dependent relaxation time. **e-f** Band structure for compensated electron and hole bands, and corresponding Nernst reponse with $t_x=2t_z$.

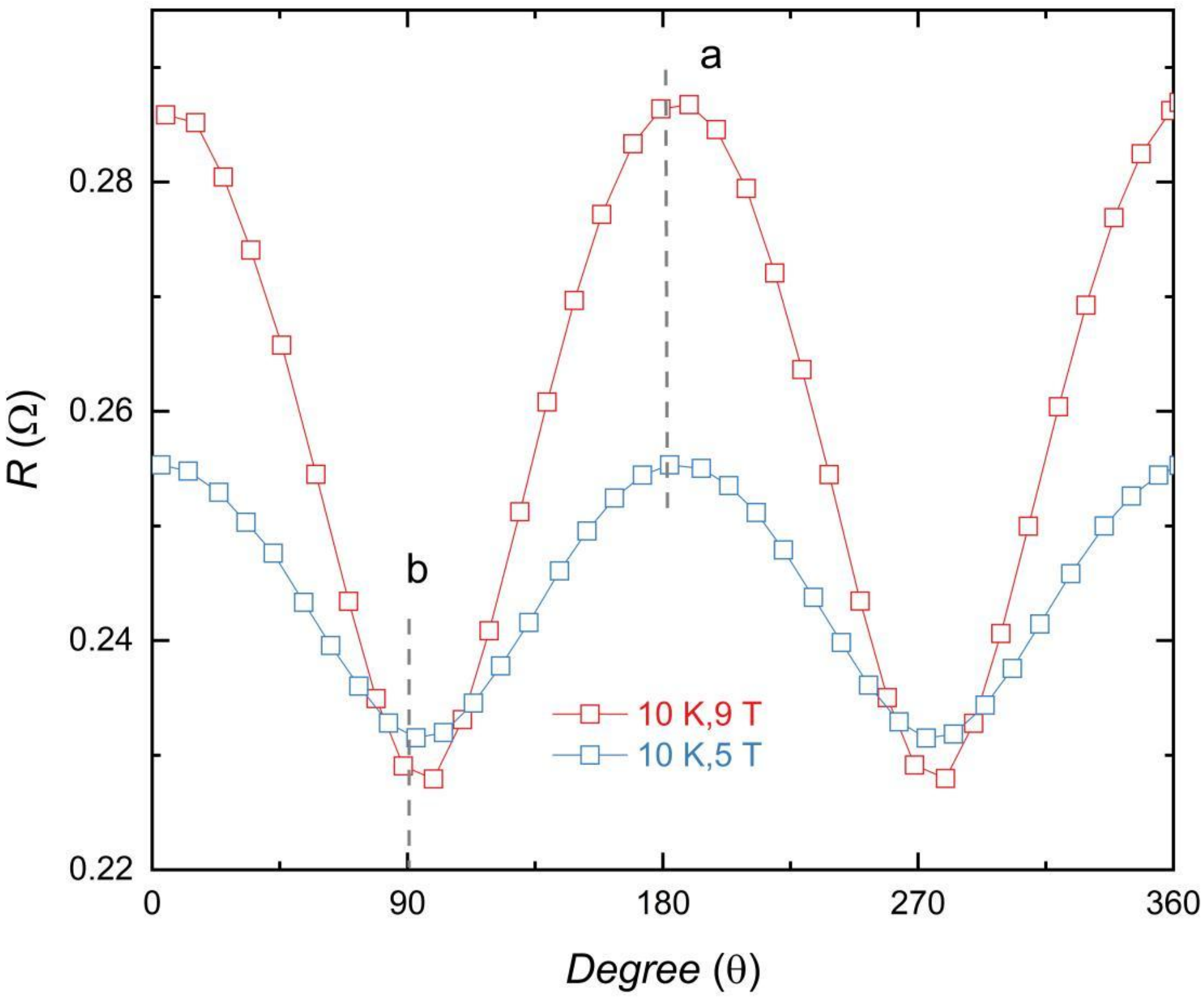

Supplementary Figure 2 | Rotating magnetoresistance. Angular-dependent the *ab*-plane magnetoresistance. Crystal orientation was determined through *MR* (θ).[2]

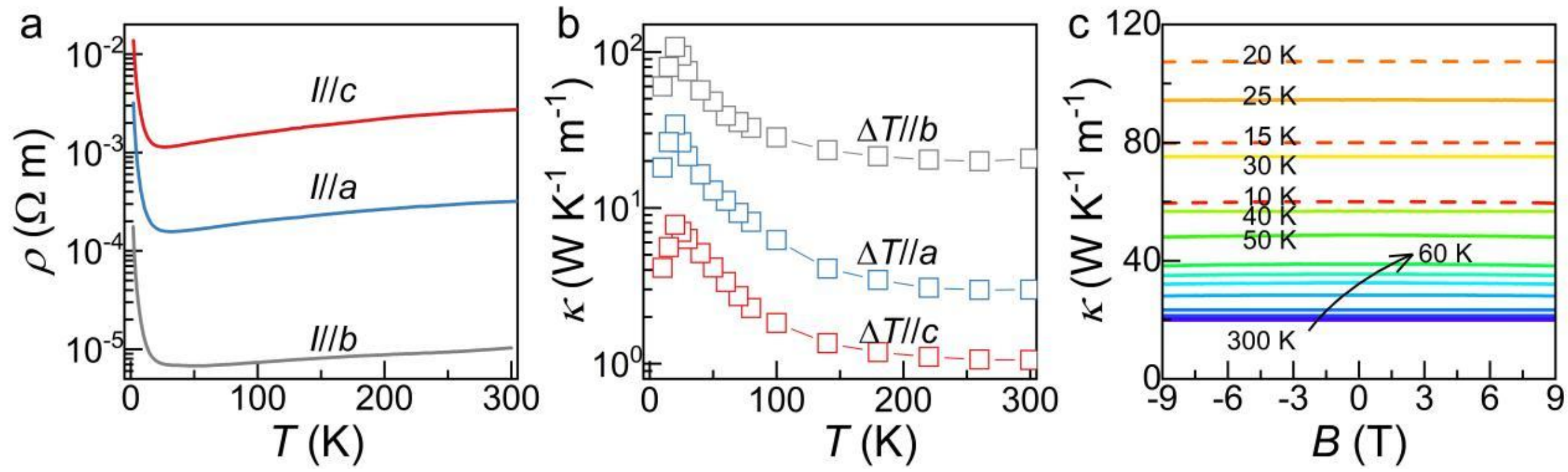

Supplementary Figure 3 | Resistivity, thermal conductivity and magneto-thermal conductivity. **a** Anisotropic electrical resistivity and **b** thermal conductivity measured with the electric current and heat current applied along the *b*-, *a*-, and *c*-axes, respectively. **c** Magnetic field dependence of the thermal conductivity along *b*-axis over the temperature range from 10 K to 300 K.

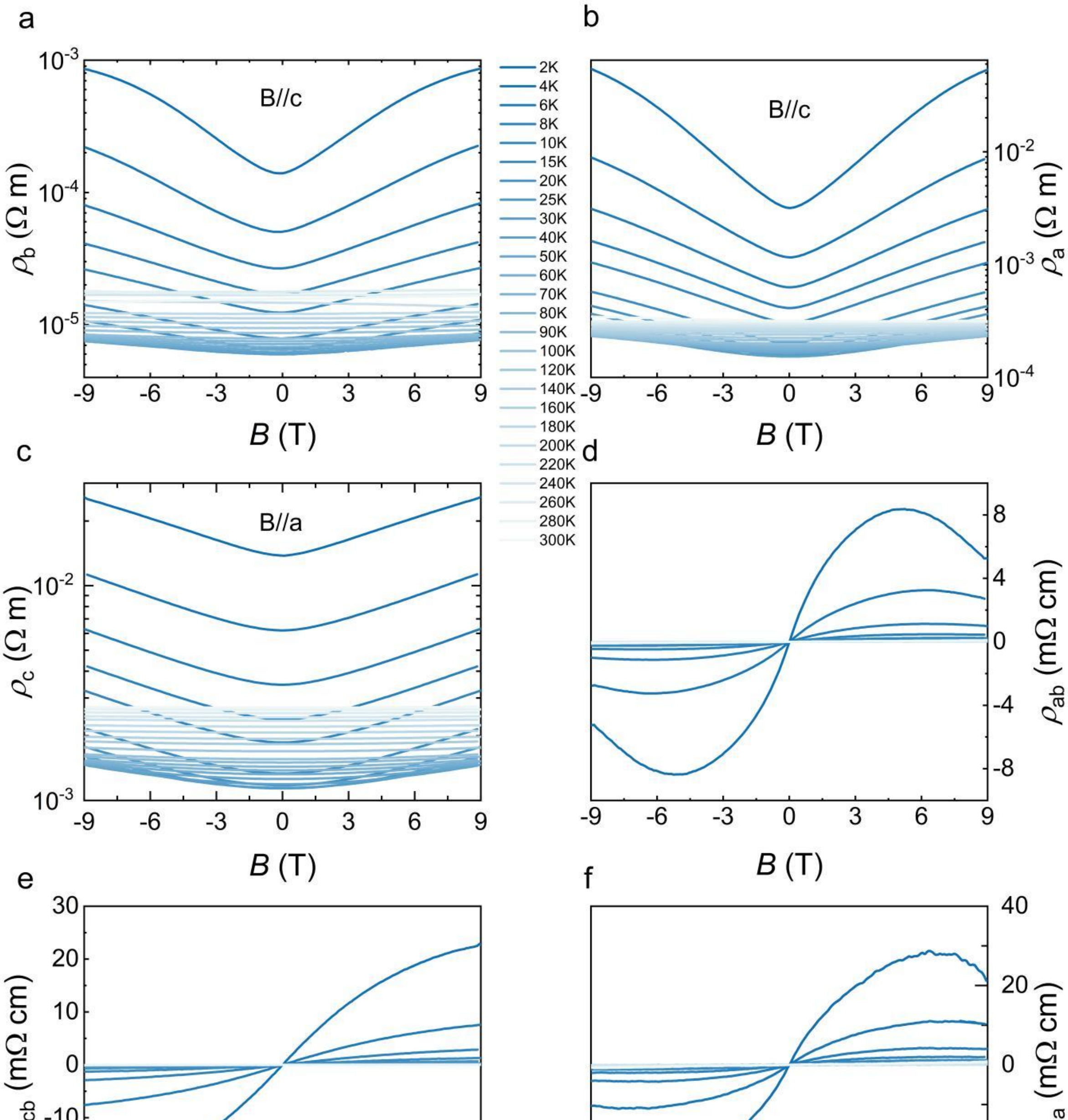

Supplementary Figure 4 | Magnetoresistivity and Hall. The magnetoresistivity with the electric current applied along *b*-axis **a**, *a*-axis **b** and *c*-axis **c**, respectively. **d** and **e** The Hall resistivity with the electric current applied along *b*-axis and magnetic field along *c*-axis and *a*-axis, respectively, in the temperature range from 2 K to 300 K. **f** The Hall resistivity with the electric current applied along *a*-axis and magnetic field along *c*-axis in the temperature range from 2 K to 300 K.

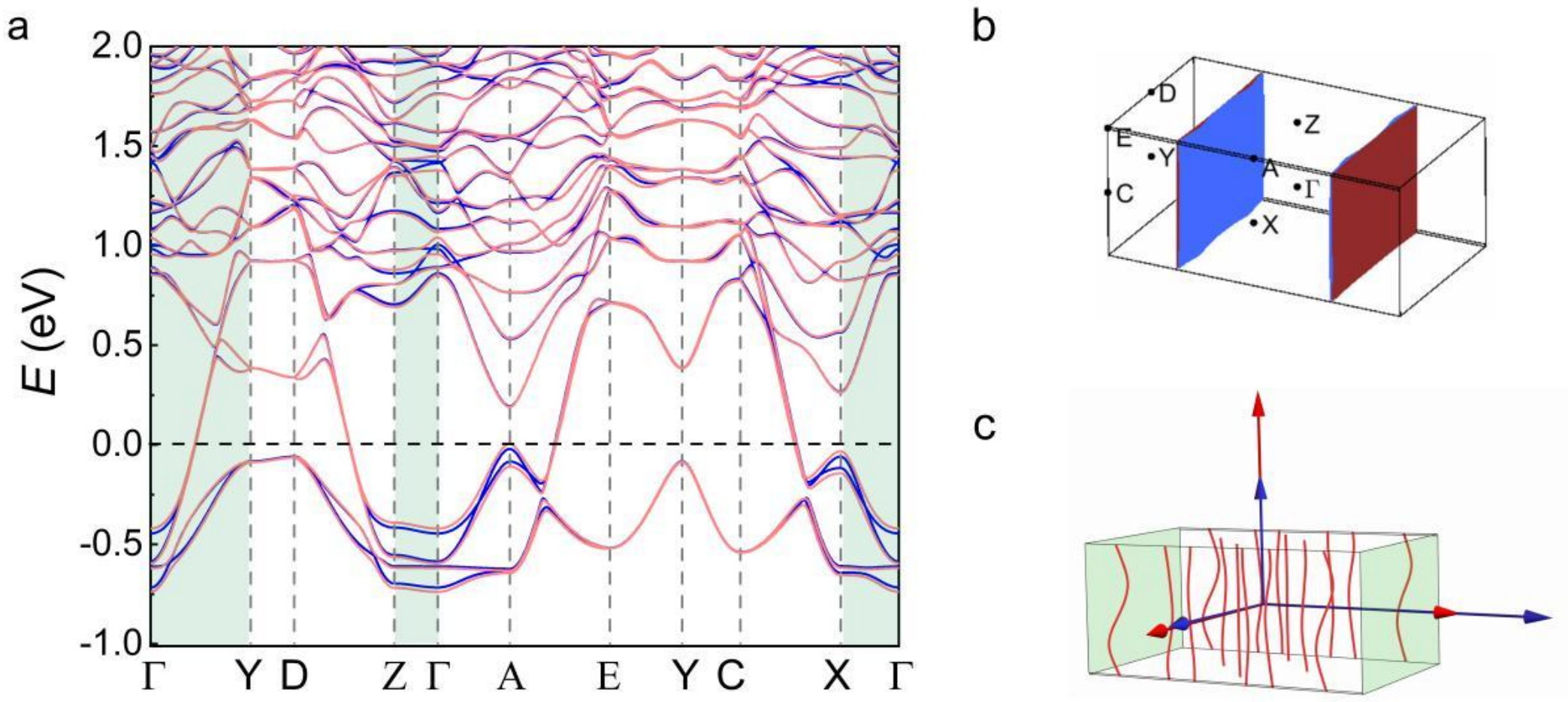

Supplementary Figure 5 | Band structure. **a** Band structure for LMO without (blue) and with (red) SOC, which shows few differences as the bands are dominated by Mo 4*d* orbitals. The first-principle calculations were carried out based on Density functional theory implemented in Vienna ab initio Simulation Package (VASP) with the projector augmented wave (PAW) method. The exchange-correlation energy functional was treated using the Perdew-Burke-Ernzerhof (PBE) scheme within the generalized gradient approximation (GGA). The plane-wave energy cutoff of 500 eV was adopted. The energy and force convergence criteria were set to 10^{-7} eV and 10^{-5} eV Å−1, respectively. The Brillouin zone was sampled with the Gamma-centred k- mesh of 5×8×4. The three colour blocks represent the bands along the *b*-axis (Γ-Y), the *a*-axis (Γ-X) and the *c*-axis (Γ-Z), respectively. Along the *b*-axis, the sharp bands cut through the Fermi level. However, the dispersion relation weakens along the *a*-axis and has even flattened out along the *c*-axis, which further indicates the quasi-one-dimensional nature of LMO. **b** Fermi surface for LMO, which is composed of two pairs of nearly flat Fermi surface sheets, confirming the 1D feature of LMO system. **c** Nodal degeneracy due to the crossing of two bands close to the Fermi level. The green shade areas denotes the nodal surface at $k_y = \pi$ plane, and the red lines denote the nodal lines which are gaped out with the inclusion of SOC.

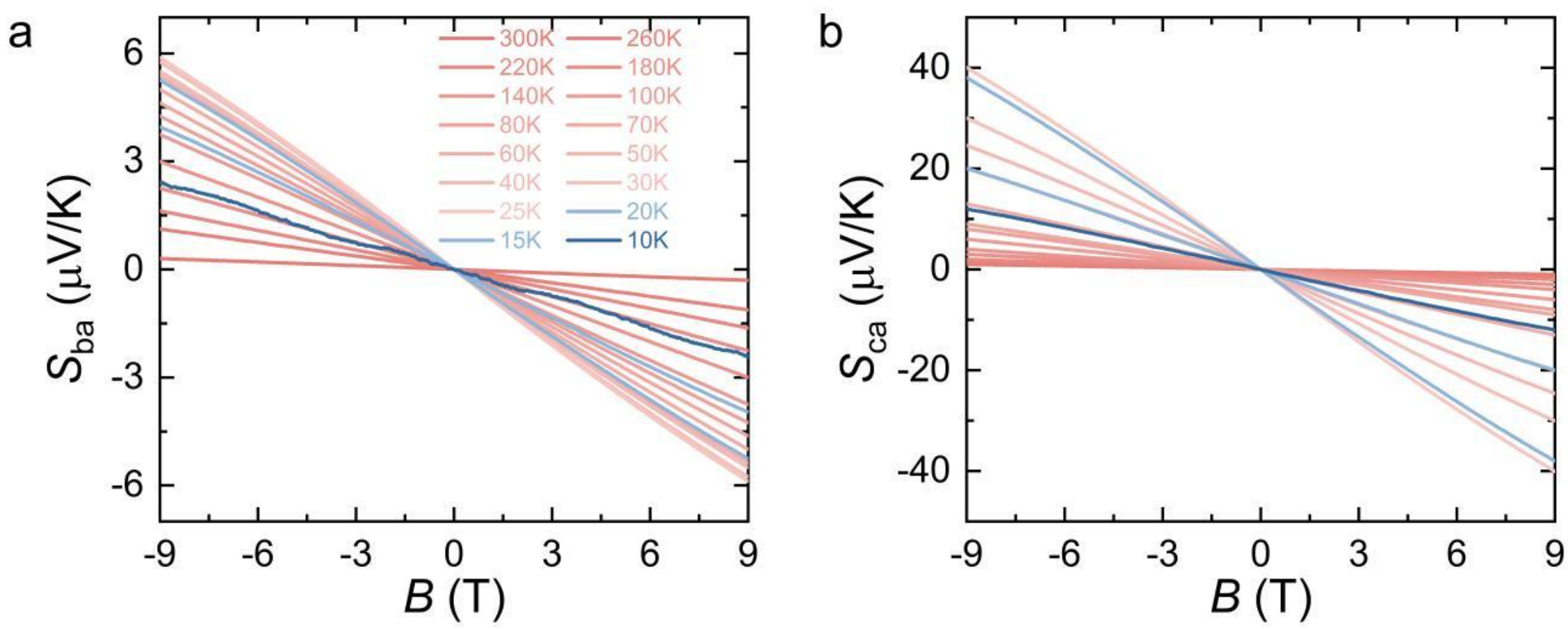

Supplementary Figure 6 | S_{ba} and S_{ca}. Magnetic field-dependent Nernst signals with the heat current applied along *a*-axis and the magnetic field along *c*-axis **a** and *b*-axis **b**, respectively.

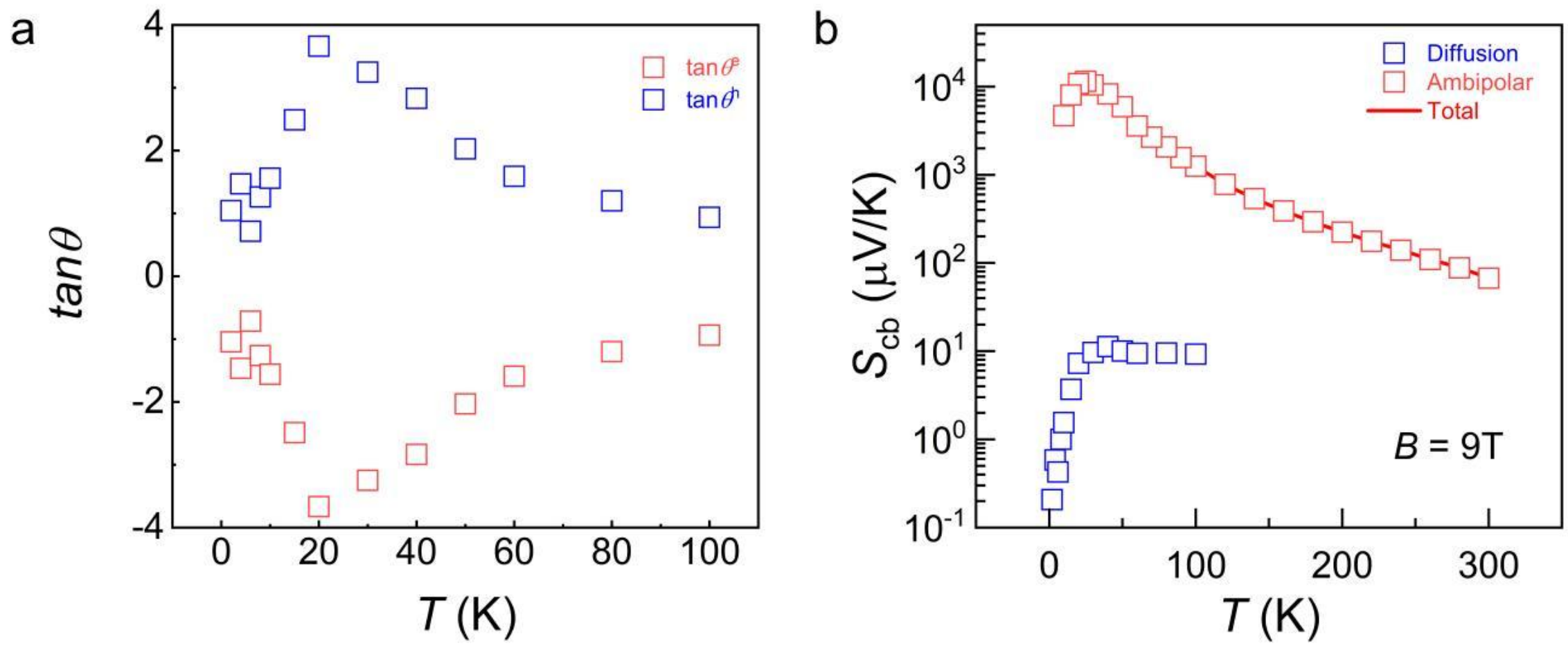

Supplementary Figure 7 | Diffusive contribution. **a** Temperature-dependent Hall angles of electrons and holes ($\sigma_{cb}^{e}/\sigma_{bb}$; $\sigma_{cb}^{h}/\sigma_{bb}$) from 2 K to 100 K under a magnetic field of 9 T. **b** The quantitative analysis of the diffusion and ambipolar Nernst contributions. The diffusive component is obtained by the formula $S_{yx}^{\mathrm{Diffusion}} = \pi^2/3(k_B^2 T/e)(\tan\Theta^h - \tan\Theta^e)/\varepsilon$. The ambipolar contribution is obtained by subtracting diffusion contribution from the total signal.

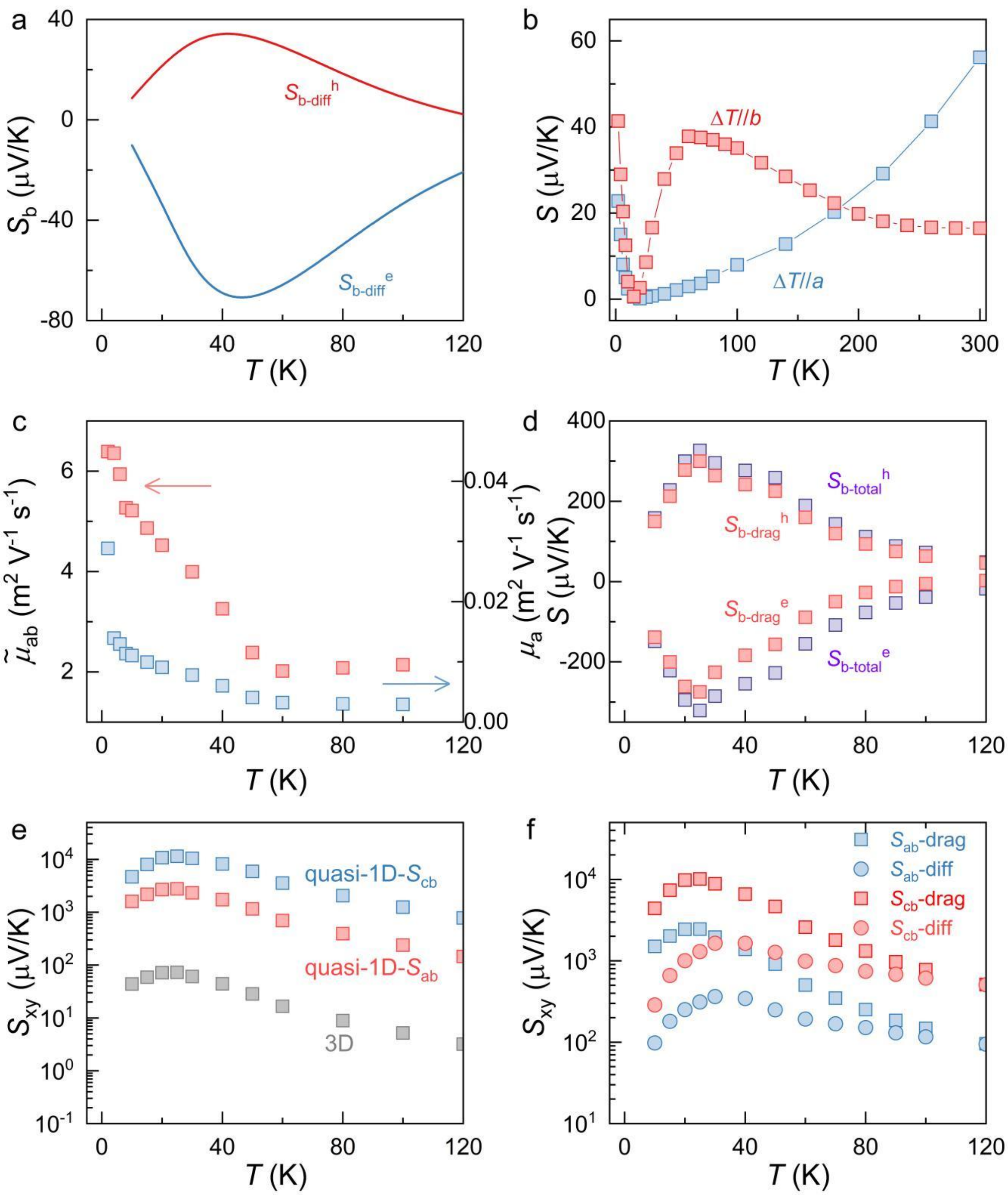

Supplementary Figure 8 | Enhancement of the Nernst signal by reduced dimensionality. **a** Temperature dependence of the partial diffusion Seebeck coefficient, obtained through DFT calculations. **b** Temperature dependent Seebeck coefficients with the heat current applied along *b*- and *a*-axes, respectively. **c** Temperature dependence of the carrier mobility μ_a and the reduced mobility $\tilde{\mu}_{ab}$ (μ_{Hab}^2/μ_a). The μ_a is obtained through low field fitting of Fig. S4f. The Hall mobility μ_{Hab} is obtained through two-carrier model fitting. **d** Temperature dependence of the total partial Seebeck coefficients and the corresponding phonon-drag contributions. **e** The temperature-dependent Nernst signals S_{ab} and S_{cb} for quasi-one-dimensional LMO, compared with that of a three-dimensional case, where only the Hall mobility is considered and the reduced mobility is neglected. **f** Temperature-dependent Nernst signals in S_{ab} and S_{cb}, highlighting the respective contributions from phonon drag and diffusive Seebeck effects.

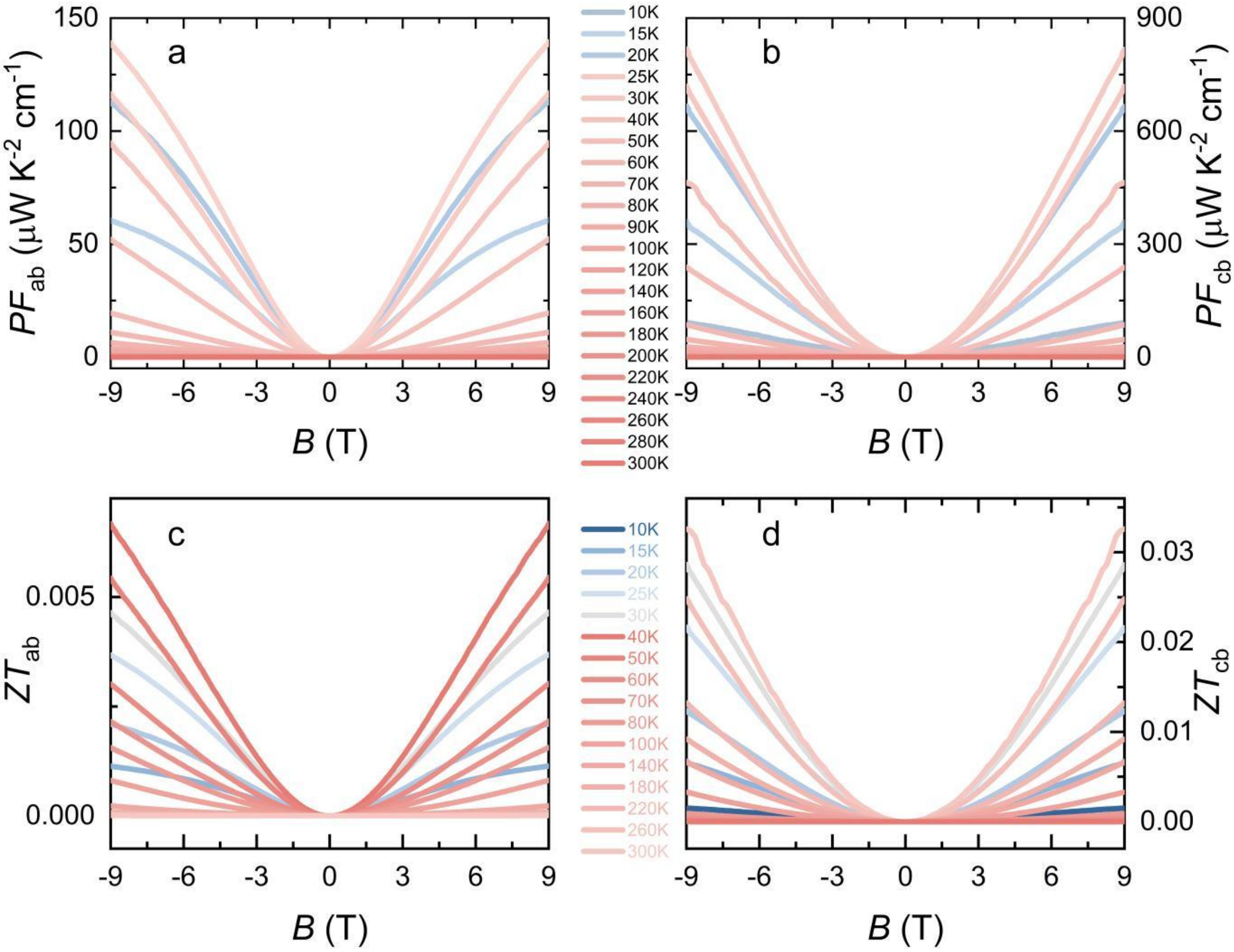

Supplementary Figure 9 | Power factors and *ZT*s. **a** and **c** The transverse power factor and *ZT* in the temperature range from 10 K to 300 K with the electric current applied along *a*-axis, the heat current along *b*-axis and the magnetic field along *c*-axis. **b** and **d** The transverse power factor and *ZT* in the temperature range from 10 K to 300 K with the electric current applied along *c*-axis, the heat current along *b*-axis and the magnetic field along *a*-axis.

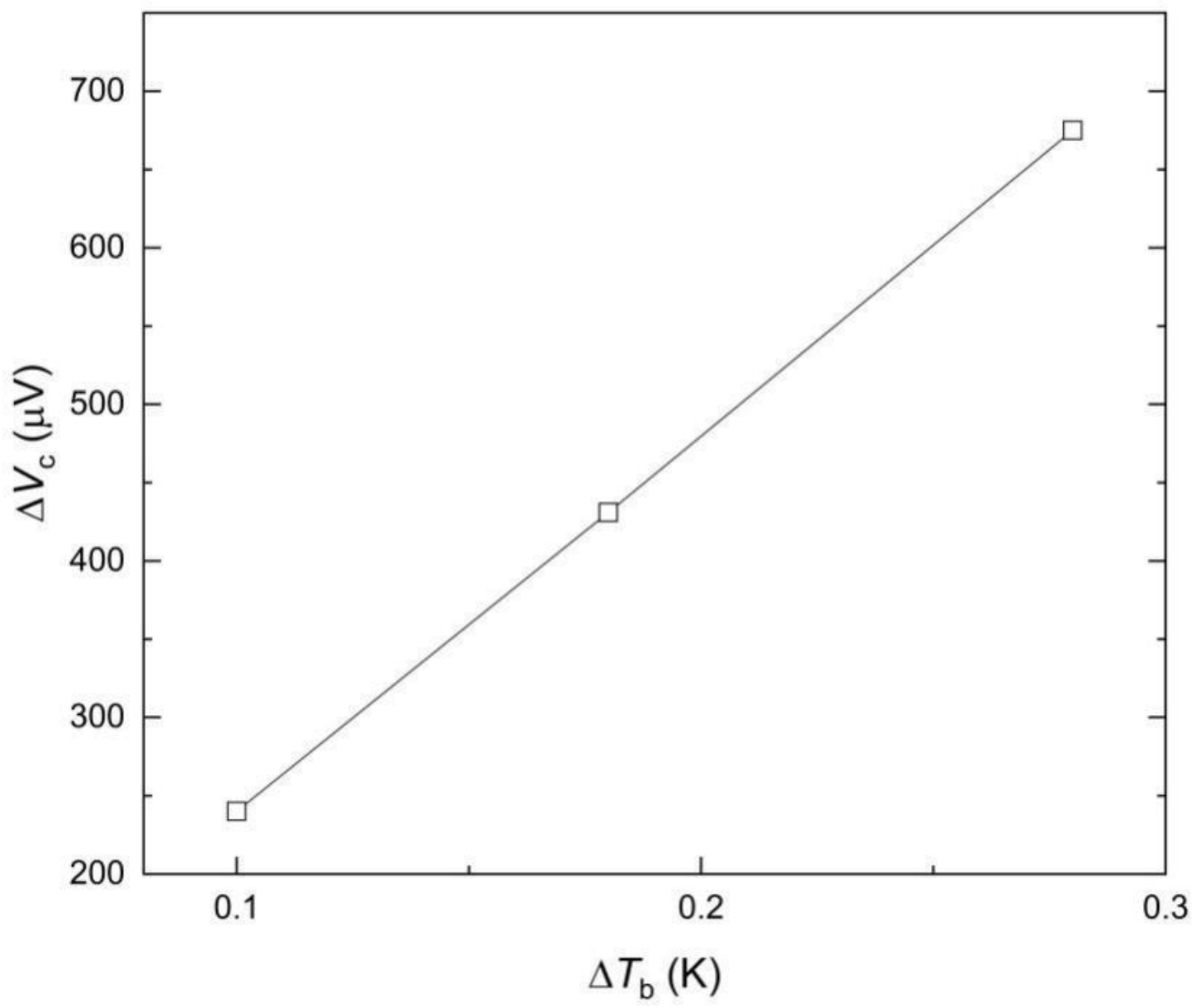

Supplementary Figure 10 | Extracted Nernst voltage signals versus different applied temperature gradients at 9 T and 25 K.

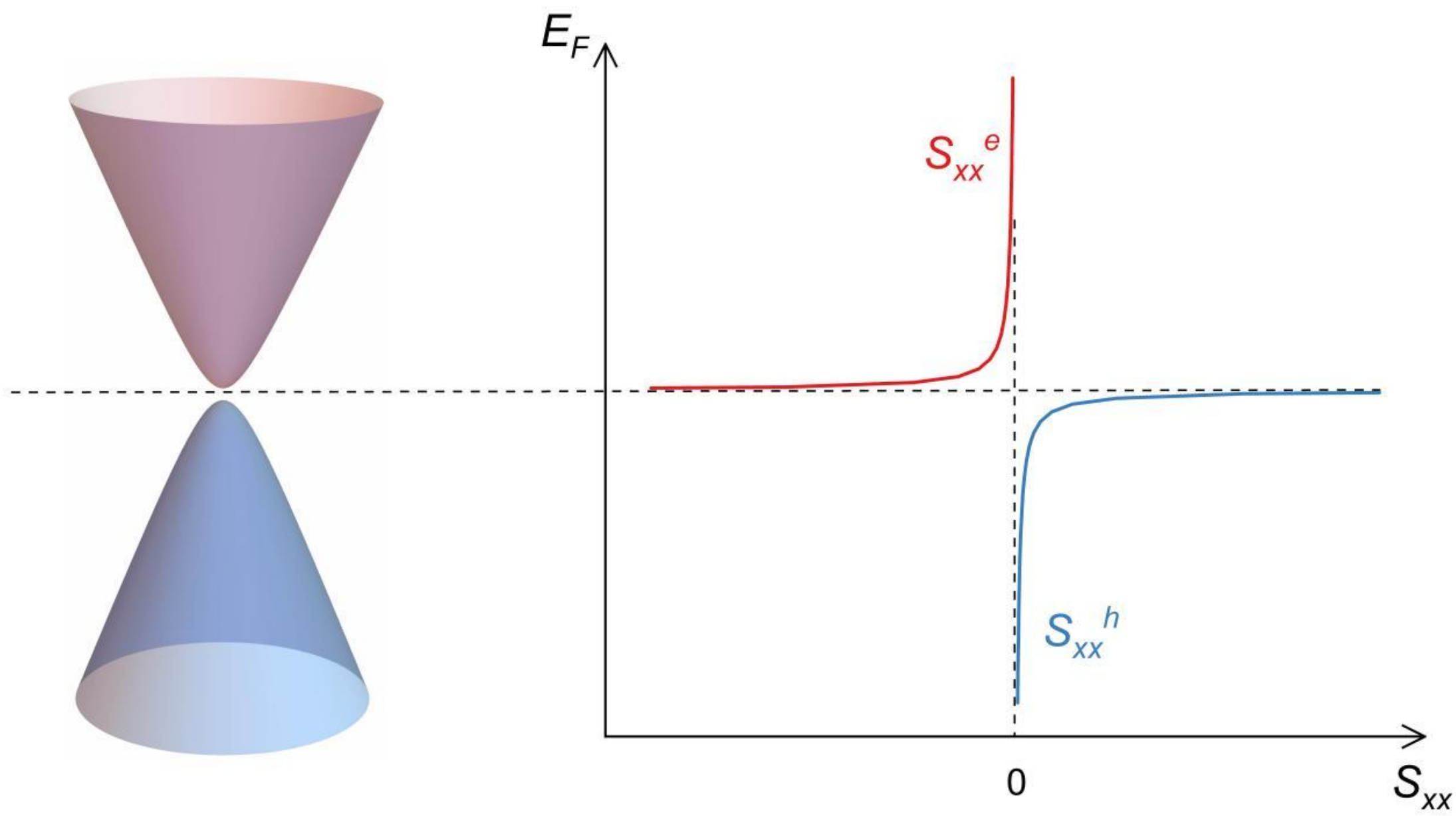

Supplementary Figure 11 | Partial Seebeck coefficient in topological semimetals.[6]

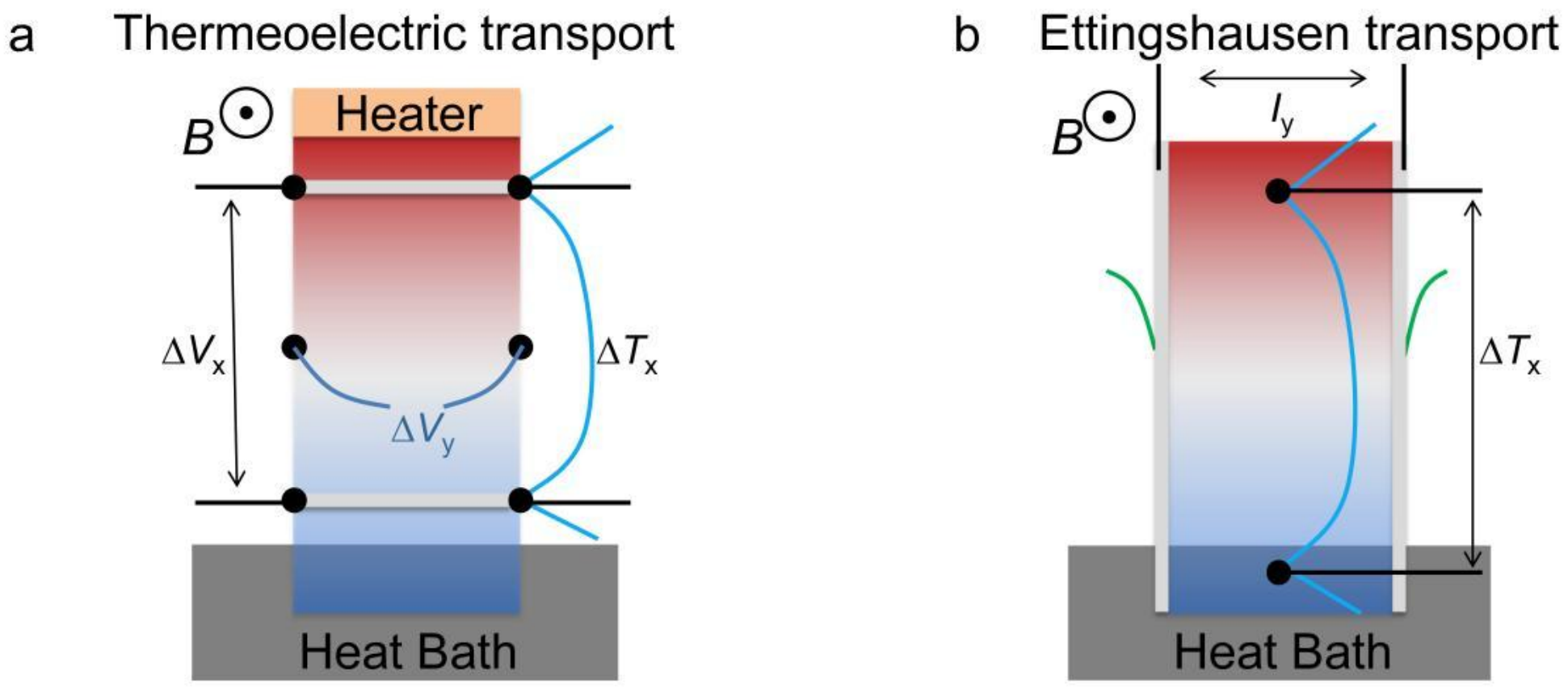

Supplementary Figure 12 | Schematic illustrations of the sample setup for thermoelectric/Ettingshausen transport properties measurements. The cold end of the sample was fixed a copper block as a heat sink. For thermal conductivity, Seebeck and Nernst coefficients (**a**), the hot end was attached to a copper heat spreader, and then a heater was mounted on the hot end to ensure uniform heat flow through the sample. A differential thermocouple was fixed along longitudinal direction to measure the temperature difference. Two pairs of platinum wires were attached to measure Seebeck and Nernst voltages. For Ettingshausen transport measurements (**b**), the same configuration was used, with the differential thermocouple aligned along the longitudinal direction to detect the temperature difference, while the electric current was applied along the transverse direction. Notably, silver paste was uniformly applied on the two transverse faces to ensure homogeneous current distribution along the transverse direction.

1. Cohn, J. L., dos Santos, C. A. M., and Neumeier, J. J. Superconductivity at carrier density 10^{17} cm^{-3} in quasi-one-dimensional $Li_{0.9}Mo_6O_{17}$. *Phys. Rev. B* **108**, L100512 (2023).
2. Chen, H., et al. Magnetotransport properties in purple bronze $Li_{0.9}Mo_6O_{17}$ single crystal. *Europhys. Lett.* **89**, 67010 (2010).
3. Xu, X. et al. Directional field-induced metallization of quasi-one-dimensional Li0.9Mo6O17, *Phys. Rev. Lett.* **102** 206602 (2009).
4. Marzari, N., et al. Maximally Localized Wannier Functions: Theory and Applications, *Rev. Mod. Phys.* **84**, 1419 (2012).
5. Mostofi, A. A., et al. An Updated Version of WANNIER90: A Tool for Obtaining Maximally-Localised Wannier Functions, *Comput. Phys. Commun.* **185**, 2309 (2014).
6. Vladyslav, K., Skinner, B. and Fu, L. Thermoelectric Hall conductivity and figure of merit in Dirac/Weyl materials. *Phys. Rev. B* **99**, 155123 (2019).
