# Supplementary material for: Observation of the amplified transverse thermoelectric signals by reduced-dimensionality transport anisotropy
Source: Nat Commun. 2026 Sep 8;17:9607. doi: 10.1038/s41467-026-77569-y (PMC13554294; doi:10.1038/s41467-026-77569-y)
Supplement: Supplementary file 2 [file 41467_2026_77569_MOESM2_ESM.pdf]

nature portfolio

Peer Review File

Observation of the amplified transverse thermoelectric signals by reduced-dimensionality transport anisotropy

Corresponding Author: Dr Honghui Wang

This file contains all reviewer reports in order by version, followed by all author rebuttals in order by version.

Version 0:

Reviewer comments:

Reviewer #1

(Remarks to the Author)
This manuscript reports a new strategy for enhancing the Nernst effect based on the anisotropic transport properties of low-dimensional structures. The paper is well organized, and the theory and experiments on the model material (LMO) are consistent. I judge that the overall quality is high.

Although giant transverse thermoelectric signals have been observed in LMO single crystals, this was partly reported in Ref. 30 (PRL 108, 056604 2012). Also, magneto-resistance, Hall effects, magneto-Seebeck effects, and Nernst effects in different crystallographic directions on this material were in part reported in PRB 108, L100512, 2023. I understand that the present manuscript includes new important results on transverse thermoelectric effects; however, based on these previous works, I would prefer not to recommend publication in Nat Commun. due to insufficient novelty. I believe that a more specialized journal would be preferable.

One technical suggestion is to provide experimental details about the thermoelectric measurements in the Methods section. Because the anisotropy is significant in this material, special care may be needed to precisely evaluate the transport quantities along each crystallographic direction.

Reviewer #2

(Remarks to the Author)
This manuscript reports a sizable transverse thermoelectric response in a low-dimensional material.
The observed large coefficients are sufficiently impactful; however, the relationship with the attributed mechanism is not entirely clear due to the ambiguous extraction of partial Seebeck coefficients.
Therefore, while the experimental results are systematic and appear solid, there are a few concerns that should be addressed before recommending publication.

The definition and estimation of S_drag are unclear, which raises concerns about logical circularity.
On Line 196, the authors state: “The partial Seebeck coefficient, extracted from extended Nernst equation (2) fitting, peaks at 25 K with pronounced contributions from both phonon drag and diffusion.”
Are the S_drag values obtained solely by fitting S_yx using Eq. 2, or can they be estimated independently from S_total? This clarification should be included in the main text.
If S_drag is obtained solely through fitting S_yx, the authors should provide a detailed explanation of the estimation procedure and include a discussion validating the S_drag values for both electrons and holes. Without this quantitative validation, it cannot be ruled out that the enhancement of S_yx may be simply attributed to S_yx_e and S_yx_h themselves.
If, on the other hand, S_drag can be independently estimated from S_total, the calculated Syx_ambipolar should be presented in the main text, preferably in graphical form.

It would also be helpful if the authors could include a discussion comparing the electrical conductivity of their material with that of bismuth (Bi), which exhibits the best and second-best Ettingshausen effects.
Since electrical conductivity influences Joule heating and is therefore critical for practical cooling applications, a discussion of the advantages and disadvantages of confined geometry in this context, as well as necessary future research directions,

would be valuable.

Additionally, there is a typo in Eq. 2: "amipolar" should be corrected to "ambipolar".

Reviewer #3

(Remarks to the Author)
The paper suggests that dimensional reduction together with the anisotropic Fermi surface geometry in 1D (in the quasi-1D LMO) amplifies the Nernst and Ettingshausen effects to levels substantially larger than those in higher dimensional materials, even up to 160 times. This will be interesting in engineering transverse thermoelectric materials for cryogenic cooling. However, the paper needs substantial revisions before acceptance. In general, the paper is not well written, with concepts scattered in various places without clear explanations. The following issues also need to be clarified regarding the explanation of the observed phenomena:

The main explanation arises from the fact that the structure is highly anisotropic and behaves essentially as 1D chains in the longitudinal direction, whereas mobility is suppressed in the other direction due to flattened bands.
But from Fig. 1b, it seems the flattened bands, which provide low-D features are below Ef, so why they are the ones that dominate the transverse signals? Relate to that, in Fig. 2d, at what energy is this energy surface drawn? How close to the Fermi level is it? Further, in Fig. S5 in the SI, the bandstructure is a bit confusing, because the S5a has dispersive features around the Fermi level, I assume at 0eV, but the energy surfaces are flat in S5b, and they are the main features that lead to the high signals reported. It will be better if the S5a was also plotted along some directions that indicate that 'flatness' as well.

Figure 1 explains the process, but it could have been prepared much better. For example, there is no clear immediate visible difference between the 1D and 3D schematic. Use different colors for the wave packets, make them more visible, and maybe magnify the effects a bit so the difference comes out. Also, in the text this should have been explained better. For example, which is the transport direction, where are the 1D chains (make then more evident in Fig. 1), and the scattering schematic events also look very similar. Clarify so the reader understands the setting. It is also confusing that the paper talks about 1D from the beginning, but it takes a while to explain that it is actually a quasi-1D within a 3D structure. The introduction needs reorganization for clarity.

The ZT is measured in S9, but I did not see a reference to the thermal conductivity. Please provide.

It is not clear how the data in Fig. 2f are fitted with the two-carrier model – what type of electronic structure is assumed above/below the metal insulator transition at 20K? How are the two merged? How does the electronic structure look like below 20K? Is it still quasi-1D?

The authors should quantify how much of the increase in the signals measured compared to 3D materials comes from the different effects described: low-D Seebeck, anisotropic mobility, phonon drag, etc. Specifically for the effect of low-dimensionality, for which the authors claim that it is one of the main contributors, I have the following concern:

The paper claims exotic 1D surface states and unconventional Fermi surfaces. In principle 1D surface states are not 'exotic', they are something studied for decades now and their only 'exotic' feature are the sharp features in their DOS. On the other hand, it is not clear what is a Fermi surface for 1D as we only have 1D electronic structure (this again goes back to the point about more clarity in the introduction about the studied material). The main concern is that sharp features in the DOS for thermoelectrics was the topic of investigation for many years, but it yielded no such performance enhancement. It is not clear what is different in this current work and they allow for such extraordinary performance. See for example [J. Appl. Phys. 105, 034506 (2009); Physical Review B, 83, 245305, (2011)], where it is explained why such features are not as useful as suggested. Figure S1 could support this; it is shown that by reducing Kt, essentially making the system more 1D, the Nernst thermopower only increases by 50% or so only. In that figure, it is also not clear why there is a maximum and not a monotonic increase as Kt reduces, making the system more 1D. Furthermore, how valid is the constant relaxation time approximation? This is crucial, because if one considers energy dependent scattering (which is the more correct approach), where the scattering rates are proportional to the density of states, then the energy dependencies of the scattering times and DOS cancel out, which makes transport at first order independent of dimensionality, and the low-D argument cannot be valid.

It seems more reasonable that reduced mobilities are mostly responsible for the signals observed. In 3D systems we use the Hall mobility, but in 1D the reduced mobility, which makes the difference.

When comparing quasi-1D and 3D, why does this system has an advantage with regards to mobility, is not clear. At low temperatures phonon scattering is suppressed, but it is suppressed in all systems.
Why is mobility higher in 1D than 3D?

In Fig. S8 in the SI, the S increases with lowering T, and this is attributed to low dimensions, but this is a typical case also for the 3D materials. The authors should plot what a 3D model predicts for S, versus what an 1D model predicts, and also produce the same with constant and energy dependent relaxation time approximations. Only if the difference is substantial, they can claim that 'exotic' features are responsible substantially for the improvements they observe.

Version 1:

Reviewer comments:

Reviewer #1

(Remarks to the Author)
The authors have carefully revised the manuscript in response to my previous comments. My major concern was the novelty of this study, as the magnetotransport effects of this material have already been reported in two papers. In the rebuttal letter, the authors claimed that the central advance of this work is the establishment of a general physical framework that explains how reduced dimensionality amplifies transverse thermoelectric responses. In this case, the question is how broadly this framework can be applied. This issue was clarified to some extent by the comments of Referee 3. This study will attract attention in related fields and stimulate future research. Therefore, I recommend the publication of this manuscript.

Reviewer #2

(Remarks to the Author)
The authors' response to most comments is reasonable and satisfactory, except for Comment 2.1. The description of the analysis background and physical model should be both concise and comprehensive in the introduction of the main text, as the current manuscript remains poorly organized and relies too heavily on implicit references to the supplementary material.

For the revision, the concern regarding Comment 2.1 should be addressed.
The authors claim that $S_{yx} = S_{yx\_ambipolar}$ is justified because the partial conductivities of electrons and holes are close in value. However, in Eq. 1, the first term, $(S_{yx}^e \sigma_{yy}^e + S_{yx}^h \sigma_{yy}^h)/\sigma_{yy}$, cannot be simply canceled because there is no information provided on the S_{yx} values for electrons or holes. When the partial conductivities are similar, it reduces to $S_{yx}^e + S_{yx}^h$, not their difference.

The authors should therefore clearly explain the magnitude and estimation of S_{yx} for both electrons and holes, and organize the introduction to provide the necessary physical context.
If S_{yx} for electrons and holes are both large, their sum may be significant and possibly overestimated even with a small difference in conductivities. Thus, a proper estimation of S_{yx} for electrons and holes is mandatory to justify their framework.

Reviewer #3

(Remarks to the Author)
The authors have addressed some of my concerns, but still some minor issues remain before publication:

In line 131, they authors need to specify how they solve the Boltzmann transport equation, and specifically what they assume for the scattering times and scattering processes.

The RTA in Fig. 1 should be noted as CRTA as is customary written, because RTA can be perceived as allowing energy dependence. Still, it is not clear why there is a difference between the RTA and the DPA, and which one is the appropriate one for this system?

Check the notation in Fig. 1b the Y direction is towards the flat direction, whereas in 2c it is in the linear direction with the Fermi level crossing the band, not below the band.

The legends of Fig. 2e are wrong. They are all squares, but the data symbols are triangles.

Line 321: the PF performance is large, but it is not better than other materials as shown in Fig. 4d. The authors should smoothen their claims.

The term ‘reduced dimensionality’ in the title tends to point towards the 1D DOS features. But the effect here observed is mostly due to the transport anisotropy. It is better is this is reflected in the title, rather than 1D. Maybe ‘reduced dimensionality transport anisotropy’. In the text it needs to be stated clearly: is it the 1D features that have some effect, or mostly the transport directionality. If both have an effect, then quantify how much of an effect each has. This point is still not clear.

The abstract is also somewhat deceiving. Although the signals can be some of the highest in their temperature range, the PF, which is the important quantity, is the one to be also mentioned, and that is comparable to the best materials measured in the literature (not highest). This needs to be smoothened.

Version 2:

Reviewer comments:

Reviewer #2

(Remarks to the Author)

The authors' response has addressed my concerns. The manuscript clearly demonstrates the high thermoelectric power and its origin due to low dimensionality.
Thus, I recommend its publication.
For better readability, it would be helpful if the authors could relate Sxy and S_xy^ambipolar (as well as the diffusion term), not only by text but also by equations.

Reviewer #3

(Remarks to the Author)
The authors have responded to my concerns adequately and I recommend publication.
The only remaining issue is that many symbols in the equations in the main paper but mostly in the SI do not appear correctly, but I would assume that these will be fixed during the proof stage.

Open Access This Peer Review File is licensed under a Creative Commons Attribution 4.0 International License, which permits use, sharing, adaptation, distribution and reproduction in any medium or format, as long as you give appropriate credit to the original author(s) and the source, provide a link to the Creative Commons license, and indicate if changes were made.
In cases where reviewers are anonymous, credit should be given to 'Anonymous Referee' and the source.
The images or other third party material in this Peer Review File are included in the article's Creative Commons license, unless indicated otherwise in a credit line to the material. If material is not included in the article's Creative Commons license and your intended use is not permitted by statutory regulation or exceeds the permitted use, you will need to obtain permission directly from the copyright holder.
To view a copy of this license, visit https://creativecommons.org/licenses/by/4.0/

Dear reviewers,

We sincerely thank the reviewers for your careful evaluation of our manuscript entitled "**Observation of the amplified transverse thermoelectric signals by reduced dimensionality**". We greatly appreciate your valuable and insightful comments. We believe that our detailed responses and the revised manuscript have adequately addressed your concerns and now meet the high standards of ***Nature Communications***.

Below please find the point-by-point response and the list of the changes that have been made in the revised manuscript.

Reviewer #1:

This manuscript reports a new strategy for enhancing the Nernst effect based on the anisotropic transport properties of low-dimensional structures. The paper is well organized, and the theory and experiments on the model material (LMO) are consistent. I judge that the overall quality is high.

Reply: We sincerely thank the reviewer for the positive evaluation of our work. We also greatly appreciate the time and effort devoted to reviewing our manuscript.

Comment 1.1

Although giant transverse thermoelectric signals have been observed in LMO single crystals, this was partly reported in Ref. 30 (PRL 108, 056604 2012). Also, magneto-resistance, Hall effects, magneto-Seebeck effects, and Nernst effects in different crystallographic directions on this material were in part reported in PRB 108, L100512, 2023. I understand that the present manuscript includes new important results on transverse thermoelectric effects; however, based on these previous works, I would prefer not to recommend publication in Nat Commun. due to insufficient novelty. I believe that a more specialized journal would be preferable.

Reply: We sincerely thank the reviewer for raising the important concern regarding novelty of our work in relation to previous studies on LMO. We acknowledge that earlier works, including *PRL* **108**, 056604 (2012) and *PRB* **108**, L100512 (2023), have reported anisotropic magnetotransport and thermoelectric properties in this material. However, these studies primarily focused on reporting large responses and anisotropic transport properties within the *ab* plane.

In contrast, the central advance of our work is not merely the observation of large transverse thermoelectric signals, but the establishment of a general physical framework that, to the best of our knowledge, explains how reduced dimensionality intrinsically amplifies transverse thermoelectric responses.

Specifically, our key advances are as follows:

1) **Extended Nernst formula and the concept of reduced mobility.**

We derive an extended Nernst expression：

$$S_{yx}^{ambipolar} = 1/2(S_{xx}^{h} - S_{xx}^{e})B_z\mu_H^2/\mu_{yy},$$

which goes beyond the conventional isotropic form and is applicable to strongly anisotropic and low-dimensional systems. This formula introduces the concept of "reduced mobility" (μ_H^2/μ_{yy}), demonstrating that, in low-dimensional systems, the carrier mobility (μ_{xx}) should be replaced by "reduced mobility" in describing the Nernst effect. Importantly, reduced mobility can be significantly enhanced in low-dimensional systems, leading to much larger Nernst responses than

in isotropic three-dimensional systems. This framework also explains how exceptionally large Nernst signals can arise even in systems with relatively low Hall mobility (Fig. 4c).

2) **Enhancement of the diffusive Seebeck contribution enabling large Nernst signals over a wide temperature range.**

Dimensional reduction enhances the diffusive component of the partial Seebeck coefficient by increasing the energy dependence of the density of states. This effect helps overcome the conventional limitation that large Nernst signals typically emerge only within a narrow temperature window below the onset of phonon–phonon Umklapp scattering, thereby enabling robust responses over a much broader temperature range.

3) New experimental observations beyond previous LMO studies.

While LMO serves as a platform to validate our framework of "low dimension-amplified transverse thermoelectricity," our measurements reveal a significantly enhanced Nernst coefficient in the *cb* plane (with *c* the least conductive axis and *b* the chain direction), which has not been reported previously and is second only to that of bismuth. In addition, we perform Ettingshausen measurements to further confirm the strong anisotropy between S_{cb} and S_{ab}, observing a record Ettingshausen response among reported materials.

Importantly, previous reports (*PRL* **108**, 056604 (2012); *PRB* **108**, L100512 (2023)) focuses on *ab* plane, where the anisotropy between S_{ab} and S_{ba} is expected and can be explained by Onsager reciprocal relation ($S_{ab}/\rho_a \approx S_{ba}/\rho_b$). In contrast, the pronounced anisotropy between S_{ab} and S_{cb} observed in our work highlights the critical role of low dimensionality and anisotropic geometry in amplifying transverse thermoelectric responses.

In conclusions, we demonstrate that dimensional reduction enhances the Nernst response not simply through high mobility or large partial Seebeck coefficients, but through a geometrical amplification mechanism governed by reduced mobility. We quantitatively show that this mechanism yields an enhancement exceeding 160 times compared to conventional 3D expectations, which cannot be explained within previously established isotropic frameworks. Moreover, previous studies on LMO did not identify or quantify this mechanism. They also did not establish a connection between reduced dimensionality and transverse thermoelectric amplification.

To clarify this distinction, we have added the reference ref.33(*PRB* **108**, L100512 (2023)) in the manuscript. We have substantially revised the discussion section to explicitly compare our work with previous reports and to highlight the advance of our work. We believe that this work goes beyond material-specific observations and provides a general design principle for transverse thermoelectrics.

Text changes in the manuscript:

Line 249: Furthermore, the tanΘ decreases monotonically with increasing temperature (see Fig. S7), is clearly distinct from the behavior of both $S_{ab}(T)$ and $S_{cb}(T)$, both of which exhibits a peak at 25 K. These observations indicate the dominance of the ambipolar term. Earlier studies have reported large S_{ab} and anisotropic transport properties focusing on *ab* plane[32,33], but overlooked the even larger S_{cb}, attributing the enhancement to a phonon drag-induced Seebeck electric field within an isotropic Nernst framework. However, this simplified isotropic formulation fails to capture the giant difference between S_{ab} and S_{cb} when the direction of heat flow is fixed. In our results, the dimensional reduction and phonon drag together yield large partial Seebeck electric field that persists over a wide temperature range (see Figs. 2e and 3h). Moreover, quasi-1D transport

properties also enhance Nernst thermopower via a extended Nernst equation (2), in which the reduced mobility embodies both intrinsic contributions from Fermi surface curvature K_m and extrinsic effects arising from scattering K_τ.

Comment 1.2

One technical suggestion is to provide experimental details about the thermoelectric measurements in the Methods section. Because the anisotropy is significant in this material, special care may be needed to precisely evaluate the transport quantities along each crystallographic direction.

Reply: We thank the reviewers for your constructive suggestions. We have added detailed descriptions of the thermoelectric measurements in the Methods section and included schematic illustrations in Fig. S13. The Nernst thermopowers S_{cb} and S_{ab} were measured with the heat flow applied along the *b*-axis, which exhibits the highest thermal conductivity among all crystallographic directions. This ensures a uniform heat flow along the *b*-axis and supports the reliability of the data. In addition, the Ettingshausen coefficient measurements, together with the corresponding Bridgman relation (see Fig. 3p), further confirm the reliability of the data.

Text changes in the Methods section of the manuscript:

<u>Transport measurement.</u> Electrical transport measurements, including resistivity and Hall resistivity, were carried out using a standard four-probe configuration on a Physical Property Measurement System (PPMS-9T, Quantum Design). Thermal/thermoelectric transport parameters were measured under high vacuum using a one-heater, two-(differential-)thermocouple steady-state method implemented within the same PPMS platform. To ensure uniform heat flow through the sample, thin copper sheets were attached at both ends of the sample. A strain gauge heater (~350 Ω) was then mounted on the hot end, while the cold end was fixed to a copper block with a piece of cigarette paper serving as electrical insulation. The differential thermocouple was fixed to the sample using black glue (Stycast 2850 FT). The Seebeck and Nernst voltages were measured by attaching two pairs of platinum wires to the sample with silver paste (see Fig. S13). During magnetic field sweeps at each temperature, measurements were conducted from 9 T to -9 T and back to 9 T. To eliminate self-inductance effect, the signals were symmetrized as $\Delta T(B) = \Delta T(-B) + \Delta T(+B)$; $\Delta V(B) = \Delta V(-B) + \Delta V(+B)$. For Ettingshausen temperature difference measurements, the same configuration was employed, with an electric current (25mA) applied along the *a*- and *c*-axes, the output temperature difference measured along *b*-axis, the hot end suspended, and the cold end fixed to the copper block.

Text and figure changes in the Supplementary material:

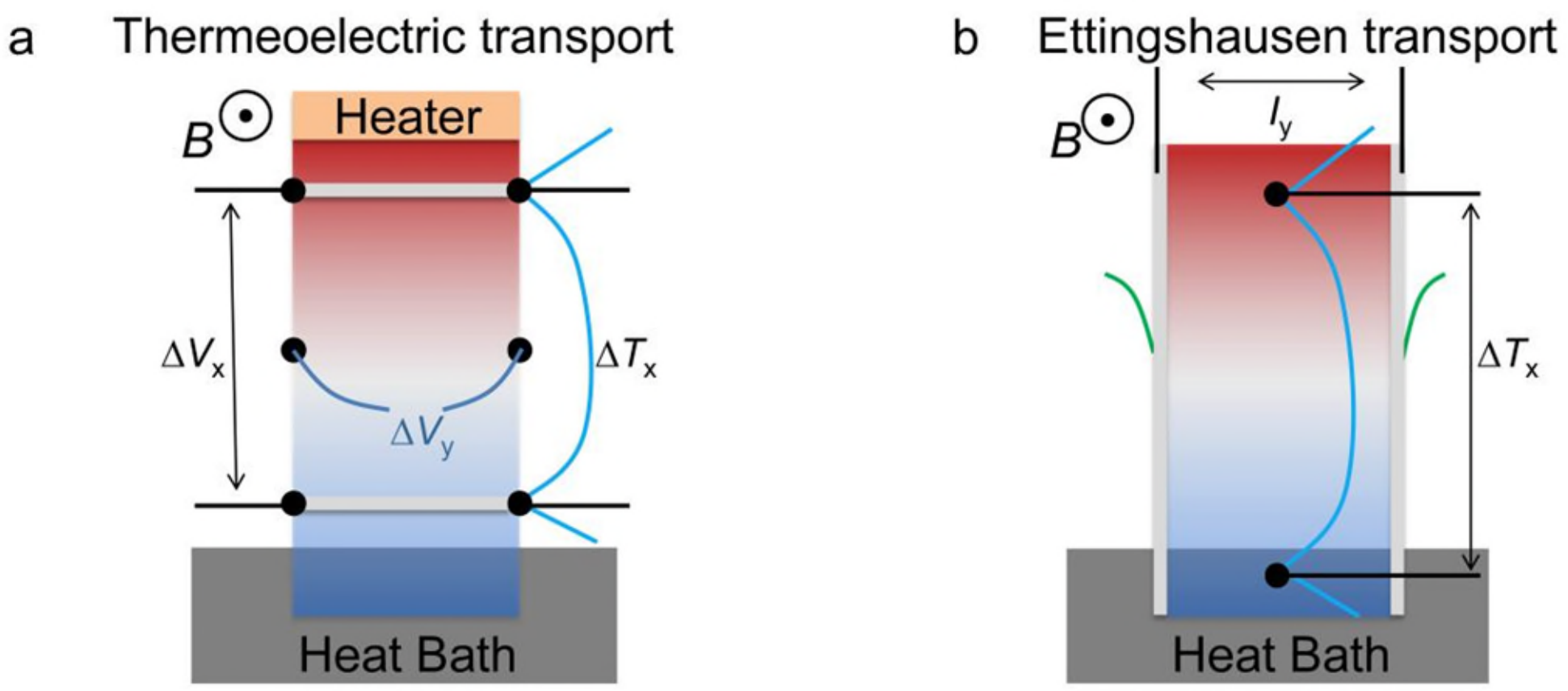

Fig. S13 Schematic illustrations of the sample setup for thermoelectric/Ettingshausen transport properties measurements. The cold end of the sample was fixed a copper block as a heat sink. For thermal conductivity, Seebeck and Nernst coefficients (**a**), the hot end was attached to a copper heat spreader, and then a heater was mounted on the hot end to ensure uniform heat flow through the sample. A differential thermocouple was fixed along longitudinal direction to measure the temperature difference. Two pairs of platinum wires were attached to measure Seebeck and Nernst voltages. For Ettingshausen transport measurements (**b**), the same configuration was used, with the differential thermocouple aligned along the longitudinal direction to detect the temperature difference, while the electric current was applied along the transverse direction. Notably, silver paste was uniformly applied on the two transverse faces to ensure homogeneous current distribution along the transverse direction.

Reviewer #2:

This manuscript reports a sizable transverse thermoelectric response in a low-dimensional material. The observed large coefficients are sufficiently impactful; however, the relationship with the attributed mechanism is not entirely clear due to the ambiguous extraction of partial Seebeck coefficients.

Therefore, while the experimental results are systematic and appear solid, there are a few concerns that should be addressed before recommending publication.

Reply: We thank the reviewer for your positive feedback on our experimental results and for raising this important suggestion regarding the unclear extraction of partial Seebeck contribution. In the revised manuscript, we have clarified how to obtain total, diffusion and phonon drag partial Seebeck coefficients. In additions, we have included the different dimensional model predicts for diffusive *S* under considering energy-dependent scattering and within the constant relaxation time approximation (see Fig. S1b). The dimensional reduction significantly enhance diffusive *S*.

Comment 2.1

The definition and estimation of S_drag are unclear, which raises concerns about logical circularity. On Line 196, the authors state: "The partial Seebeck coefficient, extracted from extended Nernst equation (2) fitting, peaks at 25 K with pronounced contributions from both phonon drag and diffusion."

Are the S_drag values obtained solely by fitting S_yx using Eq. 2, or can they be estimated independently from S_total?

This clarification should be included in the main text.

Reply: We thank the reviewer for pointing out our unclear expressions. With the help of the Nernst thermopower, we are able to resolve the total partical Seebeck contribution from the electrons and holes. From *Eq.2*, the slope of the Nernst thermopower with respect to magnetic field is proportional to the difference between (or the sum of the absolute values of) the partial electron and hole Seebeck coefficients. The difference between the total partial Seebeck coefficients S_{total}^h - S_{total}^e can be calculated through *Eq.2*, i.e.

$$S_{total}^h - S_{total}^e \approx 2S_{ab}/(\tilde{\mu}_{ab} B).$$

In addition, the sum of the partial Seebeck coefficients, $S_{total} = S_{total}^h + S_{total}^e$, can be directly obtained through Seebeck measurement (see Fig. 2e), since the partial conductivities of the electrons and holes are close to each other. By combining these two relations, we calculate the individual partial Seebeck coefficients S_{total}^h and S_{total}^e (see Fig. S8d). Based on DFT calculations, the diffusion components S_{diff}^e and S_{diff}^h are obtained. The phonon-drag contributions are then determined using

$$S_{drag}^e = S_{total}^e - S_{diff}^e,\ S_{drag}^h = S_{total}^h - S_{diff}^h.$$

We have corrected the manuscript accordingly and included the partial Seebeck coefficients in Fig. 2e and Fig. S8d for clarify.

Changes in Fig. 2e:

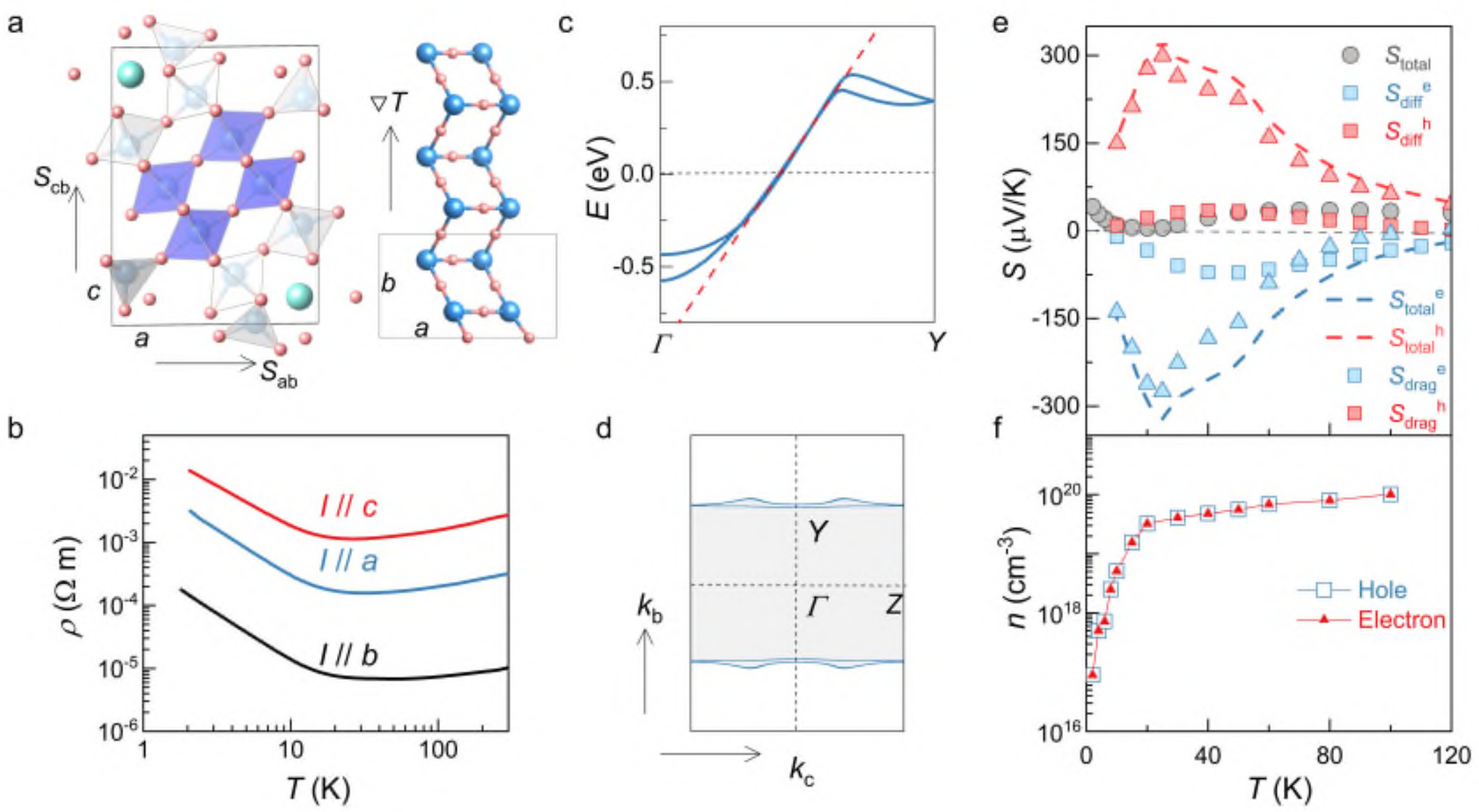

e Temperature dependence of Seebeck coefficients. A remarkably small net Seebeck coefficient (S_{bb} ~ 5 μV/K) is observed at 25 K, indicative of nearly perfect electron-hole compensation. The total partial Seebeck coefficients, extracted from extended Nernst equation (2) calculations (see Part 9 of the Supplementary Material for details), peaks at 25 K with pronounced contributions from both phonon drag and diffusion. The partial diffusion contributions are obtained through DFT calculations. The partial phonon-drag contributions are then obtained through subtracting the diffusion component from the total contribution. The diffusion term, strongly enhanced by reduced dimensionality, dominates above 70 K and extends the temperature window of large Nernst response. Even at 25 K, the diffusion contribution remains significant, reaching ~50 μV/K, owing to the quasi-1D properties.

Text and figure changes in Part 9 of Supplementary material:

For LMO, the partial diffusive Seebeck coefficients S_{diff}^h and S_{diff}^e (see Fig. S8a) are calculated based on the wannier tight-binding model, which are constructed based on the DFT calculation using wannier90 package,[3-4] with the Mo-*d* orbitals included. Fig. S8b shows the temperature dependence of Seebeck coefficients measured along *b*- and *a*-axes. To obtain the total partial Seebeck coefficients, the reduced mobility is first calculated in Fig. S8c and the difference between total partial Seebeck coefficients (S_{total}^h - S_{total}^e) is then determined using Eq. (2). Combined with the temperature-dependent S_b (S_b = S_{total}^h + S_{total}^e; Fig. S8b), the resulting total partial Seebeck coefficients are shown in Fig. S8d. The phonon-drag partial Seebeck contributions are obtained by calculating the diffusion contributions from the total partial Seebeck coefficients. The Nernst signals arising from different contributions are further distinguished in Figs. S8e-f. Figure S8e demonstrates that, under quasi-one-dimensional conditions, the introduction of reduced mobility significantly enhances the Nernst signal compared to that based on the Hall mobility. Figure S8f separates the phonon-drag and diffusive contributions to the Nernst signal. At low temperatures, the Nernst response is dominated by phonon drag, whereas with increasing temperature, the diffusive contribution gradually becomes more significant.

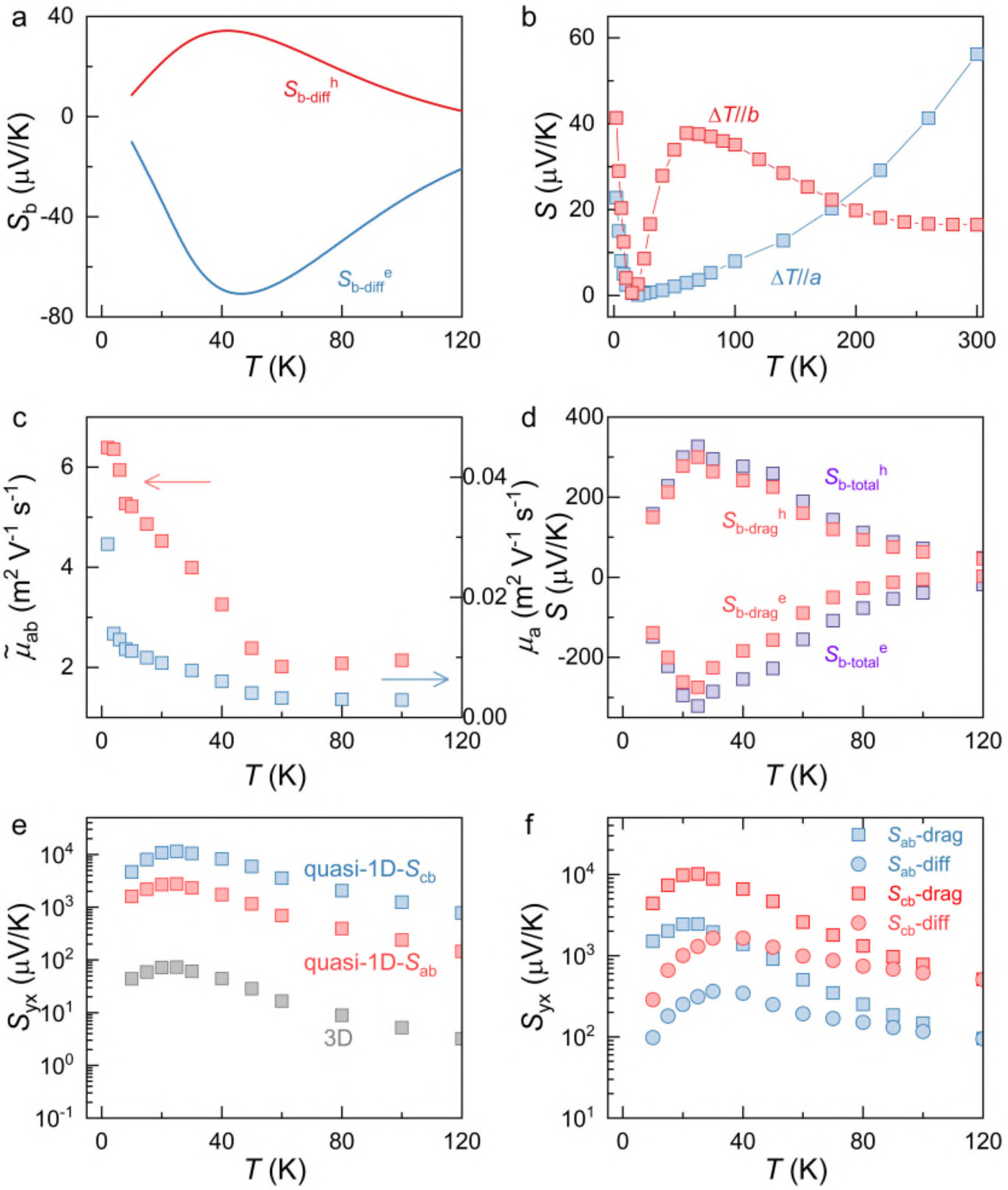

Fig. S8 | **a** Temperature dependence of the partial diffusive Seebeck coefficient, obtained through DFT calculations. **b** Temperature dependent Seebeck coefficients with the heat current applied along *b*- and *a*-axes, respectively. **c** Temperature dependence of the carrier mobility μ_a and the reduced mobility $\tilde{\mu}_{ab}$ (μ_{Hab}^2/μ_a). The μ_a is obtained through low field fitting of Fig. S4f. The Hall mobility μ_{Hab} is obtained through two-carrier model fitting. **d** Temperature dependence of the total partial Seebeck coefficients and the corresponding phonon-drag contributions. **e** The temperature-dependent Nernst signals S_{ab} and S_{cb} for quasi-one-dimensional LMO, compared with that of a three-dimensional case, where only the Hall mobility is considered and the reduced mobility is neglected. **f** Temperature-dependent Nernst signals in S_{ab} and S_{cb}, highlighting the respective contributions from phonon drag and diffusive Seebeck effects.

Comment 2.2

If S_drag is obtained solely through fitting S_yx, the authors should provide a detailed explanation of the estimation procedure and include a discussion validating the S_drag values for both electrons and holes. Without this quantitative validation, it cannot be ruled out that the enhancement of S_yx may be simply attributed to S_yx_e and S_yx_h themselves.

Reply: We apologize for the unclear expressions. As mentioned in our reply to Comment 2.1, we first calculate the total partial Seebeck coefficients, S_{total}^{h} and S_{total}^{e}, through equation (2) in combination with transport measurements. We then obtain the diffusion components S_{diff}^{e} and S_{diff}^{h} through DFT calculations. Finally, the phonon-drag partial Seebeck coefficients are obtained by $S_{drag}^{e} = S_{total}^{e} - S_{diff}^{e}$ and $S_{drag}^{h} = S_{total}^{h} - S_{diff}^{h}$. In part 8 of Supplementary material, we have included a quantitative analysis of the diffusion and ambipolar contributions (see Fig. S7b). Due to the small Hall angle and the relatively large Fermi energy (~500 meV), the diffusive term contributes negligibly to the Nernst response. Our results indicate that the ambipolar contribution dominates the large Nernst signal, while also confirming the validity of above extraction of the total partial Seebeck coefficients.

Text and figure changes in Part 8 of Supplementary material:

Hall angle is calculated by the formula $\tan\Theta = \sigma_{cb}/\sigma_{bb}$. Based on Mott relation and linear response equation, the diffusive Nernst contribution can be expressed as $S_{yx} = \pi^2/3(k_B^2T/e)\tan\Theta/\varepsilon$. Due to small Hall angle and the large Fermi energy (ε~500 meV), the diffusive Nernst component is negligibly small. The ambipolar Nernst component is obtained by subtracting diffusive contribution from the total signal. These results indicate that the ambipolar contribution dominates the large Nernst signal.

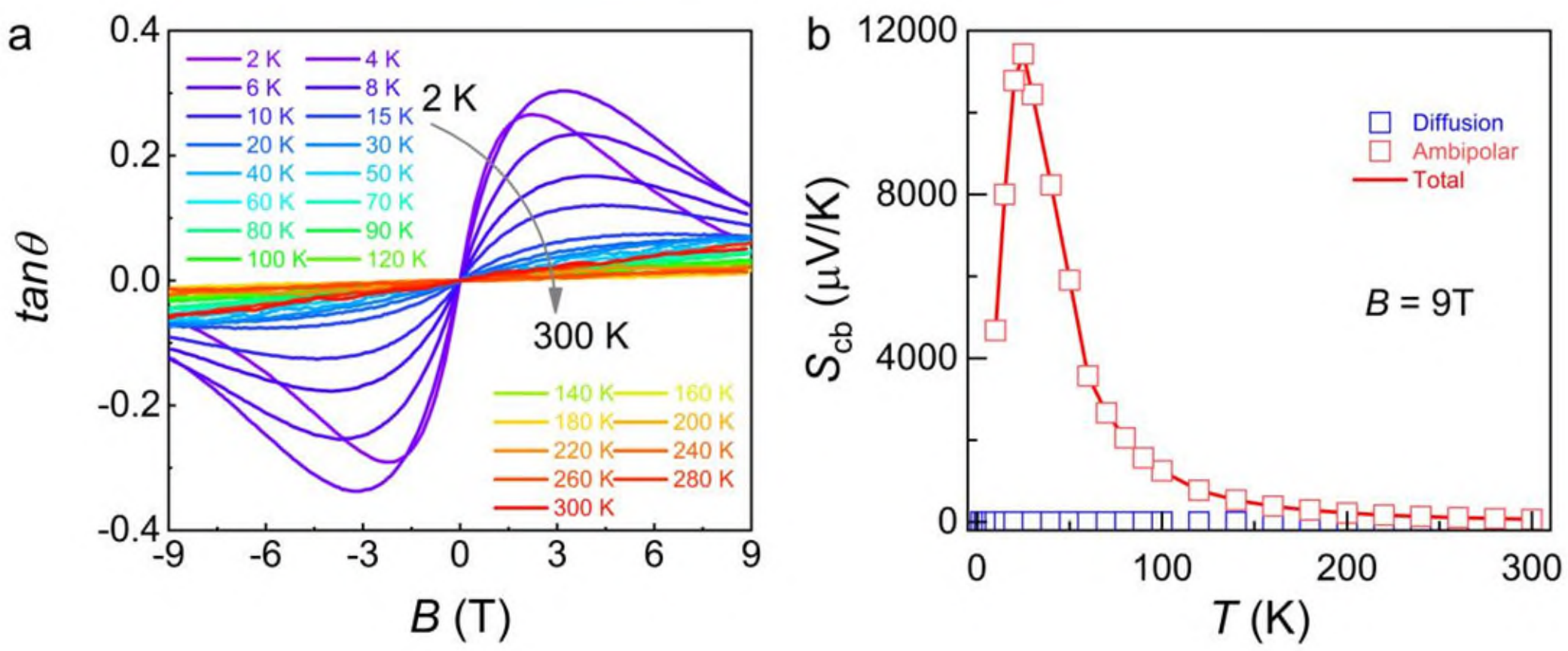

Fig. S7 | **a** The Hall angle σ_{cb}/σ_{bb} in the temperature range from 2 K to 300 K. **b** The quantitative analysis of the diffusion and ambipolar Nernst contributions. The diffusive component is obtained by the formula $S_{yx} = \pi^2/3(k_B^2T/e)\tan\Theta/\varepsilon$. The ambipolar contribution is obtained by subtracting diffusion contribution from the total signal.

Comment 2.3

If, on the other hand, S_drag can be independently estimated from S_total, the calculated Syx_ambipolar should be presented in the main text, preferably in graphical form.

Reply: We thank the reviewer for the valuable suggestion. The phonon-drag partial Seebeck coefficients are derived from the total partial Seebeck coefficients, S_{total}^{h} and S_{total}^{e}. First, we

determine S_{total}^h and S_{total}^e using Eq. (2) in combination with transport measurements. We then obtain the diffusive components S_{diff}^e and S_{diff}^h from DFT calculations. Finally, the phonon-drag contributions are extracted as $S_{drag}^e = S_{total}^e - S_{diff}^e$ and $S_{drag}^h = S_{total}^h - S_{diff}^h$. This approach is widely accepted to estimate the phonon-drag contribution (first obtaining the diffusive contribution, and then subtracting it from the total Seebeck coefficient to extract the phonon-drag component; ***Refs. R1-2***).

In addition, a direct quantitative evaluation of phonon-drag contribution requires solving the coupled electron-phonon Boltzmann transport equation. Although such first-principles approaches have been developed, they remain computationally demanding and achieving quantitative agreement with experiment is still challenging for large complex metallic system since several ingredients need to be accurately determined, including electron-phonon scattering matrix elements, phonon-phonon scattering, and boundary/impurity scattering.

References

[1] Li, P. et al. Colossal Nernst power factor in topological semimetal $NbSb_2$. *Nat. Commun.* **13**, 7612 (2022).

[2] Hu, H. et al. Multipocket synergy towards high thermoelectric performance in topological semimetal $TaAs_2$. *Nat. Commun.* **16**, 119 (2025).

Comment 2.4

It would also be helpful if the authors could include a discussion comparing the electrical conductivity of their material with that of bismuth (Bi), which exhibits the best and second-best Ettingshausen effects.

Since electrical conductivity influences Joule heating and is therefore critical for practical cooling applications, a discussion of the advantages and disadvantages of confined geometry in this context, as well as necessary future research directions, would be valuable.

Reply: We appreciate the reviewer's suggestion regarding comparison with bismuth (Bi), which further highlights the practical relevance of our work. In the revised manuscript, we have included a discussion comparing the electrical conductivity of LMO and Bi, along with future research directions aimed at further enhancing transverse thermoelectric signals and performance. While Bi exhibits extremely high carrier mobility and electrical conductivity at zero magnetic field, its conductivity is significantly suppressed under an applied magnetic field due to the Lorentz force. At the optimal magnetic field corresponding to the maximum Nernst signal, the conductivities of LMO and Bi become comparable, reaching the same order of magnitude. Therefore, strongly anisotropic (low dimensional) topological semimetals may represent more promising candidates for transverse thermoelectric applications, owing to their potentially optimized larger reduced mobility (compared with LMO) and lower magnetoresistivity along the low-mobility direction. Also, Bi shows the largest Nernst thermopower at very low T (approximately 10K), while LMO reaches the maximum at a much higher temperature.

Text changes in the manuscript:

Line 218: To further examine the role of transverse geometry with the heat flow direction held constant, we measured the Ettingshausen signals, i.e. the magnetic field induced transverse temperature gradient with the temperature difference recorded along *b*-axis (see Figs. 3i-o). The

Ettingshausen response shows a similar field dependence to that of the Nernst thermopower, displaying an approximately linear increase with increasing field strength across 24.5-102.2 K range. Maximum Ettingshausen signals reach 1.18×10^{-3} Km/A and 6.3×10^{-3} Km/A at 52.5 K and 43.1 K for electric currents along the *a*- and *c*-axis, respectively, which are comparable to or even surpass that of Bi (0.45×10^{-3} Km/A at 6.8 K).[28-29] While Bi exhibits extremely high mobility and conductivity at zero magnetic field, its conductivity is strongly suppressed under a magnetic field due to the Lorenz force, reaching the same order as that of LMO.[28-29] These results further underscore the exceptional Ettingshausen cooling capacity of LMO. Additionally, the Ettingshausen coefficient (E) and the isothermal Nernst thermopower (S_{yx}) are related via the Bridgman relation, $E_{xy} = S_{yx}T/\kappa_{xx}$,[23] where κ denotes the thermal conductivity and is independent of magnetic field in LMO. Figure 3p compares the Ettingshausen coefficients obtained via direct measurement with those calculated from the Nernst thermopower. The excellent agreement between the two provides compelling evidence for the pronounced difference in transverse TE response of LMO under fixed the directions of heat flow and electric current.

Line 320: In summary, we demonstrate that dimensional reduction provides an effective and previously under-explored strategy for enhancing transverse TE response and performance. Through a combination of theoretical modelling and experimental validation, we reveal that the record Nernst signal (11,430 μV/K at 25 K and 9 T) and Ettingshausen signal (0.0063 Km/A at 43.1 K and 9 T) observed in LMO originate not only from carrier compensation and high-mobility quasi-linear band dispersion, but more fundamentally from the emergence of exotic density-of-states distributions, pronounced Fermi-surface anisotropy, and direction-dependent scattering induced by dimensional reduction. These contributions are quantitatively captured by an extended Nernst framework (equation (2)) that couples the reduced mobility with enhanced Seebeck electric fields, offering a new foundation for understanding and optimizing transverse TE transport. Moreover, the interplay between phonon drag and dimensional-reduction-driven modifications of the density of states gives rise to a large Seebeck electric field sustained over a wide temperature range, overcoming the long-standing limitation that substantial transverse TE signals are restricted to narrow temperature windows. Consequently, LMO exhibits a remarkably large transverse power factor across a broad temperature interval, with a peak value of 814 μW cm^{-1} K^{-2}, while retaining excellent performance well below liquid-nitrogen temperatures. Its performance far exceeds that of most known transverse and even state-of-the-art longitudinal TE materials, underscoring its strong potential for low-temperature solid-state cooling. This work not only advances the fundamental understanding of low-dimensional transverse TE transport but also establishes a pathway towards the rational design of high-performance transverse TE materials for cryogenic applications.

Looking forward, the concept of reduced dimensionality opens multiple avenues for further advancing transverse thermoelectricity. A key challenge is achieving an optimal balance between an enhanced transverse response and sufficient electrical conductivity, particularly in transverse geometries where low mobility also limits charge transport. Beyond quasi-one-dimensional systems, future efforts may focus on low-dimensional topological semimetals, in which high reduced mobility, and large partial Seebeck coefficients coexist. Such platforms offer a promising route to simultaneously amplify Nernst and Ettingshausen effects while suppressing excessive magnetoresistance, thereby enabling more practical and efficient cryogenic cooling devices. More broadly, integrating dimensional engineering with band topology may establish a general design

paradigm for next-generation high-performance transverse TE materials.

Comment 2.5

Additionally, there is a typo in Eq. 2: "amipolar" should be corrected to "ambipolar".

Reply: We thank the reviewer for pointing out this typo. We have corrected “amipolar” to “ambipolar” in the revised manuscript.

Text changes in the manuscript:

$$S_{yx}^{ambipolar} = 1/2(S_{xx}^{h} - S_{xx}^{e})B_z\mu_H^2/\mu_{yy}, \quad (2)$$

Reviewer #3:

The paper suggests that dimensional reduction together with the anisotropic Fermi surface geometry in 1D (in the quasi-1D LMO) amplifies the Nernst and Ettingshausen effects to levels substantially larger than those in higher dimensional materials, even up to 160 times. This will be interesting in engineering transverse thermoelectric materials for cryogenic cooling. However, the paper needs substantial revisions before acceptance. In general, the paper is not well written, with concepts scattered in various places without clear explanations. The following issues also need to be clarified regarding the explanation of the observed phenomena:

Reply: We thank the reviewer for your positive feedback on our experimental results and for the valuable suggestions to improve our manuscript. In response, we have made extensive revisions, which are detailed below along with our point-by-point replies to your comments. Specifically, we carried out an explicit comparison between the 3D and 1D cases.

Comment 3.1

The main explanation arises from the fact that the structure is highly anisotropic and behaves essentially as 1D chains in the longitudinal direction, whereas mobility is suppressed in the other direction due to flattened bands.

But from Fig. 1b, it seems the flattened bands, which provide low-D features are below Ef, so why they are the ones that dominate the transverse signals?

Reply: We thank the reviewer for your valuable suggestions. Figure 1b illustrates the anisotropic Fermi surface (constant-energy surface) and band structure of a quasi-one-dimensional system along high-symmetry path. To improve clarity, we have introduced the high-symmetry points Γ and Γ`. The left side shows the dispersive band along the Γ-X direction, i.e., the quasi-one-dimensional chain direction, crossing the Fermi level at Γ` point. The right side present the less dispersive band along the perpendicular Γ`-Y` direction, corresponding to the interchain direction. Near the Fermi level, the coexistence of a highly dispersive band along the chain direction and a nearly flat band along the interchain direction jointly contributes to the pronounced transverse thermoelectric signals.

Text and figure changes in the manuscript:

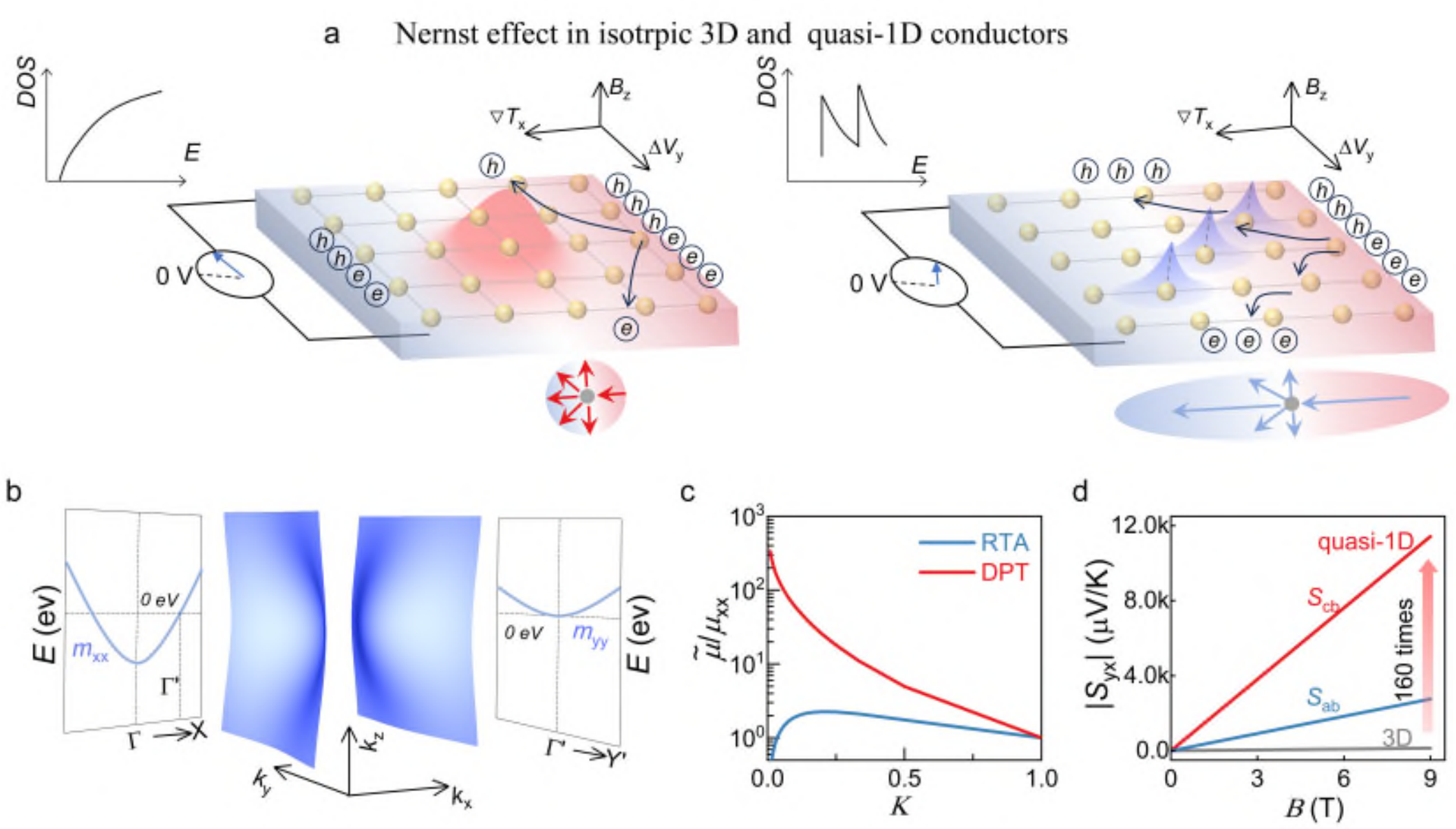

b Schematic of the strongly anisotropic effective mass distribution associated with a corrugated open Fermi surface in quasi-1D conductors. The Γ-X direction corresponds to the conducting chain, while Γ`-Y` denotes the interchain direction.

Comment 3.2

Relate to that, in Fig. 2d, at what energy is this energy surface drawn? How close to the Fermi level is it?

Reply: We apologize for the omission of the energy value. In Fig. 2d, the relevant energy is 0 meV. The DFT calculations initially provide the band structure for $LiMo_6O_{17}$. However, after accounting for Li vacancies ($Li_{0.9}Mo_6O_{17}$), the Fermi level shifts downward by 80 meV, yielding $E_F = -80$ meV. When plotting the band structure (Fig. 2c), we have shifted the energy scale upward by 80 meV, so that the Fermi level appears at 0 meV. Thus, the Fermi surface of LMO is also plotted at 0 meV.

To avoid confusion, we have explicitly distinguished between the Fermi level and the Fermi energy in the revised manuscript. The Fermi level is defined as $E_F = 0$ meV in the plotted bands and Fermi surface, while the Fermi energy $\varepsilon \approx 500$ meV corresponds to the energy difference between the Fermi level and the band bottom (Fig. 2c).

Comment 3.3

Further, in Fig. S5 in the SI, the bandstructure is a bit confusing, because the S5a has dispersive features around the Fermi level, I assume at 0eV, but the energy surfaces are flat in S5b, and they are the main features that lead to the high signals reported. It will be better if the S5a was also plotted along some directions that indicate that 'flatness' as well.

Reply: We thank the reviewers for your valuable suggestions. Distinguishing the band dispersions along different crystal orientations provides a more direct understanding of the quasi-one-dimensional nature of LMO. In the revised manuscript, we have added colored highlights to emphasize the bands along the Γ-Y, Γ-X, and Γ-Z. As shown in Fig. S5a, sharply dispersing bands cross the Fermi level along the Γ-Y direction. The dispersion relation weakens along the Γ-X direction and becomes nearly flat along the Γ-Z direction. Furthermore, the quasi-one dimensional property of LMO is also confirmed in Fig. S5b, presenting flat Fermi sheets in the XZ plane. For a better visualization of the band near the Fermi level, we additionally present band dispersions along Z1-Γ1-X1, Z2-Γ2-X2, Z3-Γ3-X3, and Z4-Γ4-X4. As shown in Fig. R1, near the Fermi energy, the bands exhibit weak dispersion along the *a*-axis and nearly flat along the *c*-axis, further confirming the pronounced quasi-one-dimensional nature of LMO.

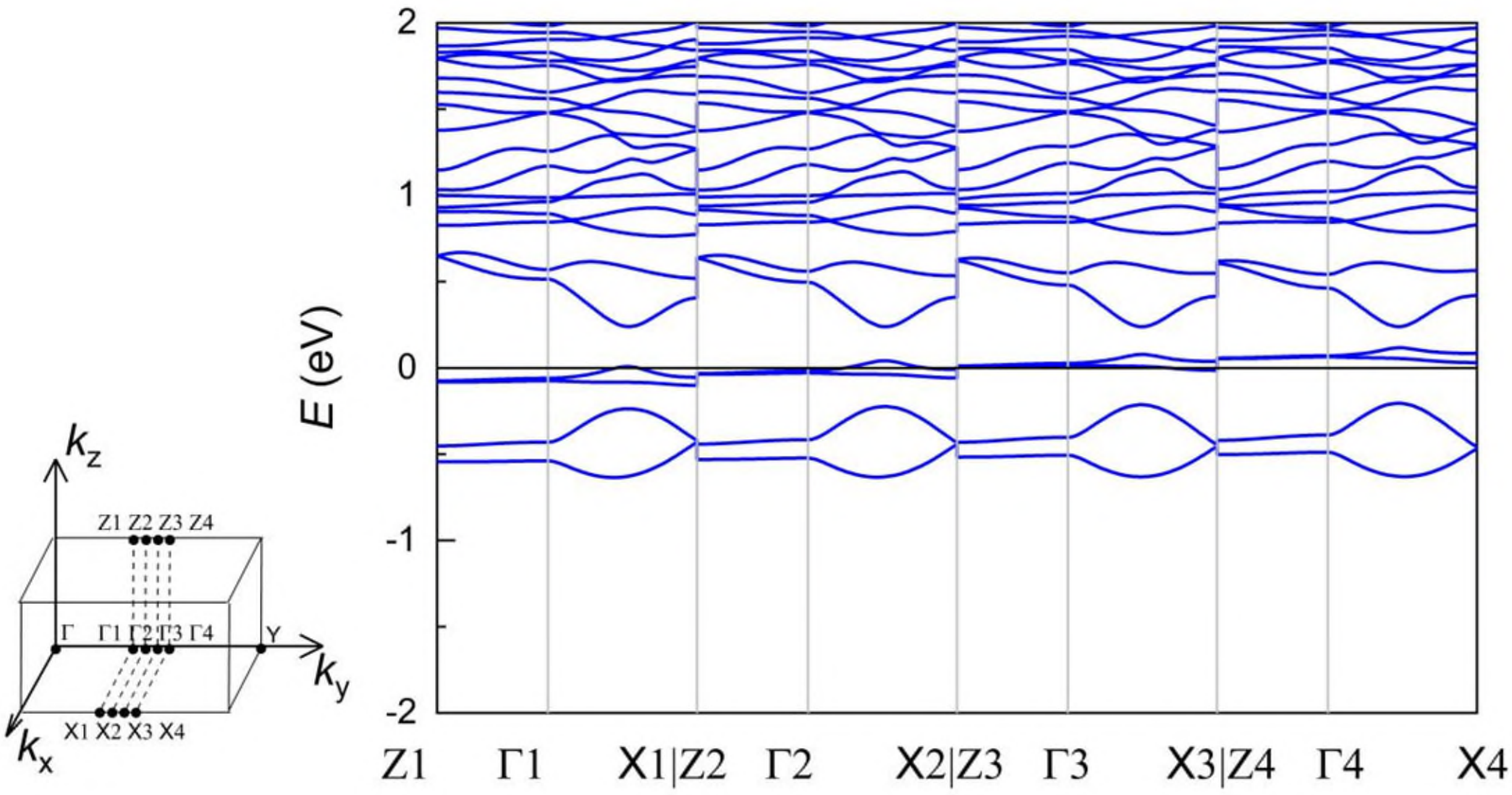

Fig. R1 | Evolution of the band structure near the Fermi level along *a*- and *c*-axes. The Γ1, Γ2, Γ3 and Γ4 are equal to 0.23, 0.24, 0.25 and 0.26, respectively.

Text and figure changes in Part 4 of Supplementary material:

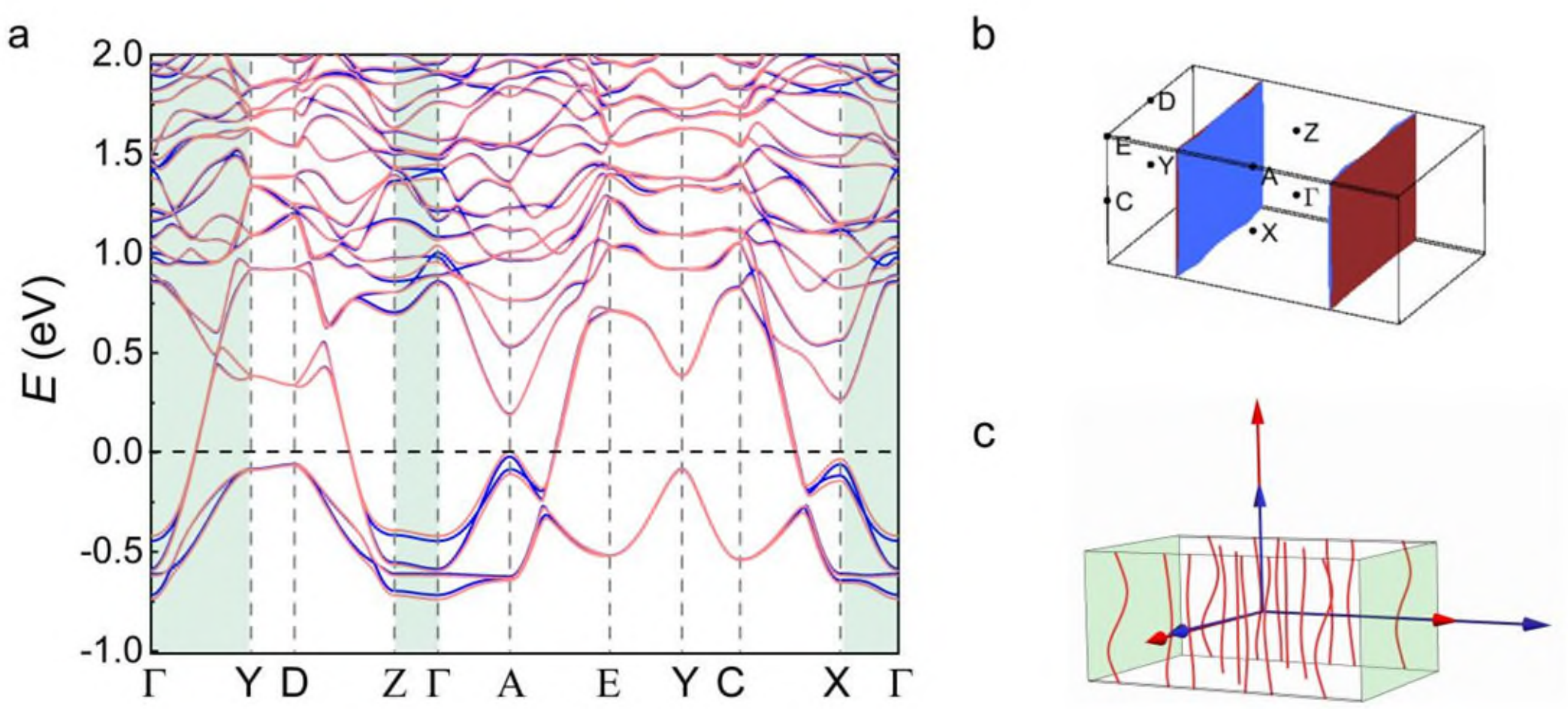

Fig. S5 | **a** Band structure for LMO without (blue) and with (red) SOC, which shows few differences as the bands are dominated by Mo 4*d* orbitals. The first-principle calculations were carried out based on Density functional theory implemented in Vienna ab initio Simulation Package (VASP) with the projector augmented wave (PAW) method. The exchange-correlation energy functional was treated using the Perdew-Burke-Ernzerhof (PBE) scheme within the generalized gradient approximation (GGA). The plane-wave energy cutoff of 500 eV was adopted. The energy and force convergence criteria were set to 10^{-7} eV and 10^{-5} eV Å−1, respectively. The Brillouin zone was sampled with the Gamma-centred k- mesh of 5×8×4. The three colour blocks represent the bands along the Γ-Y, the Γ-X and the Γ-Z, respectively. Along the Γ-Y direction, the sharp bands cut through the Fermi level. However, the dispersion relation weakens along the Γ-X and has even flattened out along the Γ-Z,

which further indicates the quasi-one-dimensional nature of LMO. **b** Fermi surface for LMO, which is composed of two pairs of nearly flat Fermi surface sheets, confirming the 1D feature of LMO system. **c** Nodal degeneracy due to the crossing of two bands close to the Fermi level. The green shade areas denotes the nodal surface at $k_y = \pi$ plane, and the red lines denote the nodal lines which are gaped out with the inclusion of SOC.

Comment 3.4

Figure 1 explains the process, but it could have been prepared much better. For example, there is no clear immediate visible difference between the 1D and 3D schematic. Use different colors for the wave packets, make them more visible, and maybe magnify the effects a bit so the difference comes out. Also, in the text this should have been explained better. For example, which is the transport direction, where are the 1D chains (make then more evident in Fig. 1), and the scattering schematic events also look very similar. Clarify so the reader understands the setting. It is also confusing that the paper talks about 1D from the beginning, but it takes a while to explain that it is actually a quasi-1D within a 3D structure. The introduction needs reorganization for clarity.

Reply: We thank the reviewers for your valuable suggestions. We have revised Fig. 1a to clearly distinguish the quasi-one-dimensional and three-dimensional wave packets, shown in blue and red, respectively. In addition, the directions of the one-dimensional chains and transport have been explicitly highlighted in the revised manuscript. Furthermore, we have clarified the differences in scattering between quasi-one-dimensional systems (characterized by strong anisotropy) and three-dimensional systems (typically isotropic). The Introduction has also been substantially revised to emphasize that the system under study is a quasi-one-dimensional bulk system, namely, a quasi-one-dimensional electronic system embedded within a three-dimensional crystal structure.

Text and figure changes in the manuscript:

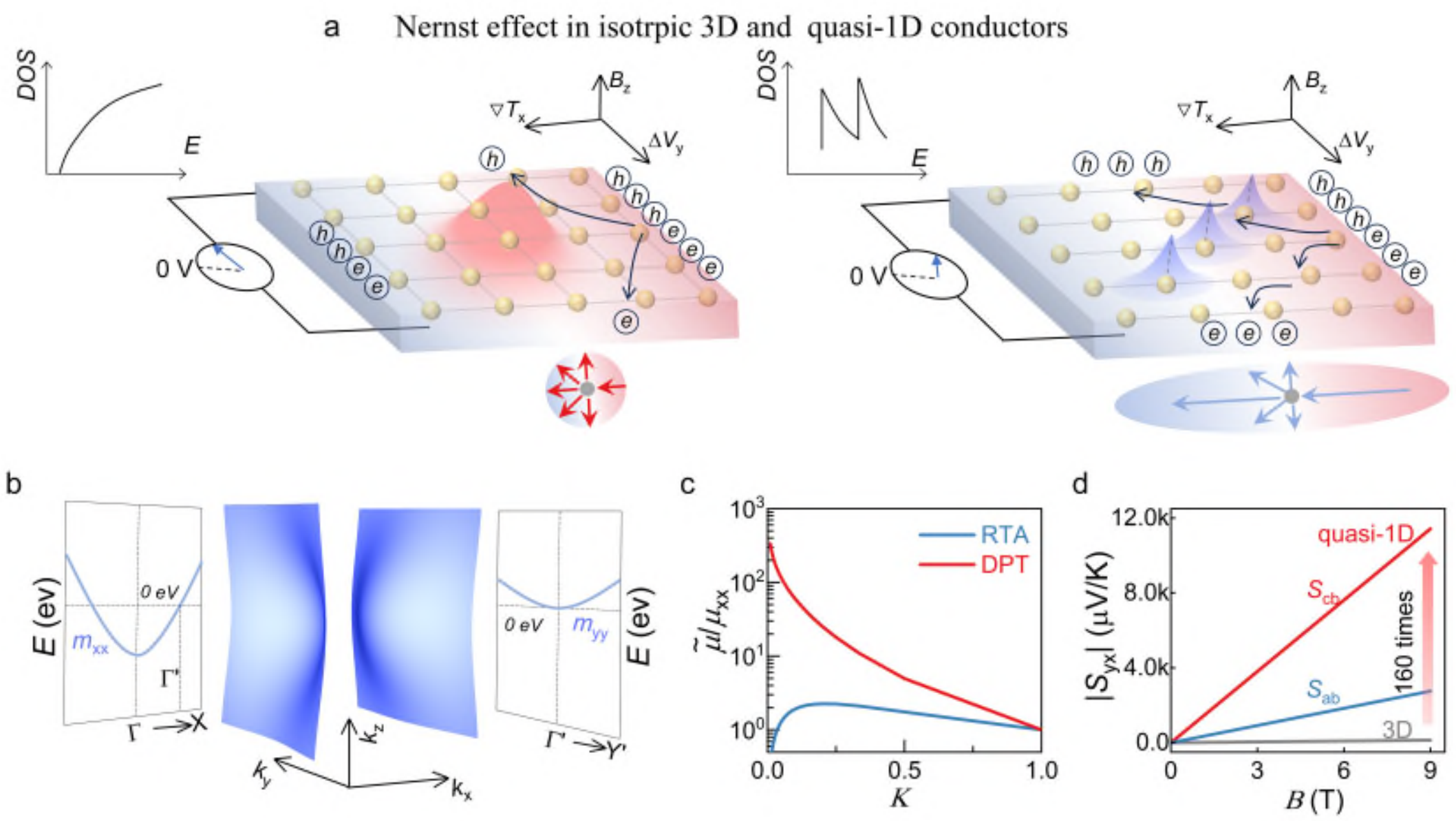

a Schematic illustration of the Nernst effect in an isotropic 3D (left) and a quasi-1D conductor (right). When the same heat flux is applied along *x*-axis and the same orthogonal magnetic field along *z*-axis, the resulting Nernst voltage along *y*-axis differs significantly between quasi-one-

dimensional and three-dimensional systems. In 3D systems, atomic orbital overlap (red wave packet) is comparable along all crystallographic axes, yielding nearly isotropic effective masses ($m_{xx}^* \approx m_{yy}^*$), relaxation times ($\tau_{xx} \approx \tau_{yy}$) and mobilities ($\mu_{xx} \approx \mu_{yy} \approx \mu_H$). By contrast, quasi-1D conductors (conducting chains along *x*-axis) display pronounced anisotropy: strong orbital hybridization dominates along the chain direction, whereas inter-chain coupling remains weak and yields strong electron localization (blue wave packets). This anisotropic coupling landscape leads to large disparities in effective mass and relaxation time, and thus direction-dependent carrier mobility. Consequently, the electronic entropy δ ($\delta \sim m^*$) is larger for inter-chain transport in quasi-1D systems than in 3D ones,[24] leaving a greater residual charge. Together with an enlarged Seebeck-driven electric field arising from the exotic density of states (top left; continuous energy-dependent density of states in 3D conductors and nearly δ-shaped energy-dependent density of states in quasi-1D conductors), these factors markedly amplify the Nernst response.

Text changes in the manuscript:

Line 63: In transverse TE geometry, the thermal gradient, electric current (or voltage), and magnetic field are mutually orthogonal (see Fig. 1a). This geometric decoupling of thermal and charge transport simplifies device structures[21-22] and introduces additional degrees of freedom for material optimization that are absent in longitudinal configurations. Large Nernst responses have been reported in various correlated and topological materials,[7-22] which are contributed by the phonon drag induced high partial Seebeck coefficients (S_{xx}^h; S_{xx}^e) as well as the high mobilities. Additional underlying mechanisms between transverse geometry and enhanced TE effects, particularly in highly anisotropic systems remain insufficiently explored. Further enhancement of the Nernst thermopower naturally requires higher partial Seebeck coefficients. Quasi-one-dimensional (quasi-1D) conductors intrinsically host exotic electronic density of states and strongly anisotropic open Fermi surfaces, features that may profoundly reshape transverse TE responses. Developing a systematic framework that links quasi-1D electronic anisotropy and macroscopic TE performance is therefore essential for advancing both fundamental understanding and device design. In this work, we identify enhanced geometrical anisotropy as a guiding design principle for amplifying transverse TE responses. Through a combination of theoretical modelling and experimental investigation on bulk quasi-1D $Li_{0.9}Mo_6O_{17}$ (LMO), we show that low-dimensional structures not only generates a Seebeck-driven electric field larger than that in higher dimensional systems but also enhance the reduced mobility ($\tilde{\mu}$) through introducing anisotropic Fermi surface geometry and direction-dependent scattering. These effects act synergistically to yield large Nernst and Ettingshausen signals, exceeding state-of-the-art transverse TE materials. These findings establish geometrical optimization as a new framework for transverse TE engineering, opening avenues towards high-performance cryogenic cooling technologies.

Comment 3.5

The ZT is measured in S9, but I did not see a reference to the thermal conductivity. Please provide.

Reply: We apologize for the missing magneto-thermal conductivity data. It has now been added in Fig. S3c.

Text and figure changes in Part 4 of Supplementary material:

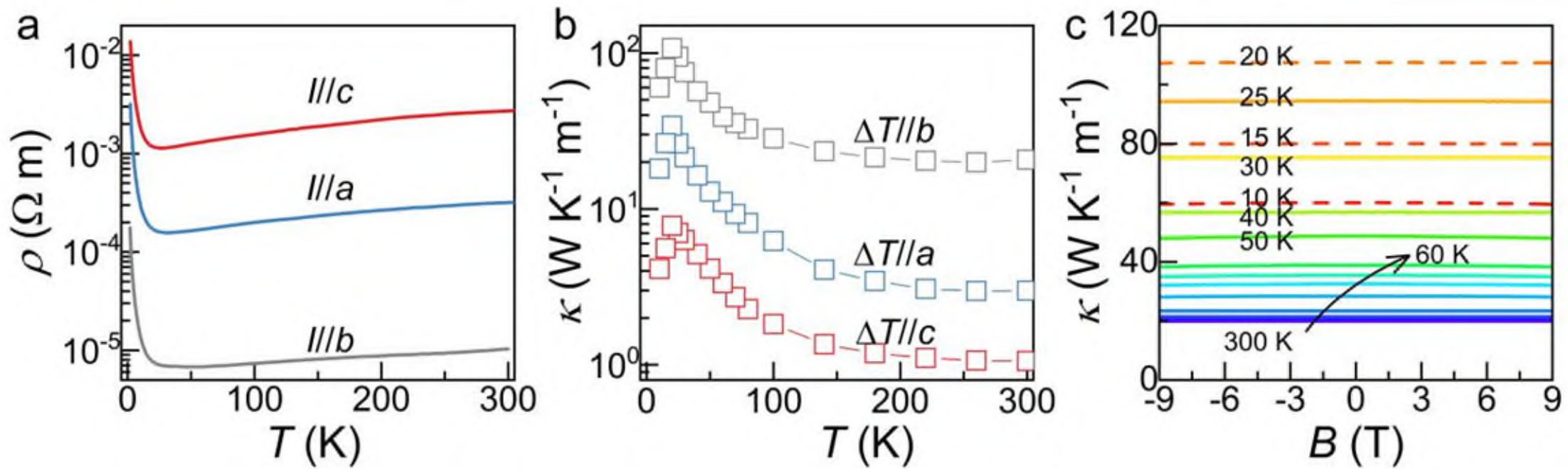

Fig. S3 | a Anisotropic electrical resistivity and **b** thermal conductivity measured with the electric current and heat current applied along the *b*-, *a*-, and *c*-axes, respectively. **c** Magnetic field dependence of the thermal conductivity along *b*-axis over the temperature range from 10 K to 300 K.

Comment 3.6

It is not clear how the data in Fig. 2f are fitted with the two-carrier model – what type of electronic structure is assumed above/below the metal insulator transition at 20K? How are the two merged? How does the electronic structure look like below 20K? Is it still quasi-1D?

Reply: Temperature-dependent carrier concentrations (Fig. 2f) and Hall mobility (Fig. 4a) extracted from two-carrier model fitting $\sigma_{yx} = eB(\frac{n_1\mu_1^2}{1+\mu_1^2B^2} - \frac{n_2\mu_2^2}{1+\mu_2^2B^2})$, where the Hall conductivity $\sigma_{ab} \approx \rho_{ab}/\rho_a\rho_b$ and $\sigma_{cb} \approx \rho_{cb}/\rho_c\rho_b$ due to $\rho_a\rho_b >> \rho_{ab}\rho_{ba}$ and $\rho_c\rho_b >> \rho_{cb}\rho_{bc}$.

The electronic structure above ~20 K is obtained from DFT calculations (see Fig. 2c and Fig. S5a). The Fermi surface of LMO along the conducting chain direction (Γ-Y) is primarily composed of two half-filled quasi-one-dimensional bands, which give rise to four nearly flat Fermi surface sheets (Fig. 2d). Each sheet contains regions of both positive and negative curvature, resulting in nearly perfect compensation between electrons and holes. Therefore, it is reasonable to extract carrier concentrations and mobilities using a two-carrier model.

Below ~20 K, several mechanisms, including charge-density waves (CDW), spin-density waves (SDW), dimensional crossover and exciton order (***refs. R3-5***), have been proposed to explain the metal-insulator transition. However, no consensus has yet been reached. Nevertheless, based on our results, the resistivity anisotropy remains pronounced below 20 K (Fig. R2c), indicating that the system seems to preserve its quasi-one-dimensional character, although the resistivity ratio gradually decrease with decreasing temperature. These correlation effects may open a small gap near the Fermi level. A gap fitting based on the relation $\rho_b = \rho_0 e^{[Eg/(2kBT)]}$ is shown in Fig. R2a (***ref. R4***), yielding an extremely small gap of approximately 0.94 meV. A schematic diagram of the band structure is also shown in Fig. R2b (red). Thus, the system remains approximately compensated, making the two-carrier model still applicable for extracting transport parameters.

The key evidence to this concern is that, the resistivity anisotropy remains pronounced below ~20 K (Fig. R2c), suggesting that the system preserves its quasi-one-dimensional nature.

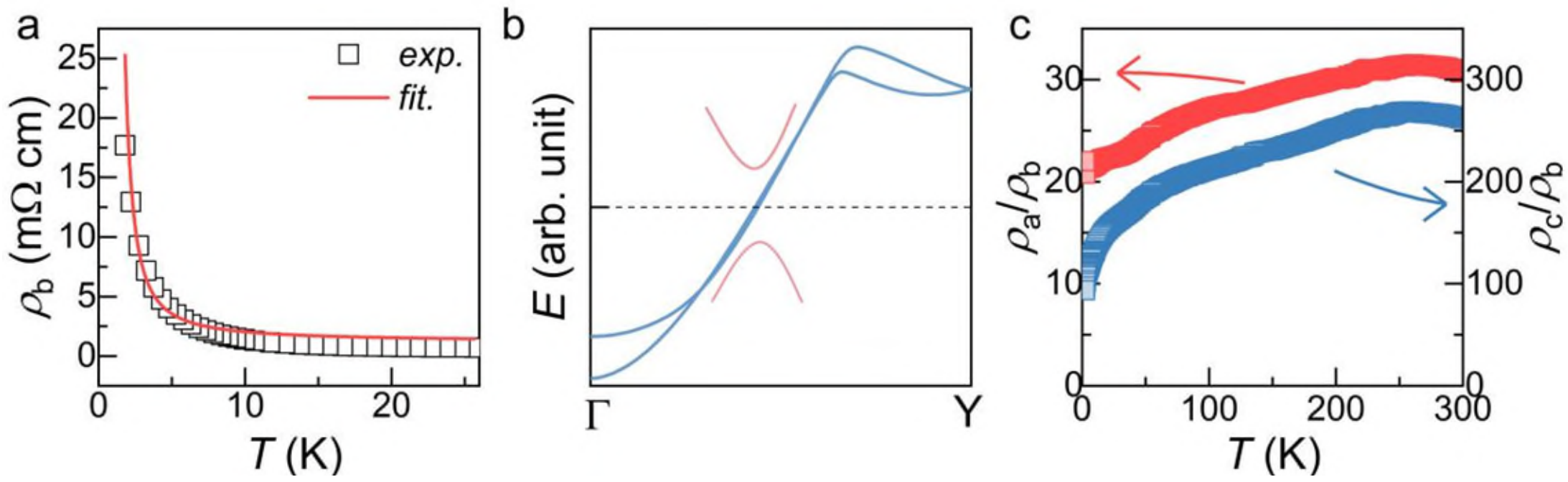

Fig. R2 | a Temperature dependence of resistivity along *b*-axis. The red line is fitted by formula $\rho_b = \rho_0 e^{[E_g/(2k_BT)]}$. **b** The electronic band structure along *b*-axis. Above ~20 K (blue), The band structure is obtained by DFT calculation. Below ~20 K, The red line indicates the band. Due to correlation effects, a small gap has formed near the Fermi level. **c** Temperature dependence of the resistivity ratios ρ_a/ρ_b and ρ_c/ρ_b. Although the resistivity ratio tends to decrease as the temperature decreases, there is still strong anisotropy at low temperatures ($\rho_a/\rho_b \approx 21$ and $\rho_c/\rho_b \approx 97$ at 2 K), indicating that the quasi-one-dimensional nature of LMO persists across the entire temperature range.

Reference

3. Dos Santos, C. A. M. et al. Dimensional crossover in the purple bronze $Li_{0.9}Mo_6O_{17}$, *Phys. Rev. Lett.* **98** 266405 (2007).
4. Xu, X. et al. Directional field-induced metallization of quasi-one-dimensional Li0.9Mo6O17, *Phys. Rev. Lett.* **102** 206602 (2009).
5. Lu, J. et al. Emergence of a real-space symmetry axis in the magnetoresistance of the one-dimensional conductor $Li_{0.9}Mo_6O_{17}$. *Sci. Adv.* **5**, eaar8027 (2019).

Text changes in Part 5 of Supplementary material:

Magnetoresistivity and Hall resistivity are shown in Figs. S4a–f. Based on the compensated band structure along the conducting chain (DFT calculations indicate that, in the metallic regime, the Fermi surface of LMO is primarily composed of two half-filled quasi-one-dimensional bands. These bands give rise to four nearly flat Fermi surface sheets (Fig. 2d). Each sheet contains regions of both positive and negative curvature, resulting in nearly perfect compensation between electrons and holes. However, due to possible correlation effects, the system undergoes a metal-insulator transition with the opening of a gap below ~25 K, while compensated electrons and holes may still persist.[3]), the temperature-dependent carrier concentrations (Fig. 2f) and Hall mobility (Fig. 4a) are extracted using a two-carrier model $\sigma_{yx} = eB(\frac{n_1\mu_1^2}{1+\mu_1^2B^2} - \frac{n_2\mu_2^2}{1+\mu_2^2B^2})$. In this analysis, the Hall conductivity can be approximated as $\sigma_{ab} \approx \rho_{ab}/\rho_a\rho_b$ and $\sigma_{cb} \approx \rho_{cb}/\rho_c\rho_b$, given that $\rho_a\rho_b >> \rho_{ab}\rho_{ba}$ and $\rho_c\rho_b >> \rho_{cb}\rho_{bc}$.

Text changes in the manuscript:

Line 193: By contrast, a sharp decline in both carrier types occur below 20 K, which potentially indicates the onset of possibly correlated effects associated with the metal-insulator transition in

$\rho(T)$.[27-29]

Comment 3.7

The authors should quantify how much of the increase in the signals measured compared to 3D materials comes from the different effects described: low-D Seebeck, anisotropic mobility, phonon drag, etc. Specifically for the effect of low-dimensionality, for which the authors claim that it is one of the main contributors, I have the following concern:

Reply: We thank the reviewers for your valuable suggestions. First, we have included a quantitative analysis separating the diffusive and ambipolar contributions (see Fig. S7b), which demonstrates the dominant role of the ambipolar component. In addition, quantitative analyses of the low-dimensional Seebeck effect, anisotropic mobility (reduced mobility), and phonon-drag contributions are presented in Figs. S8e-f. As described by the extended Nernst formula, $S_{yx}^{ambipolar} = 1/2(S_{xx}^{h} - S_{xx}^{e})B_z\mu_H^2/\mu_{yy}$, both the partial Seebeck coefficients (S_{xx}^{h} - S_{xx}^{e}) and the reduced mobility (μ_H^2/μ_{yy}) play essential roles in generating a large Nernst signal, with a multiplicative coupling between them. Therefore, both contributions are crucial. That is, strong anisotropy in low dimensional systems induces a large reduced mobility. Low-dimensional density of states and phonon drag increase the partial Seebeck effect. The low-dimensional Seebeck contribution gradually dominates the Nernst response with increasing temperature (Fig. S8f).

Text and figure changes in Part 8 of Supplementary material:

Hall angle is calculated by the formula $\tan\Theta = \sigma_{cb}/\sigma_{bb}$. Based on Mott relation and linear response equation, the diffusive Nernst contribution can be expressed as $S_{yx} = \pi^2/3(k_B^2T/e)\tan\Theta/\varepsilon$. Due to small Hall angle and the large Fermi energy (ε~500 meV), the diffusive Nernst component is negligibly small. The ambipolar Nernst component is obtained by subtracting diffusive contribution from the total signal. These results indicate that the ambipolar contribution dominates the large Nernst signal.

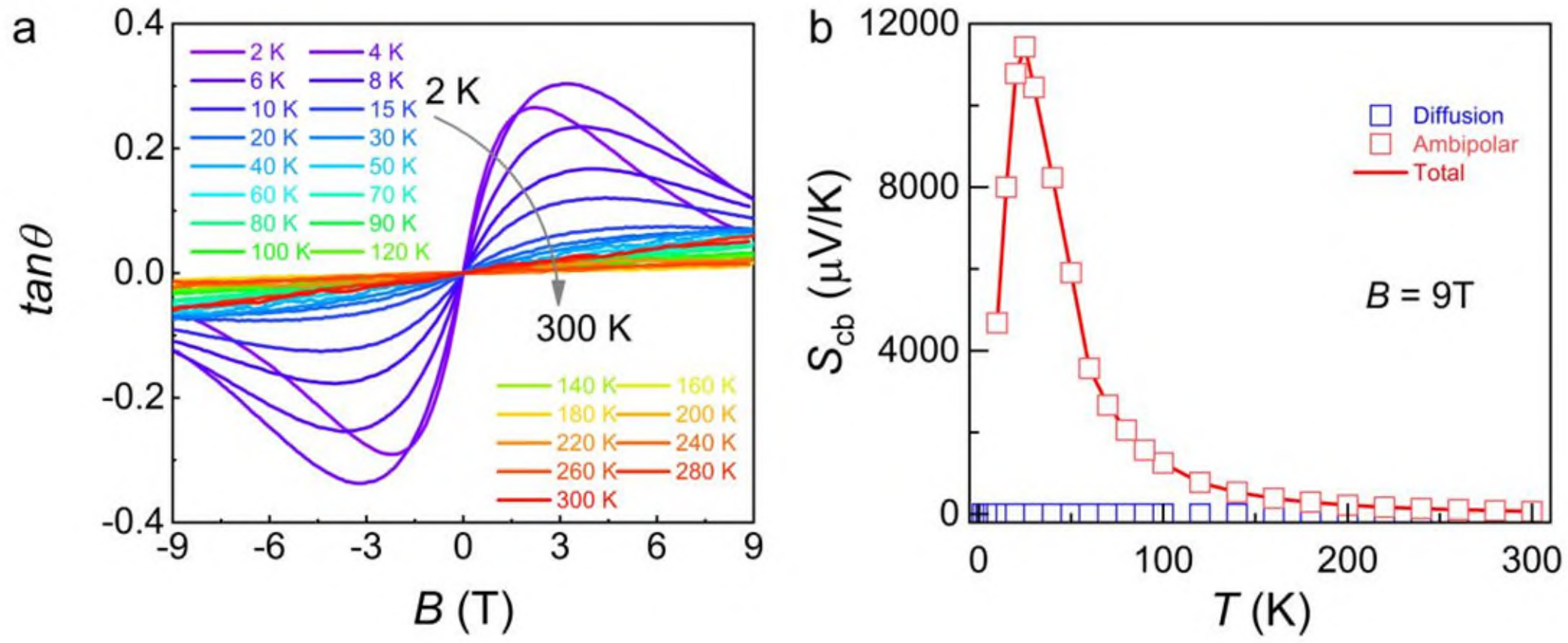

Fig. S7 | **a** The Hall angle σ_{cb}/σ_{bb} in the temperature range from 2 K to 300 K. **b** The quantitative analysis of the diffusion and ambipolar Nernst contributions. The diffusive component is obtained by the formula $S_{yx} = \pi^2/3(k_B^2T/e)\tan\Theta/\varepsilon$. The ambipolar contribution is obtained by subtracting diffusion contribution from the total signal.

Text and figure changes in Part 9 of Supplementary material:

For LMO, the partial diffusive Seebeck coefficients S_{diff}^{h} and S_{diff}^{e} (see Fig. S8a) are calculated

based on the wannier tight-binding model, which are constructed based on the DFT calculation using wannier90 package,[3-4] with the Mo-*d* orbitals included. Fig. S8b shows the temperature dependence of Seebeck coefficients measured along *b*- and *a*-axes. To obtain the total partial Seebeck coefficients, the reduced mobility is first calculated in Fig. S8c and the difference between total partial Seebeck coefficients (S_{total}^{h} - S_{total}^{e}) is then determined using Eq. (2). Combined with the temperature-dependent S_b (S_b = S_{total}^{h} + S_{total}^{e}; Fig. S8b), the resulting total partial Seebeck coefficients are shown in Fig. S8d. The phonon-drag partial Seebeck contributions are obtained by calculating the diffusion contributions from the total partial Seebeck coefficients. The Nernst signals arising from different contributions are further distinguished in Figs. S8e-f. Figure S8e demonstrates that, under quasi-one-dimensional conditions, the introduction of reduced mobility significantly enhances the Nernst signal compared to that based on the Hall mobility. Figure S8f separates the phonon-drag and diffusive contributions to the Nernst signal. At low temperatures, the Nernst response is dominated by phonon drag, whereas with increasing temperature, the diffusive contribution gradually becomes more significant.

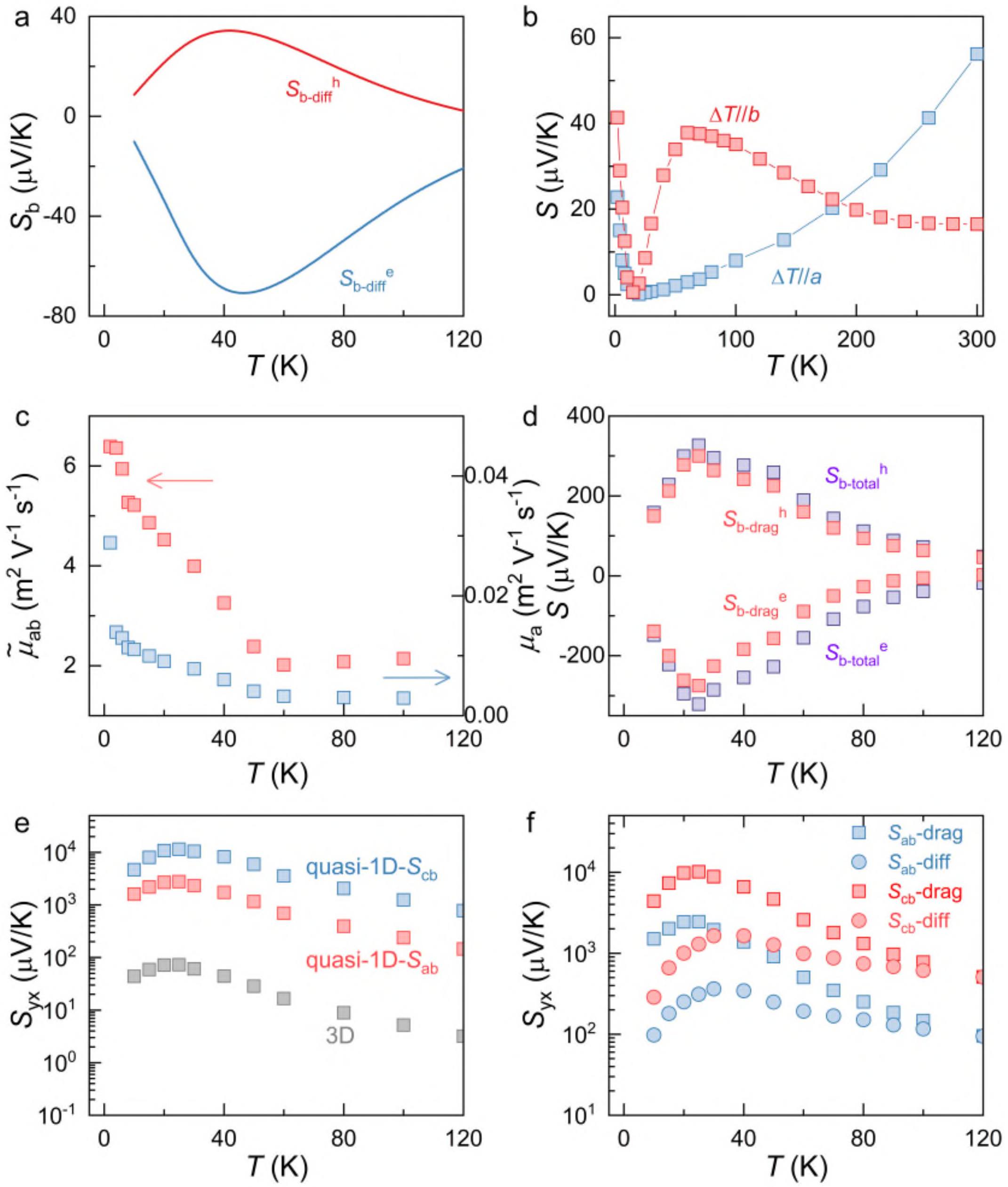

Fig. S8 | **a** Temperature dependence of the partial diffusive Seebeck coefficient, obtained through DFT calculations. **b** Temperature dependent Seebeck coefficients with the heat current applied along *b*- and *a*-axes, respectively. **c** Temperature dependence of the carrier mobility μ_a and the reduced mobility $\tilde{\mu}_{ab}$ (μ_{Hab}^2/μ_a). The μ_a is obtained through low field fitting of Fig. S4f. The Hall mobility μ_{Hab} is obtained through two-carrier model fitting. **d** Temperature dependence of the total partial Seebeck coefficients and the corresponding phonon-drag contributions. **e** The temperature-dependent Nernst signals S_{ab} and S_{cb} for quasi-one-dimensional LMO, compared with that of a three-dimensional case, where only the Hall mobility is considered and the reduced mobility is neglected. **f** Temperature-dependent Nernst signals in S_{ab} and S_{cb}, highlighting the respective contributions from phonon drag and diffusive Seebeck effects.

Comment 3.8

The paper claims exotic 1D surface states and unconventional Fermi surfaces. In principle 1D surface states are not 'exotic', they are something studied for decades now and their only 'exotic' feature are the sharp features in their DOS. On the other hand, it is not clear what is a Fermi surface for 1D as we only have 1D electronic structure (this again goes back to the point about more clarity in the introduction about the studied material). ***The main concern is that sharp features in the DOS for thermoelectrics was the topic of investigation for many years, but it yielded no such performance enhancement. It is not clear what is different in this current work and they allow for such extraordinary performance. See for example [J. Appl. Phys. 105, 034506 (2009); Physical Review B, 83, 245305, (2011)], where it is explained why such features are not as useful as suggested.*** *Figure S1 could support this; it is shown that by reducing Kt, essentially making the system more 1D, the Nernst thermopower only increases by 50% or so only. In that figure, it is also not clear why there is a maximum and not a monotonic increase as Kt reduces, making the system more 1D.* ***Furthermore, how valid is the constant relaxation time approximation? This is crucial, because if one considers energy dependent scattering (which is the more correct approach), where the scattering rates are proportional to the density of states, then the energy dependencies of the scattering times and DOS cancel out, which makes transport at first order independent of dimensionality, and the low-D argument cannot be valid.***

Reply: We thank the reviewers for your valuable suggestions and apologize for the lack of clarity in the Introduction. We have now clarified that the system under study is a quasi-one-dimensional electronic system embedded within a three-dimensional crystal structure, characterized by flat Fermi sheets, as illustrated in Fig. 2d and Fig. S5b.

We also appreciate the reviewer's concern regarding whether low dimensionality enhances the Seebeck coefficient and the corresponding longitudinal power factor since previous studies (*Ref. A*:*J. Appl. Phys. 105, 034506 (2009); Ref. B*:*Phys. Rev. B 83, 245305 (2011)*) didn't fully support such statement. Moreover, our earlier results didn't show the expected monotonic enhancement. The reviewer's comment motivated us to further investigate the role of anisotropy more carefully. First, we would like to clarify the previous reports. In *Ref. A*, the discussion primarily focuses on transport at the same Fermi energy close to the band edge within the ballistic regime. However, their results do not capture the pronounced enhancement of Seebeck near the band edge that is typically observed in Boltzmann transport regime. *Ref. B*, on the other hand, investigates silicon nanowires and reports an enhancement of Seebeck with decreasing wire diameter and carrier concentration although the power factor is reduced due to the suppressed conductivity. Both *Ref. A* and *Ref. B* discuss semiconducting system with a single type of carrier. In contrast, our results focus on the Nernst effect in a metallic system where only perfect carrier compensation was assumed. Nevertheless, for Seebeck and Nernst response, the Fermi energy remains a key parameter in determining the magnitude of thermoelectric response.

Starting from a 3D isotropic system, the Fermi pocket becomes progressively flattened as the system goes quasi-one-dimension (**see Fig. R3**). Correspondingly, the Fermi energy is significantly reduced due to the redistribution of carriers onto quasi-one-dimensional Fermi sheets. Consistent with this picture, the model should determine the Fermi energy self-consistently at fixed band filling, similar as the fixed carrier concentration in *Ref. B*. Otherwise the model fail to properly capture the effect of anisotropy as seen in *Ref. A* and our previous results. Under the constrain of fixed band filling, our results reveal a significant enhancement of the Seebeck coefficient with increasing anisotropy

(via decreasing interchain coupling $t_{x,z}$) as strong anisotropy reshapes the distribution of carriers and lower the Fermi energy as shown below. Distinct from the complicate fabrication process and extra boundary scattering in 1D nanowires, the one-dimensional conducting chain in 3D crystals offers an easy way to realize quasi-one-dimensional physics with flat Fermi sheets, enabled by the crystal symmetry and orbital hybridizations, offering a well-defined 1D electronic states and easily reproducible transport measurements.

For the Nernst response, the filling for electron and hole bands are fixed under the condition of perfect compensation. The calculations similarly shows an enhancement of Nernst signal with increasing anisotropy. Notably, the Fermi surface undergoes a Lifshitz transition from closed pockets to open sheets upon touching the boundary of the Brillouin zone. This transition substantially reshapes the Fermi surface, i.e., the distribution of charge carriers, rendering the notion of anisotropy less well-defined in this vicinity. This accounts for the sign change around $K_t \approx 0.5$, whereas the Nernst response grows monotonically with anisotropy on both sides of the transition. This also accounts for the maximum Nernst thermopower in our earlier results.

To assess the role of relaxation time, we further introduce a *DOS*-dependent relaxation time $\tau(E)=\tau_0 g^{-1}(E)$, which shows negligible changes. The compensation between *DOS* and *DOS*-dependent relaxation time maybe arise from the fundamental physical constraints, but rather from simplified assumptions for parabolic bands, which are not applicable to the present system.

Finally, we introduce an additional in-plane anisotropy, which further enhances the Nernst effect. In this case, a clear in-plane anisotropy response emerges with $S_{zy}>S_{xy}$ for $t_z<t_x$, reflecting the increased directional asymmetry.

Besides, the enhancement of transverse thermoelectric performance in our work only relies on a large partial Seebeck coefficient. Such behavior has been experimentally observed, for example, in bismuth nanowires (***Ref. R6***), where the Seebeck coefficient is enhanced with decreasing size. Moreover, when combined with the enhanced reduced mobility, the transverse power factor can be significantly improved. As described by Eq. S18:

$$PF_{yx} = S_{yx}^{\ 2}/\rho \approx S_{\text{ambipolar}}/\rho \approx 1/4ne\mu_H^{\ 4}/\mu_{yy}B^2(S_{xx}^{\ h}-S_{xx}^{\ e})^2 \approx 1/4n^2e^2\mu_H^{\ 4}\rho_{yy}B^2(S_{xx}^{\ h}-S_{xx}^{\ e})^2 \quad ,$$

the transverse power factor (with the *x*-direction along the conducting chain and heat flow direction) is proportional to ρ_{yy}. With decreasing dimensionality, the interchain band dispersion becomes flatter, leading to an increase in ρ_{yy}. Consequently, together with the enhanced Seebeck coefficient, the transverse thermoelectric performance is strongly amplified. Thus, different from longitudinal power factors, transverse power factors may be enhanced with reduced dimension.

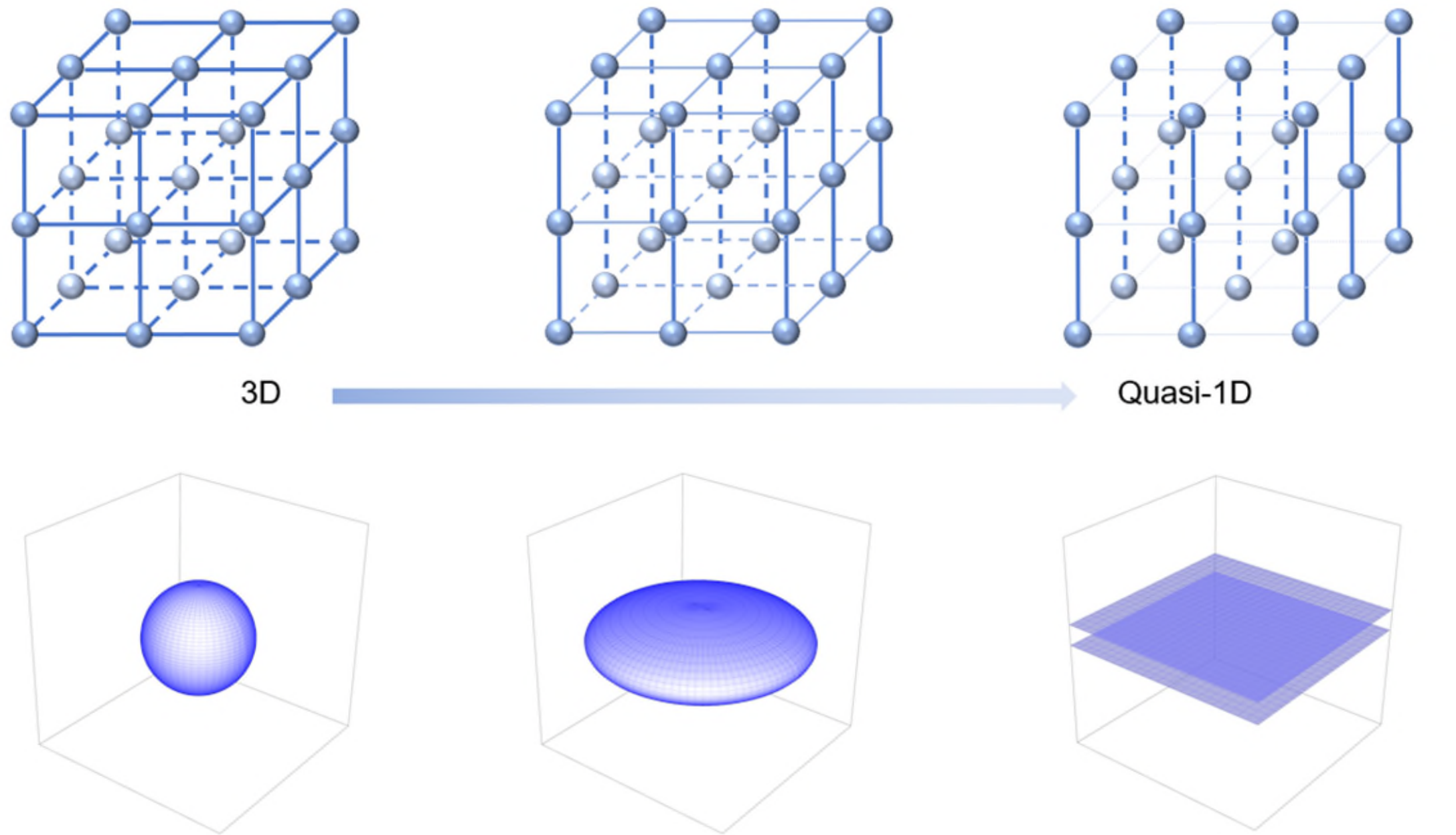

Fig. R3 | Evolution from three-dimensional to quasi-one-dimensional crystal structure and Fermi surface. Top: Schematic crystal structures illustrating the progressive reduction of interchain coupling, from an isotropic three-dimensional (3D) lattice to a quasi-one-dimensional (quasi-1D) system. **Bottom:** Corresponding evolution of the Fermi surface in momentum space. In the isotropic 3D case, the Fermi surface is closed and nearly spherical. With increasing anisotropy, it becomes progressively flattened and eventually intersects the Brillouin zone boundary, undergoing a Lifshitz transition. It ultimately evolves into an open, quasi-1D sheet-like Fermi surface.

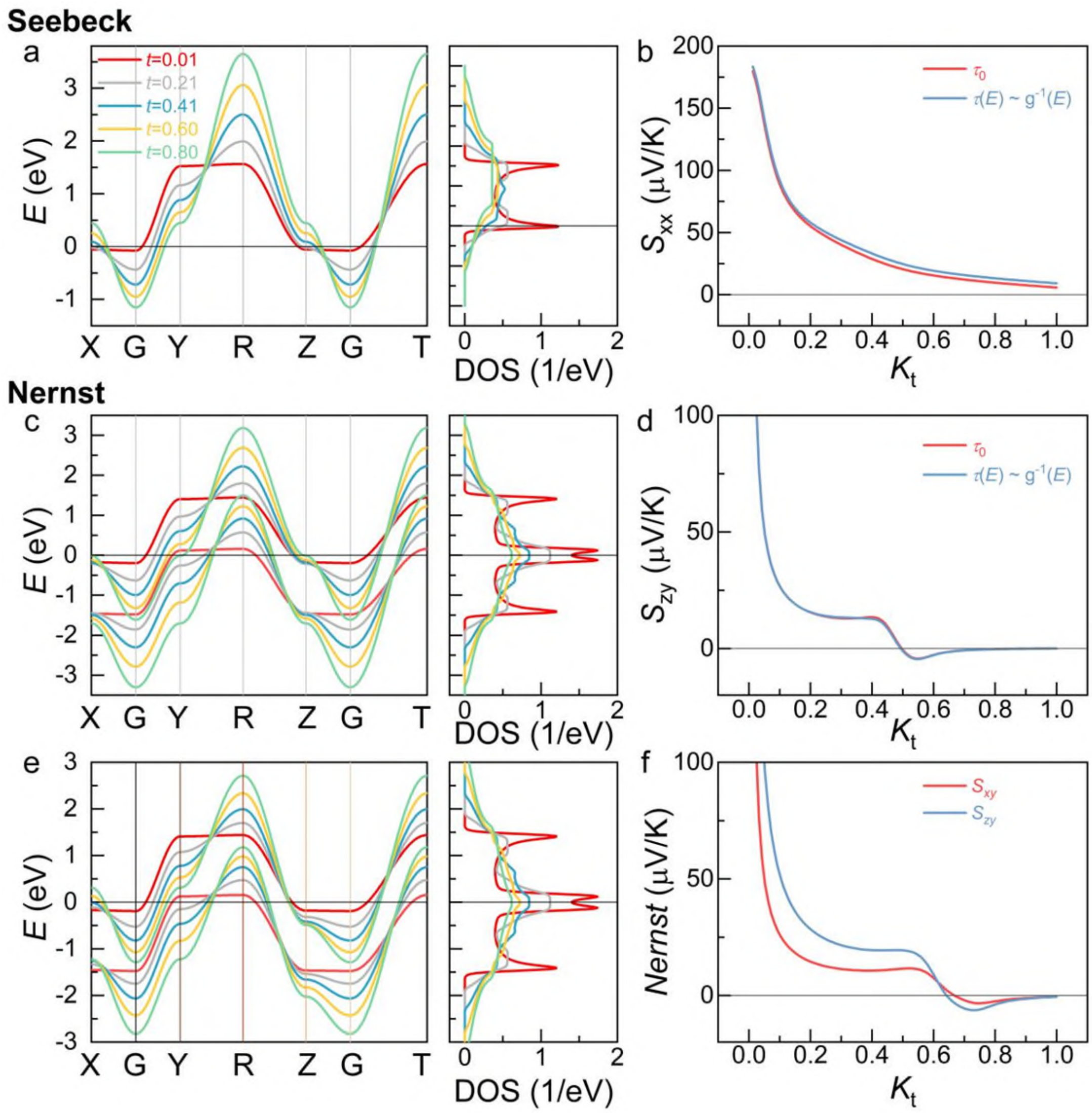

Fig. S1 | a-b Band structure, density of states for single band, and Seebeck response with varied anisotropy and same band filling at the Fermi level. **c-d** Band structure for compensated electron and hole bands, and corresponding Nernst reponse with t_x=t_z for constant relaxation time and *DOS*-dependent relaxation time. **e-f** Band structure for compensated electron and hole bands, and corresponding Nernst reponse with t_x=2t_z.

Reference

6. Heremans, J. P. et. al. Thermoelectric power of bismuth nanocomposites. *Phys. Rev. Lett.* **88**, 216801 (2002).

Text changes in the manuscript:

Line 63: In transverse TE geometry, the thermal gradient, electric current (or voltage), and magnetic field are mutually orthogonal (see Fig. 1a). This geometric decoupling of thermal and charge transport simplifies device structures[21-22] and introduces additional degrees of freedom for material optimization that are absent in longitudinal configurations. Large Nernst responses have been reported in various correlated and topological materials,[7-22] which are contributed by the phonon drag induced high partial Seebeck coefficients (S_{xx}^{h}; S_{xx}^{e}) as well as the high mobilities. Additional

underlying mechanisms between transverse geometry and enhanced TE effects, particularly in highly anisotropic systems remain insufficiently explored. Further enhancement of the Nernst thermopower naturally requires higher partial Seebeck coefficients. Quasi-one-dimensional (quasi-1D) conductors intrinsically host exotic electronic density of states and strongly anisotropic open Fermi surfaces, features that may profoundly reshape transverse TE responses. Developing a systematic framework that links quasi-1D electronic anisotropy and macroscopic TE performance is therefore essential for advancing both fundamental understanding and device design. In this work, we identify enhanced geometrical anisotropy as a guiding design principle for amplifying transverse TE responses. Through a combination of theoretical modelling and experimental investigation on bulk quasi-1D $Li_{0.9}Mo_6O_{17}$ (LMO), we show that low-dimensional structures not only generates a Seebeck-driven electric field larger than that in higher dimensional systems but also enhance the reduced mobility ($\tilde{\mu}$) through introducing anisotropic Fermi surface geometry and direction-dependent scattering. These effects act synergistically to yield large Nernst and Ettingshausen signals, exceeding state-of-the-art transverse TE materials. These findings establish geometrical optimization as a new framework for transverse TE engineering, opening avenues towards high-performance cryogenic cooling technologies.

Comment 3.9

It seems more reasonable that reduced mobilities are mostly responsible for the signals observed. In 3D systems we use the Hall mobility, but in 1D the reduced mobility, which makes the difference.

Reply: We thank the reviewers for your valuable suggestions. We agree that reduced mobility is one of the most important characteristic of low-dimensional systems. However, as the diffusive Seebeck calculation, the Seebeck coefficient can be significantly enhanced with decreasing interchain hoping t (reduced dimension). In additions, as indicated by the extended Nernst relation, $S_{yx}^{ambipolar} = 1/2(\boldsymbol{S}_{xx}^{h} - \boldsymbol{S}_{xx}^{e})B_z\boldsymbol{\mu}_H^2/\boldsymbol{\mu}_{yy}$, the partial Seebeck coefficients ($\boldsymbol{S}_{xx}^{h} - \boldsymbol{S}_{xx}^{e}$) and the reduced mobility ($\boldsymbol{\mu}_H^2/\boldsymbol{\mu}_{yy}$) are multiplicatively coupled. Therefore, both contributions are essential for achieving a large Nernst signal.

Comment 3.10

When comparing quasi-1D and 3D, why does this system has an advantage with regards to mobility, is not clear. At low temperatures phonon scattering is suppressed, but it is suppressed in all systems. Why is mobility higher in 1D than 3D?

Reply: We thank the reviewers for your valuable suggestions. We have carefully clarified the advantages of quasi-one-dimensional systems over three-dimensional systems in achieving large Nernst signals. In low-dimensional systems, the enhancement of the partial Seebeck coefficients together with reduced mobility jointly contributes to the large Nernst response.

We note that we do not explicitly distinguish the Hall mobility along the quasi-one dimensional chain direction from that in a isotropic three-dimensional system, as both can be relatively high at low temperatures due to suppressed phonon scattering. Such high mobility at low temperatures facilitates the emergence of large transverse thermoelectric responses, highlighting their potential for cryogenic cooling applications. However, for the reduced mobility term ${\mu^H_{yx}}^2/\mu_{yy}$ (with current applied along the chain direction x), a one-dimensional system ($\mu^H_{yx} >> \mu_{yy}$) exhibits a much larger value than a three-dimensional system ($\mu^H_{yx} \approx \mu_{yy}$) owing to its strong anisotropy.

Text changes in caption of Fig. 1d:

d Magnetic-field dependence of the Nernst thermopower in LMO at 25 K. Upon incorporating the contribution from dimensional reduction, the Nernst thermopower S_{ab} and S_{cb} are enhanced by factors of exceeding 22 and 160, respectively. Because phonon drag dominates the partial Seebeck coefficient at 25 K, this figure compares only the enhancement of the Nernst coefficient arising from the low dimensionality-enhanced reduced mobility. For 3D systems, the Nernst thermopower can be described by substituting the Hall mobility for the reduced mobility. In LMO, the extremely low interchain mobility (μ_a; μ_c) makes the reduced mobility much larger than Hall mobility, i.e., $\mu_{Hab}^2/\mu_a >> \mu_{Hab}$ and $\mu_{Hcb}^2/\mu_c >> \mu_{Hcb}$, leading to a substantially enhanced Nernst coefficient.

Comment 3.11

In Fig. S8 in the SI, the S increases with lowering T, and this is attributed to low dimensions, but this is a typical case also for the 3D materials. The authors should plot what a 3D model predicts for S, versus what an 1D model predicts, and also produce the same with constant and energy dependent relaxation time approximations. Only if the difference is substantial, they can claim that 'exotic' features are responsible substantially for the improvements they observe.

Reply: We are grateful to the reviewers for your valuable suggestions. We need to clarify the misleading for *S* increase with lowering *T*. This should not be attributed to low dimensions, which was caused by the asymmetric electronic states around the Fermi level as the thermal broadening of the Fermi surface changes. In the reply for Comment 3.8 and revised manuscript, we have included discussion of the anisotropy-dependent Seebeck coefficient. With increasing anisotropy (i.e., decreasing interchain coupling *t*), the Seebeck coefficient along the conducting chain is significantly enhanced, both when considering energy-dependent scattering and within the constant relaxation time approximation. Consequently, the dimension-reduction-induced increase in the partial Seebeck coefficient, together with the enhanced reduced mobility, leads to a substantial improvement in the transverse thermoelectric response. In addition, quantitative analyses of the low-dimensional Seebeck effect and phonon-drag contributions (Fig. S8f) further demonstrate the important role of low-dimensionality in enhancing the Seebeck coefficient, particularly at elevated temperatures.

List of changes:

1. We have substantially revised the discussion section to explicitly compare our work with previous reports and to highlight the advance of our work. We have added the reference ref.33 (*PRB* **108**, L100512 (2023)) in the manuscript.

2. We have added detailed descriptions of the thermoelectric measurements in the Methods section and provided schematic illustrations in Fig. S13.

3. We have included the total partial Seebeck coefficients in Fig. 2e and clarified how to obtain total, diffusion and phonon drag partial Seebeck coefficients. We first calculate the total partial Seebeck coefficients, S_{total}^{h} and S_{total}^{e}, through equation (2) in combination with transport measurements. We then obtain the diffusion components S_{diff}^{e} and S_{diff}^{h} through DFT calculations. Finally, the phonon-drag partial Seebeck coefficients are obtained by $S_{drag}^{e} = S_{total}^{e} - S_{diff}^{e}$ and $S_{drag}^{h} = S_{total}^{h} - S_{diff}^{h}$.

4. We have included a discussion comparing the electrical conductivity of LMO and Bi, along with future research directions aimed at further enhancing transverse thermoelectric signals and performance.

5. We have included a quantitative analysis separating the diffusive and ambipolar contributions (see Fig. S7b), which demonstrates the dominant role of the ambipolar component. In addition, We also have included the quantitative analyses of the low-dimensional Seebeck effect, anisotropic mobility (reduced mobility), and phonon-drag contributions in Figs. S8e-f.

6. We have corrected "amipolar" to "ambipolar" in the equation (2).

7. We have re-prepared the Fig. 1b. We have introduced the high-symmetry points Γ and Γ\`. The left side shows the dispersive band along the Γ-X direction, i.e., the quasi-one-dimensional chain direction, crossing the Fermi level at Γ\` point. The right side present the less dispersive band along the perpendicular Γ\`-Y\` direction, corresponding to the interchain direction. Near the Fermi level, the coexistence of a highly dispersive band along the chain direction and a nearly flat band along the interchain direction jointly dominates the transverse thermoelectric signals.

8. We have explicitly distinguished between the Fermi level and the Fermi energy in the revised manuscript. The Fermi level is defined as $E_F = 0$ meV in the plotted bands and Fermi surface, while the Fermi energy $\varepsilon \approx 500$ meV corresponds to the energy difference between the band bottom and the Fermi level (Fig. 2c).

9. We have added colored highlights to emphasize the bands along Γ-Y, Γ-X, and Γ-Z. To more directly visualize the band evolution near the Fermi energy along the *ac* plane, we additionally present band dispersions along Z1-Γ1-X1, Z2-Γ2-X2, Z3-Γ3-X3, and Z4-Γ4-X4.

10. We have re-prepared Fig. 1a to clearly distinguish the quasi-one-dimensional and three-dimensional wave packets, shown in blue and red, respectively. In addition, the directions of the one-dimensional chains and transport have been explicitly highlighted in the revised manuscript. Furthermore, we have clarified the differences in scattering between quasi-one-dimensional systems (characterized by strong anisotropy) and three-dimensional systems (typically isotropic).

11. We have re-organized the Introduction to emphasize that the system under study is quasi-one-dimensional, namely, a quasi-one-dimensional electronic system embedded within a three-dimensional crystal structure.

12. We have added the magneto-thermal conductivity data in Fig. S3c.

13. We have expanded the discussion of band compensation across the metal-insulator transition, as well as the validity of the two-carrier model fitting. In addition, we provide further details on the two-carrier fitting procedure.

14. We have included the different dimensional model predicts for S under considering energy-dependent scattering and within the constant relaxation time approximation. The dimensional reduction significantly enhance S.

15. We have remodeled the Nernst response as a function of K_t under considering energy-dependent scattering and within the constant relaxation time approximation. Under conditions of fixed band filling, the Nernst coefficient increases monotonically with decreasing K_t.

16. We have included the derivation of transverse power factor in equation s18. The transverse power factor is proportional to ρ_{yy}. With decreasing dimensionality, the interchain band dispersion becomes flatter, leading to an increase in ρ_{yy}. Consequently, together with the enhanced Seebeck coefficient, the transverse thermoelectric power factor is strongly amplified.

17. We have carefully clarified the difference of the reduced mobility ${\mu^{H}_{yx}}^2/\mu_{yy}$ between quasi-1D and 3D. The quasi-one-dimensional system ($\mu^{H}_{yx} >> \mu_{yy}$) exhibits a much larger value than a three-dimensional system ($\mu^{H}_{yx} \approx \mu_{yy}$) owing to its strong anisotropy.

Dear reviewers,

We sincerely thank the reviewers for your careful evaluation of our manuscript entitled "**Reduced dimensionality amplifies transverse thermoelectricity through enhanced Seebeck coefficient and reduced mobility**". We greatly appreciate your valuable and insightful comments. We believe that our detailed responses and the revised manuscript have adequately addressed your concerns and now meet the high standards of ***Nature Communications***.

Below please find the point-by-point response and the list of the changes that have been made in the revised manuscript.

Reviewer #1:

The authors have carefully revised the manuscript in response to my previous comments. My major concern was the novelty of this study, as the magnetotransport effects of this material have already been reported in two papers. In the rebuttal letter, the authors claimed that the central advance of this work is the establishment of a general physical framework that explains how reduced dimensionality amplifies transverse thermoelectric responses. In this case, the question is how broadly this framework can be applied. This issue was clarified to some extent by the comments of Referee 3. This study will attract attention in related fields and stimulate future research. Therefore, I recommend the publication of this manuscript.

Reply: We sincerely appreciate your constructive comments and the time you invested in reviewing our manuscript. Your valuable suggestions in the previous round of review greatly helped us improve the quality, clarity, and analysis of the manuscript. We highly appreciate your positive evaluation of the revised version and your recommendation for acceptance and publication.

Reviewer #2:

The authors' response to most comments is reasonable and satisfactory, except for Comment 2.1. The description of the analysis background and physical model should be both concise and comprehensive in the introduction of the main text, as the current manuscript remains poorly organized and ***relies too heavily on implicit references to the supplementary material.***

Reply: We thank the reviewer for this important comment and for the time and effort devoted to reviewing our manuscript. In the revised manuscript, we have incorporated the anisotropic Nernst model into the Introduction to provide a clearer theoretical framework. We have also clarified that the giant Nernst response in LMO is dominated by the ambipolar contribution.

Comment 2.1

For the revision, the concern regarding Comment 2.1 should be addressed.

The authors claim that Syx = Syx_ambipolar is justified because the partial conductivities of electrons and holes are close in value. However, in Eq. 1, the first term, (S_yx^e σ_yy^e + S_yx^h σ_yy^h)/σ_yy, cannot be simply canceled because there is no information provided on the S_yx values for electrons or holes. When the partial conductivities are similar, it reduces to S_yx^e + S_yx^h, not their difference.

Reply: We thank the reviewer for this important suggestion and agree with the reviewer's point. In the previous version, we directly estimated the first (diffusive) term using the total Hall angle, which

was not rigorous. In fact, the diffusive contribution should be evaluated separately for electrons and holes according to $S_{cb}^{Diffusion} = \pi^2/3(k_B{}^2T/e)(\tan\Theta^h-\tan\Theta^e)/\varepsilon$.

Accordingly, in the revised manuscript, we separately calculated the Hall angles of electrons and holes ($\sigma_{yx}{}^h = n_h e\mu_{Hh}{}^2B/(1+\mu_{Hh}{}^2B^2)$; $\sigma_{yx}{}^e = -n_e e\mu_{He}{}^2B/(1+\mu_{He}{}^2B^2)$) and then evaluated the diffusive contribution. As shown in the revised Fig. S7, the diffusive contribution is only ~10 μV/K at 25 K, which is three orders of magnitude smaller than the measured total Nernst thermopower (11470 μV/K).

We apologize for the unclear presentation of Fig. S7(b) in the previous version. To improve clarity, we have revised both the figure and the corresponding discussion in the Supplementary Material and the main text. These results demonstrate that the diffusive contribution is negligible, and therefore the total Nernst thermopower is well approximated by the ambipolar contribution, $S_{yx}{}^{total} = S_{yx}{}^{ambipolar}$.

Text and figure changes in Part 8 of Supplementary material:

The Hall angles of electrons and holes is calculated by the formula $\tan\Theta = \sigma_{cb}{}^e/\sigma_{bb}$ and $\tan\Theta = \sigma_{cb}{}^h/\sigma_{bb}$ with $\sigma_{yx}{}^h = n_h e\mu_{Hh}{}^2B/(1+\mu_{Hh}{}^2B^2)$ and $\sigma_{yx}{}^e = -n_e e\mu_{He}{}^2B/(1+\mu_{He}{}^2B^2)$). Based on Mott relation and linear response equation, the diffusive Nernst contribution can be expressed as $S_{yx}{}^{Diffusion} = \pi^2/3(k_B{}^2T/e)\tan\Theta/\varepsilon$ for single band system. Furthermore, the diffusive Nernst contribution can be expressed as $S_{yx}{}^{Diffusion} = \pi^2/3(k_B{}^2T/e)(\tan\Theta^h-\tan\Theta^e)/\varepsilon$ for two-band system. Although the Hall angle is relatively large, the Fermi energy is also comparatively high ($\varepsilon \approx 500$ meV). Consequently, the diffusive contribution is only ~10 μV/K at 25 K, which is three orders of magnitude smaller than the measured total Nernst thermopower (11470 μV/K). The ambipolar Nernst component is obtained by subtracting diffusive contribution from the total signal. These results indicate that the ambipolar contribution dominates the large Nernst signal.

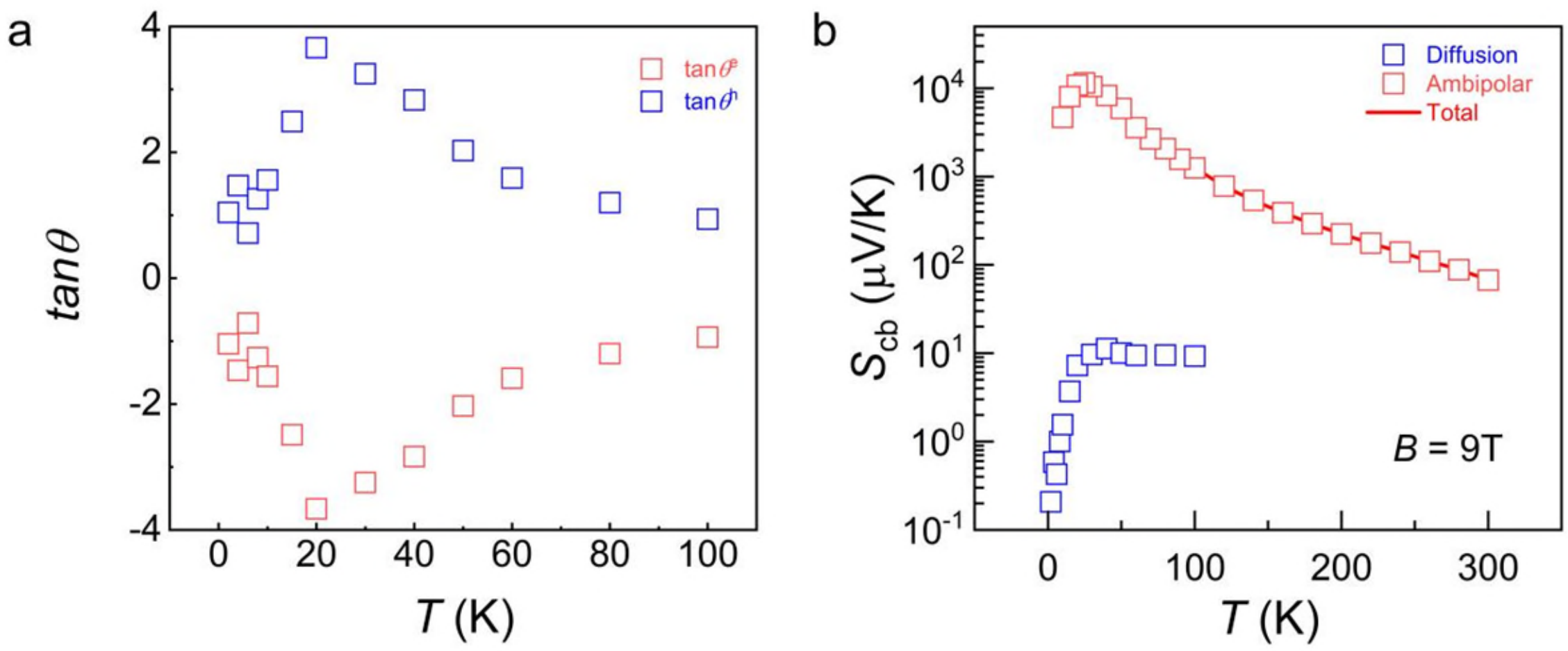

Fig. S7 | **a** Temperature-dependent Hall angles of electrons and holes ($\sigma_{cb}{}^e/\sigma_{bb}$; $\sigma_{cb}{}^h/\sigma_{bb}$) from 2 K to 100 K under a magnetic field of 9 T. **b** The quantitative analysis of the diffusion and ambipolar Nernst contributions. The diffusive component is obtained by the formula $S_{yx}{}^{Diffusion} = \pi^2/3(k_B{}^2T/e)(\tan\Theta^h-\tan\Theta^e)/\varepsilon$. The ambipolar contribution is obtained by subtracting diffusion contribution from the total signal.

Text changes in the manuscript:

Line 271: Although the Hall angle is relatively large, the Fermi energy is also comparatively high

($\varepsilon \approx 500$ meV). Consequently, the enhancement associated with the large Hall angle is largely offset by the high Fermi energy, resulting in a carrier-diffusion contribution of only ~10 μV/K at 25 K. This value is nearly three orders of magnitude smaller than the measured Nernst thermopower of 11,470 μV/K. Therefore, the giant Nernst response is dominated by the ambipolar contribution (see Fig. S7b).

Comment 2.2

The authors should therefore clearly explain the magnitude and estimation of S_yx for both electrons and holes, and organize the introduction to provide the necessary physical context.

Reply: We thank the reviewer for this important suggestion. As described in our response to Comment 2.1, we have separately calculated the electron and hole contributions to the Nernst thermopower S_{yx}. In addition, we have reorganized the Introduction to improve its logical flow, and the anisotropic Nernst model is now presented in detail in the main text to provide a clearer theoretical framework for the subsequent discussion.

Text changes in the manuscript:

Line 133: To quantitatively evaluate the role of reduced dimensionality, we employ a tight-binding model described by $H_{\pm} = c_0 - t_x\cos k_x - t_y\cos k_y - t_z\cos k_z \pm \Delta$, where t_i represents hopping parameters, Δ controls the energy separation between the electron and hole bands, and c_0 adjusts the band filling and carrier compensation. Because the multiband Nernst response is highly sensitive to both the difference between the electron and hole Seebeck coefficients and the degree of carrier compensation, we impose perfect compensation between the two bands with identical carrier fillings for all anisotropy ratios (Figs. S1c-d). This construction eliminates variations arising from carrier imbalance, allowing the effect of dimensionality-induced Fermi-surface reconstruction to be isolated. We first fix t_y and set $t_x = t_z = K_t t_y$ with K_t varying from 1 to 0 to model the dimensional crossover from 3D to 1D. For each K_t, the Fermi energy is determined self-consistently at fixed carrier filling, and the Nernst thermopower is calculated by solving the Boltzmann transport equation. As shown in Fig. 1c, the Nernst response increases monotonically with decreasing K_t, demonstrating that the evolution toward a more anisotropic Fermi surface geometry can substantially amplify the Nernst response. To further distinguish the contribution of transport anisotropy from that of the Seebeck coefficient, we introduce an additional anisotropy by setting $t_x = 2t_z$. For the same longitudinal Seebeck coefficient S_{yy}, S_{zy} exhibits a much stronger enhancement than S_{xy}, indicating that the enhanced Nernst response cannot be attributed solely to an increased Seebeck coefficient. Instead, the anisotropic Fermi-surface geometry enhances the Nernst response through the reduced mobility. Within this framework, the Nernst thermopower is governed by the dimensionality-dependent parameters $S_{xx}^{h} - S_{xx}^{e}$ and K_m. Reduced dimensionality therefore enhances the transverse thermoelectric response through two synergistic mechanisms: increasing the partial Seebeck coefficient and strengthening transport anisotropy, thereby providing additional degrees of freedom for boosting the Nernst response.

Comment 2.3

If S_yx for electrons and holes are both large, their sum may be significant and possibly overestimated even with a small difference in conductivities. Thus, a proper estimation of S_yx for electrons and holes is mandatory to justify their framework.

Reply: We thank the reviewer for this important suggestion. As shown in Fig. S7b, the diffusive contribution is much smaller than the ambipolar contribution. Therefore, the discussion framework adopted in the present manuscript is well justified, with the Nernst response being dominated by the ambipolar contribution.

Reviewer #3:

The authors have addressed some of my concerns, but still some minor issues remain before publication:

Reply: We thank the reviewer for the considerable time and effort devoted to improving our manuscript. In the revised version, we have carefully addressed all the comments and made the corresponding modifications accordingly.

Comment 3.1

In line 131, they authors need to specify how they solve the Boltzmann transport equation, and specifically what they assume for the scattering times and scattering processes.

Reply: We thank the reviewer for the important suggestion. We have added details on how the Boltzmann transport equation is solved in Part 2 of the Supplementary Material. For $\tau=\tau_0 g^{-1}(\varepsilon)$, it is assumed to be proportional to density of state, which may be relevant to short-range impurity scattering with weak momentum-dependence. This should be considered as a simplified phenomenological model rather than a universal scattering mechanism, since realistic scattering can depend on momentum, band index and microscopic defects.

Text and figure changes in Part 2 of Supplementary material:

The thermoelectric transport is evaluated based on Boltzmann transport equation

$$\frac{\partial f}{\partial t}+\dot{r}\cdot\nabla_r f+\dot{k}\cdot\nabla_k f=\left(\frac{\partial f}{\partial t}\right)_{coll} \tag{s19}$$

with $\dot{r}=v_{nk}$ and $\hbar\dot{k}=-e(E+v_{nk}\times B)$.

Under relaxation time approximation we have $\left(\frac{\partial f}{\partial t}\right)_{coll}=-\frac{f-f_0}{\tau_{nk}}$, with f_0 the equilibrium Fermi-Dirac distribution and τ_{nk} the relaxation time. Under weak electric field and temperature gradient, the distribution is linearized as $f=f_0+f_1$, with f_1 the first-order deviation from equilibrium. Under steady condition, $\frac{\partial f}{\partial t}=0$. Therefore, the Boltzmann equation is reduced to

$$v_{nk}\cdot\nabla_r f_0-eE\cdot v_{nk}\frac{\partial f_0}{\partial \varepsilon}-\frac{e}{\hbar}(v_{nk}\times B)\cdot\nabla_k f_1=-\frac{f_1}{\tau_{nk}}, \tag{s20}$$

since $\nabla_k f_0=\frac{\partial f_0}{\partial \varepsilon}\nabla_k\varepsilon=\hbar v_{nk}\frac{\partial f_0}{\partial \varepsilon}$. For $\nabla_r f_0=\frac{\partial f_0}{\partial T}\nabla T+\frac{\partial f_0}{\partial \mu}\nabla\mu=(-\frac{\partial f_0}{\partial \varepsilon})(\nabla\mu+\frac{\varepsilon-\mu}{T}\nabla T)$, the BTE can be further reduced to

$$[1+\tau_{nk}\Omega]f_1=\tau_{nk}v_{nk}\cdot F(-\frac{\partial f_0}{\partial \varepsilon}) \tag{s21}$$

Where $\Omega=-\frac{e}{\hbar}(v_{nk}\times B)\cdot\nabla_k$, and the generalized force $F=-\nabla\mu-\frac{\varepsilon-\mu}{T}\nabla T-eE$. At weak magnetic fields, the distribution can be obtained as

$$f_1=[1+\tau\Omega]^{-1}\tau v_j\cdot F\left(-\frac{\partial f_0}{\partial\varepsilon}\right)$$
$$\approx[1-\tau\Omega]\tau v_j\cdot F\left(-\frac{\partial f_0}{\partial\varepsilon}\right)$$
$$=\tau F\left(-\frac{\partial f_0}{\partial\varepsilon}\right)\left[v_j-\tau\Omega v_j\right]$$
$$=\tau F\left(-\frac{\partial f_0}{\partial\varepsilon}\right)\left[v_j+e\tau\epsilon_{lmn}v_l B_m\frac{\partial v_j}{\partial k_n}\right] \quad \text{(s22)}$$

assuming k-independent relaxation time $\tau_{nk}=\tau$. Consider $J=\sigma E+\alpha\nabla T+\dots$, the electric and thermoelectric conductivity then can be obtained as

$$\sigma_{ij}=\frac{e^2}{V}\int\tau v_i(v_j+e\tau\epsilon_{lmn}v_l B_m\frac{\partial v_j}{\partial k_n})(-\frac{\partial f}{\partial\varepsilon})d\varepsilon \quad \text{(s23)}$$

$$\alpha_{ij}=\frac{e}{TV}\int\tau v_i(v_j+e\tau\epsilon_{lmn}v_l B_m\frac{\partial v_j}{\partial k_n})(\varepsilon-\mu)(-\frac{\partial f}{\partial\varepsilon})d\varepsilon. \quad \text{(s24)}$$

within constant relaxation time approximation $\tau_{nk}=\tau$.

Notably, for $\tau=\tau_0 g^{-1}(\varepsilon)$, it is assumed to be proportional to density of state, which may be relevant to short-range impurity scattering with weak momentum-dependence. This should be considered as a simplified phenomenological model rather than a universal scattering mechanism, since realistic scattering can depend on momentum, band index and microscopic defects.

Comment 3.2

The RTA in Fig. 1 should be noted as CRTA as is customary written, because RTA can be perceived as allowing energy dependence. Still, it is not clear why there is a difference between the RTA and the DPA, and which one is the appropriate one for this system?

Reply: We thank the reviewer for pointing this out. We acknowledge that the term "RTA" imply an energy-dependent relaxation time. To avoid ambiguity, we have replaced "RTA" by "CRTA (constant relaxation time approximation)" throughout Fig. 1 and the corresponding text.

In the revised manuscript, the theoretical model has now been incorporated into the main text to provide a clearer framework for the subsequent discussions. We fix the electron and hole bands to remain perfectly compensated with identical carrier fillings for different anisotropy. This enables us to isolate the intrinsic effect of reduced dimensionality on the Nernst response. We first fix t_y and set $t_x = t_z = K_t t_y$ with K_t varying from 0 to 1 to model the dimensional crossover from 1D to 3D. The Nernst response increases markedly with decreasing K_t ($K_t \propto K_m$), whereas introducing a *DOS*-dependent relaxation time produces only negligible changes (Fig. 1c). These results demonstrate that anisotropic Fermi surface geometry (K_m) alone can strongly enhance the Nernst response. To further distinguish the role of transport anisotropy from that of the Seebeck coefficient, we additionally consider the case of $t_x = 2t_z$. Under the same longitudinal Seebeck coefficient S_{yy}, S_{zy} exhibits a much stronger enhancement than S_{xy}, demonstrating that the transport anisotropy induced by reduced dimensionality provides an additional and substantial enhancement of the Nernst response beyond the Seebeck effect alone. Within this framework, the Nernst thermopower is governed by the dimensionality-dependent parameters K_m and S_{xx}^h - S_{xx}^e. Therefore, the enhancement mechanism discussed in this work does not rely on introducing anisotropic scattering. Since any specific anisotropic-scattering model cannot be regarded as universally applicable, we have removed the discussion of the scattering from the revised manuscript, including DPA.

Text changes in the manuscript:

Line 133: To quantitatively evaluate the role of reduced dimensionality, we employ a tight-binding model described by $H_{\pm} = c_0 - t_x\cos k_x - t_y\cos k_y - t_z\cos k_z \pm \Delta$, where t_i represents hopping parameters, Δ controls the energy separation between the electron and hole bands, and c_0 adjusts the band filling and carrier compensation. Because the multiband Nernst response is highly sensitive to both the difference between the electron and hole Seebeck coefficients and the degree of carrier compensation, we impose perfect compensation between the two bands with identical carrier fillings for all anisotropy ratios (Figs. S1c-d). This construction eliminates variations arising from carrier imbalance, allowing the effect of dimensionality-induced Fermi-surface reconstruction to be isolated. We first fix t_y and set $t_x = t_z = K_t t_y$ with K_t varying from 1 to 0 to model the dimensional crossover from 3D to 1D. For each K_t, the Fermi energy is determined self-consistently at fixed carrier filling, and the Nernst thermopower is calculated by solving the Boltzmann transport equation. As shown in Fig. 1c, the Nernst response increases monotonically with decreasing K_t, demonstrating that the evolution toward a more anisotropic Fermi surface geometry can substantially amplify the Nernst response. To further distinguish the contribution of transport anisotropy from that of the Seebeck coefficient, we introduce an additional anisotropy by setting $t_x = 2t_z$. For the same longitudinal Seebeck coefficient S_{yy}, S_{zy} exhibits a much stronger enhancement than S_{xy}, indicating that the enhanced Nernst response cannot be attributed solely to an increased Seebeck coefficient. Instead, the anisotropic Fermi-surface geometry enhances the Nernst response through the reduced mobility. Within this framework, the Nernst thermopower is governed by the dimensionality-dependent parameters $S_{xx}^h - S_{xx}^e$ and K_m. Reduced dimensionality therefore enhances the transverse thermoelectric response through two synergistic mechanisms: increasing the partial Seebeck coefficient and strengthening transport anisotropy, thereby providing additional degrees of freedom for boosting the Nernst response.

Changes in Fig. 1c:

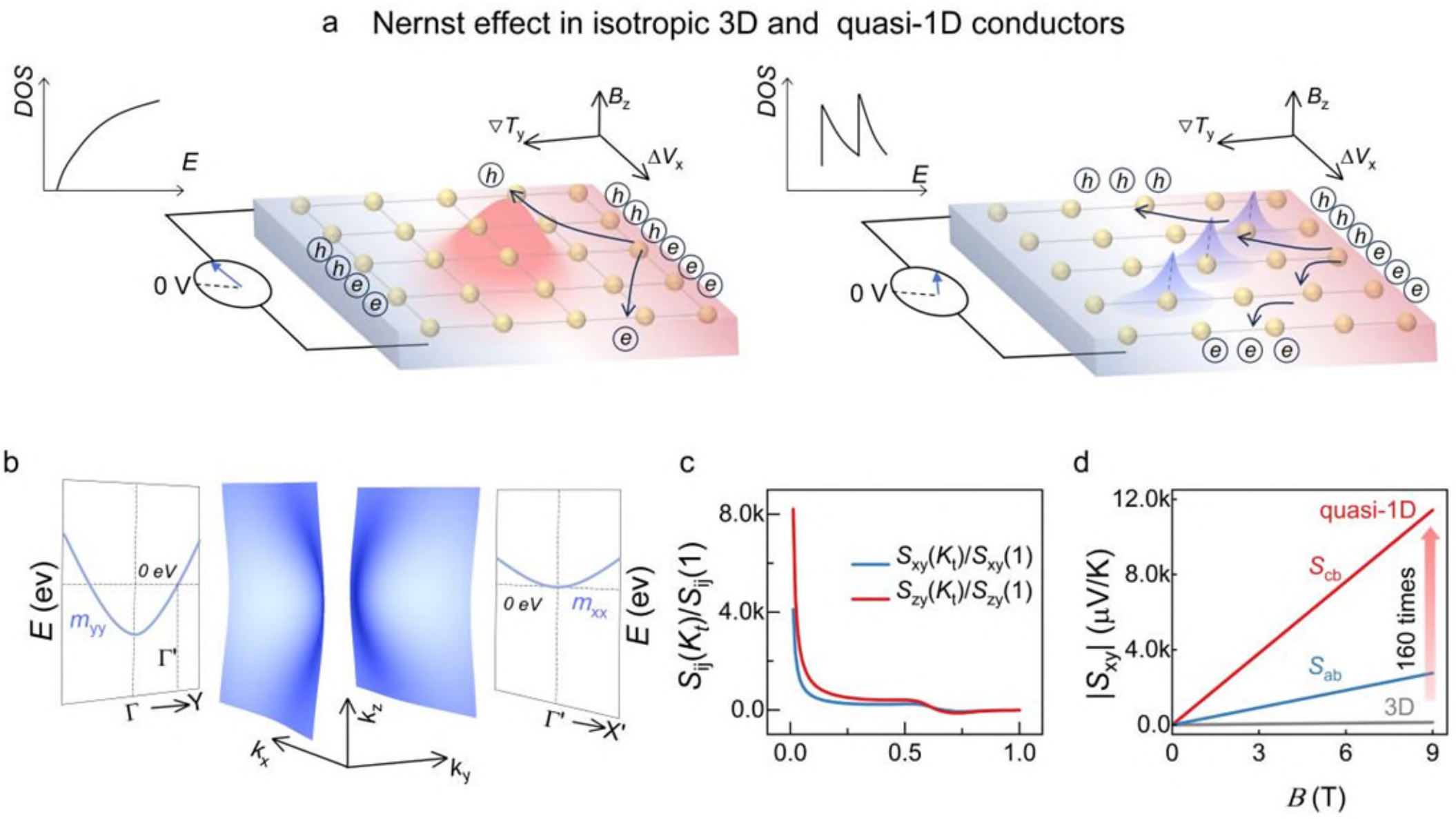

c Calculated Nernst thermopower within the constant relaxation-time approximation (CRTA) for t_x

$= 2t_z$. The Nernst thermopower increases with decreasing K_t ($K_t \propto K_m$, $K_m = m_{xx}(m_{zz})/m_{yy}$). For the same longitudinal Seebeck coefficient S_{yy}, S_{zy} exhibits a much larger enhancement than S_{xy}, demonstrating that, beyond the Seebeck coefficient, transport anisotropy through the reduced-mobility term can provide an additional and substantial enhancement of the Nernst response.

Comment 3.3

Check the notation in Fig. 1b the Y direction is towards the flat direction, whereas in 2c it is in the linear direction with the Fermi level crossing the band, not below the band.

Reply: We thank the reviewer point this out. In the previous revision, the Y direction of Fig. 1b is towards the flat direction and Fig. 2c shows the Band structure along chains (Γ-Y) with a sharp quasi-linear band in the vicinity of the Fermi level E_F. To avoid confusion, we have revised the manuscript to ensure consistency throughout. The chain direction is now uniformly defined as the *y* direction in the entire manuscript.

Comment 3.4

The legends of Fig. 2e are wrong. They are all squares, but the data symbols are triangles.

Reply: We apologize for this mistake. The legends of Fig. 2e have been corrected in the revised manuscript.

Changes in Fig. 2e

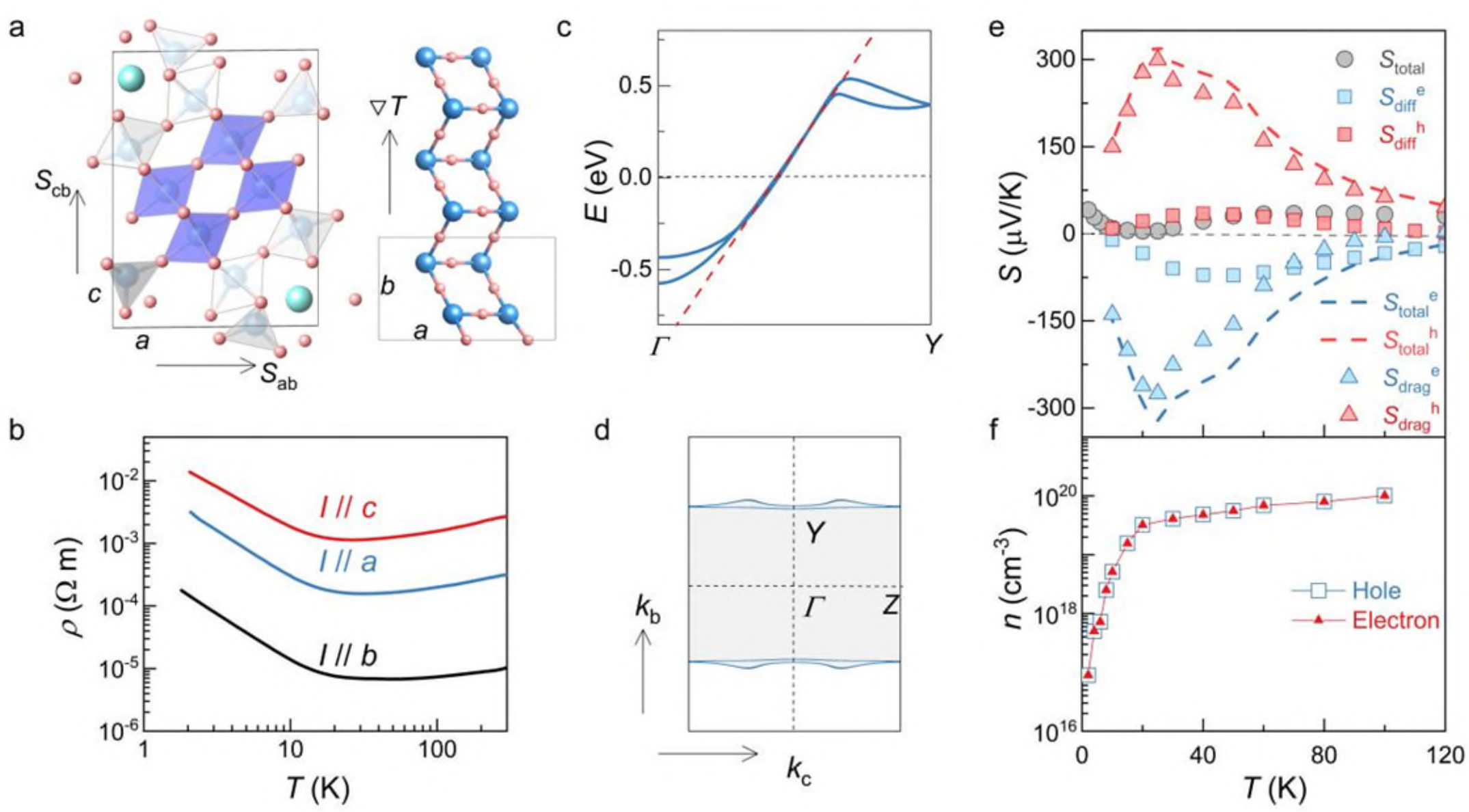

Comment 3.5

Line 321: the PF performance is large, but it is not better than other materials as shown in Fig. 4d. The authors should smoothen their claims.

Reply: We thank reviewer for point this out. We have smoothen the manuscript "Specifically, PF_{cb} attains 814 μW cm^{-1} K^{-2} at the same temperature and field, comparable to those of other reported transverse TE materials, including WTe_2 and $NbSb_2$".

Changes in manuscript:

Line 337 : power factor. Specifically, PF_{cb} attains 814 μW cm^{-1} K^{-2} at the same temperature and field, comparable to those of other reported transverse TE materials, including WTe_2 and $NbSb_2$.

Comment 3.6

The term 'reduced dimensionality' in the title tends to point towards the 1D DOS features. But the effect here observed is mostly due to the transport anisotropy. It is better is this is reflected in the title, rather than 1D. Maybe 'reduced dimensionality transport anisotropy'. In the text it needs to be stated clearly: is it the 1D features that have some effect, or mostly the transport directionality. If both have an effect, then quantify how much of an effect each has. This point is still not clear.

Reply: We thank reviewer for your valuable suggestions. Reduced dimensionality may more readily evoke low-dimensional density-of-states effects rather than anisotropic transport, we have revised the title accordingly following your suggestion, and the new title is "Observation of the amplified transverse thermoelectric signals by reduced-dimensionality transport anisotropy".

As described by the extended Nernst formula, $S_{xy}^{ambipolar} = 1/2(S_{yy}^{h} - S_{yy}^{e})B_z\mu_H^2/\mu_{xx}$, both the partial Seebeck coefficients ($S_{yy}^{h} - S_{yy}^{e}$) and the reduced mobility (μ_H^2/μ_{xx}) play essential roles in generating a large Nernst signal, with a multiplicative coupling between them. Therefore, both contributions are crucial (Fig. S8e-f). However, the enhancement of the partial Seebeck coefficient arising from the low-dimensional density of states is mainly effective at high temperatures, whereas the phonon-drag contribution dominates the partial Seebeck coefficient at low temperatures ($S_{bb}^{drag} \approx S_{bb}^{diffusion}$ at 120 K). Furthermore, transport anisotropy provides a further enhancement of the Nernst signal through the reduced mobility term. Specifically, the calculated ratio (S_{cb}^{1D}/S_{cb}^{3D} = 160 at 25 K) reaches approximately 160, highlighting the dominant role of transport anisotropy in amplifying the transverse thermoelectric response. These contributions of the quasi-iD features and transport anisotropy have now been explicitly discussed in the revised manuscript to avoid ambiguity regarding their respective roles.

Changes in Manuscript:

Line 282: At low temperatures, the phonon-drag Seebeck component dominates, particularly near the phonon peak. As the temperature increases, the contribution from the Seebeck effect induced by the low-dimensional density of states gradually becomes more significant ($S_{bb}^{drag} \approx S_{bb}^{diffusion}$ at 120 K; see Fig. S8f). Moreover, quasi-1D transport anisotropy further enhance Nernst thermopower via a extended Nernst equation (2) (see Fig. S8e), in which the reduced mobility $\tilde{\mu}$ embodies both intrinsic contributions from Fermi surface curvature K_m.

Comment 3.7

The abstract is also somewhat deceiving. Although the signals can be some of the highest in their temperature range, the PF, which is the important quantity, is the one to be also mentioned, and that is comparable to the best materials measured in the literature (not highest). This needs to be smoothened.

Reply: We thank reviewer for your important suggestions. We have added expression about *PF* "The large Nernst response further leads to a high transverse power factor of 814 μW cm^{-1} K^{-2} at 25 K and 9 T, comparable to those of the best transverse thermoelectric materials."

Changes in Abstract of manuscript:

Line 34: The large Nernst response further leads to a high transverse power factor of 814 μW cm^{-1} K^{-2} at 25 K and 9 T, comparable to those of the best transverse thermoelectric materials.

List of changes:

1. We have introduced the anisotropic Nernst model into the main text.
2. We have clarified how the Boltzmann transport equation is solved.
3. We have removed the discussion related to DPA scattering.
4. We have unified the axis definitions in the schematic diagrams and model descriptions, consistent with the convention used for LMO.
5. The legend of Fig. 2e has been corrected in the revised manuscript.
6. The manuscript has been revised for clarity in the sentence: "Specifically, PF_{cb} attains 814 μW cm^{-1} K^{-2} at the same temperature and field, comparable to those of other reported transverse TE materials, including WTe_2 and $NbSb_2$." We have also added a discussion of the power factor (*PF*) in the Abstract.
7. The title has been revised following the reviewer's suggestion to: "Observation of amplified transverse thermoelectric signals via reduced dimensionality transport anisotropy." In addition, we have further clarified the respective roles of the low-dimensional Seebeck contribution and the reduced mobility term in enhancing the Nernst response in the main text.

Sincerely,

Claudia Felser (on behalf of all co-authors)

Max Planck Institute for Chemical Physics of Solids, Dresden, Germany

Dear reviewers,

We sincerely thank the reviewers for your careful evaluation of our manuscript entitled "**Observation of the amplified transverse thermoelectric signals by reduced dimensionality**". We greatly appreciate your valuable and insightful comments. We believe that our detailed responses and the revised manuscript have adequately addressed your concerns and now meet the high standards of ***Nature Communications***. Below please find the point-by-point response.

Reviewer #2 (Remarks to the Author):

The authors' response has addressed my concerns. The manuscript clearly demonstrates the high thermoelectric power and its origin due to low dimensionality.
Thus, I recommend its publication.
For better readability, it would be helpful if the authors could relate Sxy and S_xy^ambipolar (as well as the diffusion term), not only by text but also by equations.
Reply: We are very grateful to Reviewer #2 for recommending publication. We also thank the reviewer for providing many valuable comments in the last two rounds of review, which help us to clarify some most important points and to improve our manuscript.
To quantitatively clarify the different contributions to the transverse thermoelectric response, we have provided the expressions for the diffusion and ambipolar terms: $Sxy^{\mathrm{Diffusion}} = \pi^2/3(k_B^2 T/e)(\tan\Theta^h - \tan\Theta^e)/\varepsilon$ and $S_{xy}^{\mathrm{ambipolar}} = 1/2(S_{yy}^{h} - S_{yy}^{e})B_z\mu_H^2/\mu_{xx}$. Using the experimentally relevant parameters for LMO, we find that the diffusion contribution is negligibly small compared with the experimentally observed transverse thermoelectric signal. In contrast, the ambipolar contribution is sufficiently large and accounts for the dominant part of the transverse thermoelectric response. These results further support our conclusion that the giant Nernst signal in LMO primarily originates from the ambipolar contribution associated with its highly anisotropy.

Reviewer #3 (Remarks to the Author):

The authors have responded to my concerns adequately and I recommend publication.
The only remaining issue is that many symbols in the equations in the main paper but mostly in the SI do not appear correctly, but I would assume that these will be fixed during the proof stage.
Reply: We are very grateful to Reviewer #3 for recommending publication. We sincerely appreciate the reviewer's valuable comments and constructive suggestions throughout the two rounds of review, which have helped us clarify several important points and further improve the quality and presentation of our manuscript.
We also appreciate the reviewer for pointing out the issue with the formatting of symbols in the equations, particularly those in the Supplementary Information. We have carefully checked and corrected all equation symbols to ensure that they are displayed properly and consistently.

Sincerely,

Claudia Felser (on behalf of all co-authors)

Max Planck Institute for Chemical Physics of Solids, Dresden, Germany
